# Preparing the 2025 revision of the International Code of Nomenclature of Prokaryotes

**DOI:** 10.1099/ijsem.0.006666

**Published:** 2025-01-29

**Authors:** Aharon Oren, David R. Arahal, Henrik Christensen, Markus Göker, Célia M. Manaia, Edward R.B. Moore

**Affiliations:** 1 Institute of Life Sciences, The Hebrew University of Jerusalem, The Edmond J. Safra Campus, 9190401 Jerusalem, Israel; 2 Departamento de Microbiología y Ecología, Edificio de Investigación, Campus de Burjassot, Universidad de Valencia, 46100 Burjassot, Valencia, Spain; 3 Department of Veterinary Animal Sciences, University of Copenhagen, 4 Stigbøjlen, DK-1870 Frederiksberg C, Denmark; 4 Leibniz Institute DSMZ – German Collection of Microorganisms and Cell Cultures, Inhoffenstrasse 7B, 38124 Braunschweig, Germany; 5 CBQF- Centro de Biotecnologia e Química Fina– Laboratório Associado, Escola Superior de Biotecnologia, Universidade Católica Portuguesa, Rua Arquiteto Lobão Vital, Apartado 2511, 4202-401 Porto, Portugal; 6 Department of Infectious Disease, Institute for Biomedicine, Sahlgrenska Academy, University of Gothenburg, P.O. Box 7193, SE-402 34 Göteborg, Sweden

**Keywords:** International Code of Nomenclature of Prokaryotes, International Committee on Systematics of Prokaryotes, the Prokaryotic Code

## Abstract

The editorial Board of the *International Code of Nomenclature of Prokaryotes* (ICNP) – the Prokaryotic Code – has compiled already ratified proposed emendations of the ICNP, together with additional editorial changes and clarifications. These were implemented in a draft 2025 revision of the *Prokaryotic Code*. To comply with Articles 13(b)(4) and 4(d) of the statutes of the International Committee on Systematics of Prokaryotes (ICSP), a public discussion of the document will start on 1 January (or later if required) 2025, to last for 6 months. Here, we present the basis for the revision and the procedure for the discussion. The discussion will be followed by the balloting of the ICSP members.

The latest version of the *International Code of Nomenclature of Prokaryotes* (ICNP) – the *Prokaryotic Code* – [1] was revised in 2022 [1], after 14 years had passed since the previous revision [2]. Article 13 of the statutes of the ICSP [3] states that an Editor-in-Chief, appointed by the Executive Board of the ICSP, shall be responsible for the continuing revision of the ICNP, for its editing and its publication. The editorial board of the ICNP, consisting of A. Oren (appointed Editor-in-Chief); *ex officio* members D.R. Arahal (Chair of the Judicial Commission), H. Christensen (Vice-Chair of the Judicial Commission), E.R.B. Moore (Chair, ICSP) and C.M. Manaia (Vice-Chair, ICSP) and co-opted member M. Göker, has prepared a new revision of the ICNP. It incorporates the seven proposals for emendation published in the *International Journal of Systematic and Evolutionary Microbiology* (IJSEM) and approved by the ICSP between December 2022 and December 2025 (Table 1), as well as newly proposed minor editorial changes and further additions to clarify the meaning of the ICNP. The proposed 2025 revision is found as a supplementary file together with the text of the 2022 revision and comments explaining the changes proposed by the editorial board.

**Table 1.** Proposals for emendation of the ICNP approved by the ICSP published in the IJSEM between December 2022 and November 2024

No.	Authors	Title	Reference	Proposing modification of
1	Oren A, Arahal DR, Göker M, Moore ERB, Rossello-Mora R, *et al.*	Proposals to emend Rules 8, 15, 22, 25a, 30(3)(b), 30(4) and 34a and Appendix 7 of the International Code of Nomenclature of Prokaryotes	[4]	Rules 8, 15, 22, 25a, 30(3)(b), 30(4) and 34a, Appendix 7
	Oren A	Emendation of Rules 8, 15, 22, 25a, 30(3)(b), 30(4) and 34a and Appendix 7 of the International Code of Nomenclature of Prokaryotes	[5]	
2	Göker, M, Oren A	Proposal to include the categories kingdom and domain in the International Code of Nomenclature of Prokaryotes	[6]	Principle 8, Rules 5b, 8, 15 and 33a, Appendix 7
	Oren A	Emendation of Principle 8, Rules 5b, 8, 15 and 33a and Appendix 7 of the International Code of Nomenclature of Prokaryotes to include the categories of kingdom and domain	[7]	
3	Oren A, Chuvochina M	Naming genera after geographical locations; proposal to emend Appendix 9 of the International Code of Nomenclature of Prokaryotes	[8]	Appendix 9
	Oren A	Emendation of Appendix 9 of the International Code of Nomenclature of Prokaryotes to provide guidelines for naming genera after geographical locations	[9]	
4	Arahal DR, Bisgard M, Christensen H, Clermont D, Dijkshoorn L, *et al.*	The best of both worlds: a proposal for further integration of *Candidatus* names into the International Code of Nomenclature of Prokaryotes	[10]	Rules 66–73
5	Pallen MJ	Use of connecting vowels after stems ending in the same vowel: a proposal to emend Appendix 9 of the International Code of Nomenclature of Prokaryotes	[11]	Appendix 9
	Oren A	Emendation of Appendix 9 of the International Code of Nomenclature of Prokaryotes to regulate the use of connecting vowels in compound names after stems ending in the same vowel	[12]	
6	Pallen MJ	Formation of prokaryote names from personal names: a review of current practice and a proposal to emend Appendix 9 of the International Code of Nomenclature of Prokaryotes	[13]	Recommendation 6, Rule 64, Appendix 9
7	Oren A, Kostovski M, Pallen MJ	Proposal to add a Note to Rule 8 of the International Code of Nomenclature of Prokaryotes to clarify the meaning of the term ‘stem’	[18]	Rule 8*

* Approval by the ICSP pending.

Major changes have been made in Principle 8 and Rules 5b, 6, 7, 15, 22, 25, 30, 33, 34 and 64 [4,13]. Some of these changes implement the decision to include the ranks of kingdom and domain in the *Code* [6,7]. An entirely new section (Section 10, Rules 66–73) was added to regulate the status of *Candidatus* names, which were formerly not governed by the rules of the ICNP [10]. Major updates were made in Appendices 4, 5, 7 and 9 [4,9,11]. Minor updates were introduced in other appendices, as required. Opinions 123–130 issued by the ICSP Judicial Commission [14,17] were added in Appendix 5. Many sections of Appendix 9 – Orthography were rewritten and are presented here, as approved by the ICSP in separate ballots [8,9, 11,13]. The new version of this appendix should be useful for assisting authors in proposing correctly formed names that comply with the rules of the *Code.*

In addition to these already approved ICNP emendations, a proposal to clarify the meaning of the term ‘stem’ [18], which is awaiting approval by the ICSP, has been provisionally added. Minor editorial changes have also been made throughout the document. In addition, further emendations are proposed to clarify outstanding issues, most of which reflect previous decisions of the Judicial Commission and its guidelines [19]. Care has been taken to ensure that these emendations do not actually change the status quo. The proposed emendations include the addition of examples and the replacement or removal of outdated examples; clarification of the status of cyanobacterial names with a category not recognized by the ICNP, based on considerations from Opinion 129 [16], as well as issues of formation, conservation and rejection of such names; clarification of certain borderline cases regarding effective publications; the addition of sentences to clarify interrelationships within the ICNP; the rephrasing of sentences that might be considered to contradict other statements in the ICNP [19]; the addition of sentences to better reflect established practice regarding the conservation and rejection of names or epithets by the Judicial Commission [19,21]; and, finally, the addition of sentences to further clarify the intended use of terms such as ‘basonym’ [19].

A 6-month period of public discussion will start on the date this announcement is published in the IJSEM, to comply with Articles 13(b)(4) and 4(d) of the statutes of the ICSP [3]. All relevant documents have been posted on the ICSP website (http://the-icsp.org) and on the Slack communication platform (https://icnp-revision.slack.com), which contains the discussion forum. Colleagues who wish to participate in the discussions on the Slack platform should contact A. Oren (aharon.oren@mail.huji.ac.il), D.R. Arahal (david.ruiz@uv.es), M. Göker (markus.goeker@dsmz.de), or E.R.B. Moore (erbmoore@ccug.se) to be registered in the Slack workspace. Following an up to 2-month period, during which the editorial board members may respond to the comments raised, the material will be sent for balloting among the full and co-opted members of the ICSP.

## Supplementary material

10.1099/ijsem.0.006666 Uncited Supplementary Material 1.

## Abbreviations

ICNP: International Code of Nomenclature of Prokaryotes
ICSP: International Committee on Systematics of Prokaryotes
IJSEM: International Journal of Systematic and Evolutionary Microbiology

## References

[1] Oren A, Arahal DR, Göker M, Moore ERB, Rossello-Mora R (2023). International code of nomenclature of prokaryotes. Int J Syst Evol Microbiol.

[2] Parker CT, Tindall BJ, Garrity GM (2019). International Code of Nomenclature of Prokaryotes. Prokaryotic Code (2008 revision). Int J Syst Evol Microbiol.

[3] Whitman WB, Bull CT, Busse H-J, Fournier P-E, Oren A (2019). Request for revision of the Statutes of the International Committee on Systematics of Prokaryotes. Int J Syst Evol Microbiol.

[4] Oren A, Arahal DR, Göker M, Moore ERB, Rossello-Mora R (2023). Proposals to emend Rules 8, 15, 22, 25a, 30(3)(b), 30(4), 34a, and Appendix 7 of the International Code of Nomenclature of Prokaryotes. Int J Syst Evol Microbiol.

[5] Oren A (2023). Emendation of Rules 8, 15, 22, 25a, 30(3)(b), 30(4), 34a, and Appendix 7 of the International Code of Nomenclature of Prokaryotes. Int J Syst Evol Microbiol.

[6] Göker M, Oren A (2023). Proposal to include the categories kingdom and domain in the International Code of Nomenclature of Prokaryotes. Int J Syst Evol Microbiol.

[7] Oren A (2023). Emendation of Principle 8, Rules 5b, 8, 15, 33a, and Appendix 7 of the International Code of Nomenclature of Prokaryotes to include the categories of kingdom and domain. Int J Syst Evol Microbiol.

[8] Oren A, Chuvochina M (2023). Naming genera after geographical locations. Proposal to emend Appendix 9 of the International Code of Nomenclature of Prokaryotes. Int J Syst Evol Microbiol.

[9] Oren A (2024). Emendation of Appendix 9 of the International Code of Nomenclature of Prokaryotes to provide guidelines for naming genera after geographical locations. Int J Syst Evol Microbiol.

[10] Arahal D, Bisgaard M, Christensen H, Clermont D, Dijkshoorn L (2024). The best of both worlds: a proposal for further integration of *Candidatus* names into the International Code of Nomenclature of Prokaryotes. Int J Syst Evol Microbiol.

[11] Pallen MJ (2023). Use of connecting vowels after stems ending in the same vowel: a proposal to emend Appendix 9 of the International Code of Nomenclature of Prokaryotes. Int J Syst Evol Microbiol.

[12] Oren A (2024). Emendation of Appendix 9 of the International Code of Nomenclature of Prokaryotes to regulate the use of connecting vowels in compound names after stems ending in the same vowel. Int J Syst Evol Microbiol.

[13] Pallen MJ (2024). Formation of prokaryote names from personal names: a review of current practice and a proposal to emend Appendix 9 of the International Code of Nomenclature of Prokaryotes. Int J Syst Evol Microbiol.

[14] Arahal DR, Bull CT, Busse H-J, Christensen H, Chuvochina M (2022). Judicial Opinions 123-127. Int J Syst Evol Microbiol.

[15] Arahal DR, Bull CT, Christensen H, Chuvochina M, Dedysh SN (2023). Judicial Opinion 128. Int J Syst Evol Microbiol.

[16] Arahal DR, Bull CT, Christensen H, Chuvochina M, Dedysh SN (2024). Judicial Opinion 129. Int J Syst Evol Microbiol.

[17] Arahal DR, Bull CT, Christensen H, Chuvochina M, Dunlap C (2024). Judicial Opinion 130. Int J Syst Evol Microbiol.

[18] Oren A, Kostovski M, Pallen MJ (2024). Proposal to add a Note to Rule 8 of the International Code of Nomenclature of Prokaryotes to clarify the meaning of the term ‘stem’. Int J Syst Evol Microbiol.

[19] Arahal DR, Bull CT, Busse H-J, Christensen H, Chuvochina M (2023). Guidelines for interpreting the International Code of Nomenclature of Prokaryotes and for preparing a Request for an Opinion. Int J Syst Evol Microbiol.

[20] Arahal DR, Busse H-J, Bull CT, Christensen H, Chuvochina M (2022). Judicial Opinions 103–111. Int J Syst Evol Microbiol.

[21] Judicial Commission (1985). Judicial Opinion 60: Rejection of the name *Yersinia pseudotuberculosis* subsp. *pestis* (van Loghem) Bercovier *et al*. 1981 and conservation of the name *Yersinia pestis* (Lehmann and Neumann) van Loghem 1944 for the plague bacillus. Int J Syst Bacteriol.

