# Supplementary material for: Preparing the 2025 revision of the International Code of Nomenclature of Prokaryotes
Source: Int J Syst Evol Microbiol. 2025 Jan 29;75(1):006666. doi: 10.1099/ijsem.0.006666 (PMC12282017; doi:10.1099/ijsem.0.006666)
Supplement: Uncited Supplementary Material 1. [file ijsem-75-06666-s001.pdf]

Changes approved by the ICSP or soon to be approved are highlighted grey.

Other changes proposed in the text to be printed are highlighted yellow.

International Code of Nomenclature of Prokaryotes. Prokaryotic Code (2025 Revision)

Aharon Oren[1], David R. Arahal[2], Henrik Christensen[3] Markus Göker[4], Célia M. Manaia[5], and Edward R. B. Moore[6] (Editors)

[1]Institute of Life Sciences, The Hebrew University of Jerusalem, The Edmond J. Safra Campus, 9190401 Jerusalem, Israel
[2]Departamento de Microbiología y Ecología, Universitat de València, 46100 Burjassot (Valencia), Spain
[3] Department of Veterinary and Animal Sciences, University of Copenhagen, Stigbøjlen 4, 1870 Frederiksberg C, Denmark
[4]Leibniz Institute DSMZ – German Collection of Microorganisms and Cell Cultures, Department of Bioinformatics and Databases, Inhoffenstrasse 7B, 38124 Braunschweig, Germany
[5] Universidade Católica Portuguesa, CBQF, Centro de Biotecnologia e Química Fina, Laboratório Associado, Escola Superior de Biotecnologia, Rua de Diogo Botelho 1327, 4169-005, Porto, Portugal
[6]Department of Infectious Disease and Culture Collection University of Gothenburg (CCUG), Institute for Biomedicine, Sahlgrenska Academy, University of Gothenburg, SE-402 34 Gothenburg, Sweden

Corresponding Author: Aharon Oren (aharon.oren@mail.huji.ac.il)

PREFACE

To be completed by the editors after approval of the 2025 Revision by the ICSP

CHAPTER 1. GENERAL CONSIDERATIONS

General Consideration 1

The progress of prokaryotic microbiology is advanced by a precise and standardized system of nomenclature accepted by the international community of microbiologists.

General Consideration 2

Scientific names must be regulated by internationally accepted Rules, to achieve and maintain order in nomenclature.

General Consideration 3

The Rules that govern the nomenclature used in the biological sciences are embodied in International Codes of Nomenclature (see Appendix 1 for a list of these Codes).

General Consideration 4

Rules of nomenclature do not govern the delimitation of taxa nor determine their relations. The Rules prescribe the procedures for creating and proposing new names and for assessing the correctness of the names applied to defined taxa.

General Consideration 5

This *Code of Nomenclature of Prokaryotes* applies to all Prokaryotes. The nomenclature of eukaryotic microbial groups is provided for by other Codes: fungi and algae by the *International Code of Nomenclature for algae, fungi, and plants*; protozoa by the *International Code of Zoological Nomenclature*. The nomenclature of viruses is provided for by the *International Code of Virus Classification and Nomenclature* (see Appendix 1)

Note. "Prokaryotes" covers those organisms that are variously recognized as e.g., *Archaea*, *Archaebacteria*, *Archaeobacteria*, *Bacteria*, *Cyanobacteria*, *Cyanophyceae*, *Eubacteria*, *Schizomycetes*, and *Schizophycetes*.

If a taxon originally assigned to the *Cyanophyceae/Cyanobacteria* was named under the provisions of the *International Code of Nomenclature for algae, fungi, and plants*, any of its names need satisfy only the requirements of that Code for status equivalent to valid publication under the *International Code of Nomenclature of Prokaryotes*, unless otherwise expressly stated in the Rules.

General Consideration 6

This *Code* is divided into Principles, Rules and Recommendations.

(1) The *Principles* (Chapter 2) form the basis of the *Code*, and the Rules and Recommendations are derived from them.

(2) The *Rules* (Chapter 3) are designed to make the Principles effective, to reassess the nomenclature of the past and to provide for the nomenclature of the future.

(3) The *Recommendations* (Chapter 3) deal with subsidiary points and are appended to the Rules that they supplement. Recommendations do not have the force of Rules; they are intended to be guides to desirable practice in the future. Names contrary to a Recommendation cannot be replaced or rejected for this reason.

(4) Provisions for emendations of Rules, for special exceptions to Rules, and for interpretation of the Rules have been made by the establishment of the International Committee on Systematics of Prokaryotes (ICSP) and its Judicial Commission, which acts on behalf of the ICSP (see Rule 1b and Statutes of the International Committee on the Systematics of Prokaryotes). Opinions issued by the Judicial Commission become effective as provided for in the ICSP Statutes. The official journal of the ICSP is the *International Journal of Systematic and Evolutionary Microbiology* (IJSEM), formerly *International Journal of Systematic Bacteriology* (IJSB), formerly the *International Bulletin of Bacteriological Nomenclature and Taxonomy* (IBBNT). (Some other journal could be specified by the ICSP if required. Such possible future specification is implicit in the use of "*International Journal of Systematic and Evolutionary Microbiology*" or "IJSEM" throughout this Code, but is not always repeated at each mention.)

(5) *Appendices* are added to assist in the application of this *Code* (see Table of Contents).

(6) Definitions of certain words used in the *Code* are provided. Such words are indicated in boldface type in the clause concerned, and they may be printed in boldface type elsewhere in this *Code*.

(7) The *Notes* added to General Considerations, Principles, Rules and Recommendations are intended to clarify the preceding text and are an integral part of the corresponding text.

General Consideration 7

Nomenclature deals with the following:

(1) Terms used to denote the **taxonomic categories**, e.g., "species", "family" and "phylum".

(2) Relative ranks of the categories (see Rule 5).

(3) Names applied to individual taxa. A taxonomic group is referred to throughout this Code as a **taxon**, plural **taxa**.

"**Taxonomic group**" is used in this Code to refer to any group of organisms treated as a named group in a taxonomy; it may or may not correspond to a category.

Examples: Name of a species, *Pseudomonas* (generic name) *aeruginosa* (specific epithet); name of a genus, *Pseudomonas*; name of a family, *Pseudomonadaceae*; name of an order, *Pseudomonadales*.

General Consideration 8

The *International Code of Nomenclature of Prokaryotes* is an instrument of scientific communication. Names have meaning only in the context in which they were formed and used.

CHAPTER 2. PRINCIPLES

Principle 1

The essential points in nomenclature are to:

(1) Aim at stability of names.

(2) Avoid or reject the names that cause error or confusion.

(3) Avoid the useless creation of names.

(4) Nothing in this *Code* may restrict the freedom of taxonomic thought or action.

Note. "**Name**" in this *Code*, unless otherwise indicated, is used to refer to names applied to prokaryotes that have been validly published, whether legitimate or illegitimate (see Chapter 3, Section 3).

Principle 2

The nomenclature of prokaryotes is not independent of botanical and zoological nomenclature. When naming new taxa in the rank of genus or higher, due consideration is to be given to avoiding names that are regulated by the *International Code of Zoological Nomenclature* and the *International Code of Nomenclature for algae, fungi, and plants*.

Note. This principle takes effect with publication of acceptance of this change by the ICSP (from 1 January 2001) and is not retroactive.

For information about lists of names of zoological and botanical taxa, see Appendix 3.

Principle 3

The names of all taxa are Latin or Latinized words treated as Latin, regardless of their origin. They are usually taken from Latin or Greek (see Chapter 3, Section 9, and Appendix 9).

Principle 4

The purpose of giving a name to a taxon is to supply a means of referring to it rather than to indicate the characters or the history of the taxon.

Principle 5

The application of the names of taxa is determined by means of nomenclatural types, referred to in this *Code* as types (see Chapter 3, Section 4).

Principle 6

The correct name of a taxon is based upon **valid publication, legitimacy** and **priority of publication** (see Chapter 3, Section 5).

Principle 7

A name of a taxon has no status under the Rules and no claim to recognition unless it is validly published (see Chapter 3, Section 5; for pro-status, see Section 10).

Principle 8

Each taxon with a given **circumscription, position**, and **rank** can bear only one correct name, i.e., the earliest that is in accordance with the Rules of this *Code*. Provision has been made for exceptions to this Principle (see Rules 23a and 23b).

Note 1. The name of a species is a binary combination of generic name and specific epithet.

Note 2. (i) **Circumscription** of the taxon is an indication of its limits; (ii) **position** of a taxon is an indication in which higher taxon it is placed (see also Rule 23a); and (iii) **rank** of the taxon is its level in the hierarchical sequence of taxonomic categories.

Principle 9

The name of a taxon must not be changed without sufficient reason; if necessary, changes must be based upon further taxonomic studies or on the necessity of expunging a name that is contrary to the Rules of this *Code*.

CHAPTER 3. RULES OF NOMENCLATURE WITH RECOMMENDATIONS

Section 1. General

Rule 1a

This revision of the *International Code of Nomenclature of Prokaryotes* supersedes all previous revisions of the *Bacteriological Code* and the *International Code of Nomenclature of Prokaryotes* (see Appendix 1). It shall be cited as the *Prokaryotic Code* (2025 Revision) and will apply from the date of publication online.

Rule 1b

Alterations to this *Code* can be made only by the ICSP. Proposals for modifications, if any, must-be made as specified in the Statutes of the ICSP.

Rule 2

The Rules of this *Code* are retroactive, except where specified.

Examples: Rule 18a, Rule 30.

Rule 3

Names contrary to a Rule cannot be maintained, except that the ICSP, on the recommendation of the Judicial Commission, may make exceptions to the Rules (see Rule 23a).

Rule 4

In the absence of a relevant Rule or where the consequences of a Rule are uncertain, a summary in which all pertinent facts are outlined should be submitted to the Judicial Commission for consideration (see Appendix 8 for preparation of a Request for an Opinion).

Section 2. Ranks of Taxa

Rule 5a

Definitions of the taxonomic categories may vary with individual opinion, but the relative order of these categories may not be altered in any classification.

Rule 5b

The taxonomic categories above and including subspecies, which are covered by these Rules, are given below in ascending taxonomic rank. Those in the left column should be recognized; those in the right column should be considered optional. The Latin equivalents are given in parentheses.

	Subspecies (*Subspecies*)
Species (*Species*)	
	Subgenus (*Subgenus*)

Genus (*Genus*)	
	Tribe (*Tribus*)
Family (*Familia*)	
	Suborder (*Subordo*)
Order (*Ordo*)	
	Subclass (*Subclassis*)
Class (*Classis*)	
Phylum (*Phylum*)	
Kingdom (*Regnum*)	
Domain (*Dominium*)	

Rule 5c

Editorial Note. The former Rule 5c has been deleted. This rule only remains here as a placeholder, in order to avoid renumbering Rules 5d and above. Rule 5c should not be cited.

Rule 5d

Taxa below the rank of subspecies (**infrasubspecific subdivisions**) are not covered by the Rules of this *Code*, but see Rule 14a and Appendix 10.

Section 3. Naming of Taxa

General

Rule 6

The scientific names of all taxa must be treated as Latin; names of taxa above the rank of species are single words.

When proposing new names, the etymology must be provided.

With effect from 1 January 2023, names that end on -*myces*, -*phyces*, -*phyta*, or -*virus* must not be used, to avoid confusion with the names of eukaryotic or virus taxa. This restriction is not retroactive.

Recommendation 6

To form new prokaryotic names and epithets, authors are advised as follows:

(1) Avoid names or epithets that are long or difficult to pronounce.

(2) Make names or epithets that have an agreeable form that is easy to pronounce when Latinized.

(3) Words from languages other than Latin or Classical Greek should be avoided if equivalents exist in Latin or Classical Greek or can be constructed by combining word elements from these two languages.

Exceptions: names derived from typical local items, such as food, drink or geographical localities for which no Latin or Greek names exist.

(4) Do not adopt unpublished names or epithets found in authors' notes, without the authors' approval.

(5) The Greek K and Z and the Medieval Latin J (for consonantic I) may be maintained to avoid confusion.

Examples: *Actinokineospora*, *Flectobacillus major*.

(6) The abbreviation M.L. stands for 'Medieval Latin' not 'Modern Latin'. For the latter, N.L. ('Neo Latin') should be used.

(7) Authors should not name organisms after themselves or co-authors.

Names of Taxa above the Rank of Genus up to and including Order

Rule 7

The name of a taxon above the rank of genus, up to and including order, is a noun or an adjective used as a noun of Latin or Classical Greek origin or a Latinized word. It is in the feminine gender, the plural number, and written with an initial capital letter.

Example: Family *Pseudomonadaceae*.

Names of Taxa above the Rank of Order

Rule 8

The name of each taxon above the rank of order is a Latin or Latinized word.

The name of a domain (dominion) is in the plural number and written with an initial capital letter. The sole component of the name is the nominative plural of a word that is the last component of the name of a genus, whether it is the type genus of the domain or any other genus placed within the domain at the time of valid publication of its name.

The name of a kingdom is in the masculine gender, the plural number, and written with an initial capital letter. The name is formed by the addition of the suffix –*ati* to the stem of the name of the designated type genus.

The name of a phylum is in the neuter gender, the plural number, and written with an initial capital letter. The name is formed by the addition of the suffix –*ota* to the stem of the name of the designated type genus. The Judicial Commission can make exceptions regarding the use of the ending –*ota* when forming the name of a phylum.

The name of a class is in the plural number, and written with an initial capital letter.

Until 31 December 2011, new names of classes that were considered to have been validly published (see Rule 27) prior to or on that date were to be formed preferably in conformity with Recommendation 6.

With effect from 1 January 2012, for new names of classes that are considered to have been validly published (see Rule 27) on or after that date, the name is in the neuter gender and is

formed by the addition of the suffix –*ia* to the stem of the name of the type genus of the type order of the class.

The name of a subclass is in the feminine gender, plural number, and written with an initial capital letter. The name is formed by the addition of the suffix –*idae* to the stem of the name of the type genus of the type order of the subclass.

Example: Phylum— *Bacteroidota*; Class—*Ktedonobacteria*; Subclass— *Sphaerobacteridae*.

Note. The term 'stem' when used in the ICNP corresponds with that part of the word that does not vary among the forms of the noun in the oblique cases, i.e., cases other than the nominative, and which can be obtained by deleting the ending of the genitive singular.

Names of cyanobacterial taxa above the rank of order that are considered validly published under the *International Code of Nomenclature of Prokaryotes* because they are validly published under the *International Code of Nomenclature for algae, fungi, and plants* (see Rule 30) are formed in accordance with the provisions of the latter code.

Names of Taxa between Subclass and Genus (Order, Suborder, Family, Tribe)

Rule 9

The name of a taxon between subclass and genus is formed by the addition of the appropriate suffix to the stem of the name of the type genus (see Rule 15). These suffixes are presented in Table 1.

Table 1. *Suffixes for Categories between Subclass and Genus*

Rank	Suffix	Example
Order	–*ales*	*Pseudomonadales*
Suborder	–*ineae*	*Pseudomonadineae*
Family	–*aceae*	*Pseudomonadaceae*
Tribe	–*eae*	*Pseudomonadeae*

Names of Genera and Subgenera

Rule 10a

The name of a genus or subgenus is a noun, or an adjective used as a noun, in the singular number in the nominative case, and written with an initial capital letter. The name may be taken from any source and may even be composed in an arbitrary manner. It is treated as a Latin noun.

Examples: Single Greek stem, *Clostridium*; two Greek stems, *Xylophilus*; single Latin stem, *Spirillum*; two Latin stems, *Terribacillus*; hybrid name, Latin-Greek stems, *Fluviibacterium*; Latinized personal name, *Shigella*; arbitrary name, *Cedecea*.

As from January 2001, newly proposed names must not be later homonyms of names with standing in botany or zoology (see Principle 2).

Recommendation 10a

The following Recommendations apply when forming new generic or subgeneric names.

(1) Refrain from naming genera and subgenera after persons unconnected with microbiology or at least with natural science.
(2) Give a feminine form to all personal generic and subgeneric names, whether they commemorate a man or a woman (see Appendix 9).

Rule 10b

Generic and subgeneric names are subject to the same Rules and Recommendations, except that Rule 10c applies only to subgeneric names.

Rule 10c

The name of a subgenus, when included with the name of a species, is placed in parentheses along with the abbreviation "subgen." between the generic name and specific epithet. When included, the citation is inserted before closure of the parentheses.

Example: *Acetobacter* (subgen. *Gluconoacetobacter*) *liquefaciens* or *Acetobacter* (subgen. *Gluconoacetobacter* Yamada and Kondo 1985) *liquefaciens* (Asai 1935) Yamada and Kondo 1985.

Rule 11

Editorial Note. The former Rule 11 has been deleted. This rule only remains here as a placeholder in order to avoid renumbering Rules 12 and above. Rule 11 should not be cited.

Names of Species

Rule 12a

The name of a species is a **binary combination** consisting of the name of the genus followed by a single **specific epithet**.

If a specific epithet is formed from two or more words, then the words are to be joined. If the words were not joined at the time of valid publication, then the epithet is not to be rejected or replaced for this reason but the form is to be corrected by joining the words, which can be done by any author. If an epithet has been hyphenated, the parts must be joined. Such corrections of an epithet do not affect the status and date of valid publication of the name.

Example: *Nocardia otitidis-caviarum* has been corrected to *Nocardia otitidiscaviarum*.

Rule 12b

No specific or subspecific epithets within the same genus may be the same if based on different types (see Rules 13c, 40d and Section 9).

Example: *Bacillus pallidus* Scholz *et al.* 1988 is based on the nomenclatural type, strain H12; the specific epithet *pallidus* must not be used for *Bacillus pallidus* Zhou *et al.* 2008, another bacterium whose name is based on a different type.

Rule 12c

A specific epithet may be taken from any source and may even be composed arbitrarily.

Example: *thetaiotaomicron* in *Bacteroides thetaiotaomicron* derived from a combination of the Greek letters *theta*, *iota* and *omicron*.

Example: *safensis* in *Bacillus safensis*, arbitrarily derived from SAF (the spacecraft-assembly facility at the Jet Propulsion Laboratory, Pasadena, CA, USA).

A specific epithet must be treated in one of the three following ways.

(1) As an adjective in the singular number in the nominative case that must agree in gender with the generic name.

Example: *aureus* in *Staphylococcus aureus*.

(2) As a noun in apposition in the nominative case

Example: *obeum* in *Blautia obeum*.

(3) As a noun in the genitive case.

Example: *coli* in *Escherichia coli*.

Recommendation 12c

Authors should attend to the following Recommendations, and those of Recommendation 6, when forming specific epithets.

(1) Choose a specific epithet that gives some indication of a property or of the source of the species.

(2) Avoid those that express a character common to all, or nearly all, the species of a genus.

(3) Specific epithets should not honour the author or co-authors of the proposed species or subspecies, or any persons not connected with microbiology or at least with natural science.

(4) Avoid in the same genus epithets that are very much alike, especially those that differ only in their last letters (see Rule 56a(4)).

(5) Avoid the use of the genitive and the adjectival forms of the same specific epithet to refer to two different species of the same genus (see Appendix 9).

(6) If an ordinal adjective used for enumeration is chosen, then they may include numbers up to ten.

Example: *primus*, *secundus*.

Names of Subspecies

Rule 13a

The name of a subspecies is a **ternary combination** consisting of the name of a genus followed by a specific epithet, the abbreviation “subsp.” (*subspecies*), and finally the **subspecific epithet**.

Example: *Bacillus subtilis* subsp. *spizizenii* Nakamura *et al.* 1999.

Rule 13b

A subspecific epithet is formed in the same way as a specific epithet. When adjectival in form, it agrees in gender with the generic name.

Rule 13c

No two subspecies within the same species or within the same genus may bear the same subspecific epithet (see also Rules 12b and 40d).

Rule 13d

A subspecies that includes the type of the species must bear the same epithet as the species (see also Rules 40d and 45).

Names of Infrasubspecific Subdivisions

Rule 14a

The designations of the various taxa below the rank of subspecies are not subject to the Rules and Recommendations of this *Code* (for advice on their nomenclature, see Appendix 10).

Rule 14b

A Latin or Latinized infrasubspecific designation may be elevated by a subsequent author to the status of a subspecies or species name, providing that the resulting name is in conformity with the Rules. If so elevated, for purposes of priority, it ranks from its date of elevation and is attributed to the author(s) who elevated it, provided that the author(s) who elevated it observe(s) Rule 27.

Example: *Pseudomonas cannabina* (*ex* Šutič and Dowson 1959) Gardan *et al.* 1999; elevation of *Pseudomonas syringae* pathovar Cannabina of (Šutič and Dowson 1959) Young *et al.* 1978 by Gardan *et al.*

Section 4. Nomenclatural Types and Their Designation

General

Rule 15

A taxon consists of one or more elements. For each named taxon of the various taxonomic categories (listed below), there shall be designated a single **nomenclatural type**. The nomenclatural type, referred to in this *Code* as "**type**", is that element of the taxon with which the name is permanently associated, whether as a correct name or as a synonym. The nomenclatural type is not necessarily the most typical or representative element of the taxon. The types are dealt with in Rules 16–22.

Types of the various taxonomic categories are presented in Table 2.

Table 2. *Taxonomic Categories and their Types*

**Taxonomic category**	**Type**
Subspecies<br>Species	Designated strain; in special cases the place of the type strain may be taken by a description, preserved specimen, or an illustration (see Rule 18a(1))
Subgenus<br>Genus	Designated species
Tribe<br>Family<br>Suborder<br>Order<br>Subclass<br>Class<br>Phylum<br>Kingdom	Genus on whose name the name of the higher taxon is based
Domain	One of the contained genera

Rule 16

The type of a taxon must be designated by the author(s) at the time the name of the taxon is published in the IJSEM (see Rules 15, 18a, b, f, 20a-c, 21a, 22, 27(3)), unless the type of the taxon can be inferred according to Rules 20c, 21a, 21b or 22.

Note 1. Authors who intend to publish a name in the IJSEM with reference to a description or listing of the properties of the taxon that has appeared in an effective publication under Rule 27(2) must also designate the type when publishing that description.

Note 2. If a type has not been designated in the effective publication, then the type must be designated at the time of publication in IJSEM, in accordance with the Rules of this *Code*.

Rule 17

The type determines the application of the name of a taxon if the taxon is subsequently divided or united with another taxon.

Example: Ash *et al.* [4] proposed that the genus *Bacillus* be divided into the genera *Bacillus* and *Paenibacillus*, and the genus that contained the type species *Bacillus subtilis* must be named *Bacillus*.

Type of a Species or Subspecies

Rule 18a

Whenever possible, the type of a species or subspecies is a designated strain.

The type strain is made up of living cultures of an organism, which are descended from a strain designated as the nomenclatural type. The strain should have been maintained in pure culture and should agree closely to its characters with those in the original description. The type strain may be designated in various ways (see Rules 18b, 18c, and 18d).

(1) Until 31 December 2000, wherein a type strain has not so far been maintained in laboratory cultures or for which a type strain does not exist, a description, preserved specimen, or illustration (see also Rule 18f) may be designated as the type.

Example: Non-cultivated, *Oscillospira guilliermondii* Chatton and Perard 1913.

(2) As from 1 January 2001, no further descriptions, preserved (non-viable) specimens, or illustrations may be designated as the type. This does not affect nomenclatural types designated under Rule 18a(1) until 31 December 2000.

Examples: (a) Pure culture, *Thiocystis cadagnonensis* Peduzzi et al. 2011; (b) pure culture, *Spiribacter salinus* León et al. 2014; (c) co-culture with host: *Nanobdella aerobiophila* Kato et al. 2022.

(3) For species (or subspecies) of *Cyanobacteria* described under the provisions of the *International Code of Nomenclature for algae, fungi, and plants* [5] the type designated under that Code is also recognized as the type under the *International Code of Nomenclature of Prokaryotes*. In cases of homonymy, wherein the name of a cyanobacterial taxon was published under both codes, the oldest name has priority.

Example: *Prochlorococcus* Chisholm *et al.* 1992 and not *Prochlorococcus* Chisholm *et al.* 2001.

Rule 18b *Designation by original author(s)*

If the author(s) of the name of a species or subspecies unambiguously designated a type strain in the effective publication, then this strain shall be accepted as the type strain and may be referred to as the **holotype**.

Rule 18c *Designation as neotype*

If a strain on which the original description was based cannot be found, a **neotype** strain may be proposed. A **neotype** strain must be proposed (**proposed neotype**) in the IJSEM, together with citation of the author(s) of the name, a description or reference to a description or listing of the properties of the taxon that has appeared in an effectively published description, and a record of the publicly accessible culture collection(s) where the strain is deposited (see also Note 1 to Rule 24a).

The author(s) must show that a careful search for the strains used in the original description has been made and that none can be found. This is not restricted to the deposits of the strain bearing the culture collection number mentioned in the valid publication, but refers to any culture derived from the original culture of the strain. The author(s) must also

demonstrate that the proposed neotype agrees closely with the description given by the original author(s).

The neotype becomes established (**established neotype**) two years after the date of its publication in the IJSEM, provided that no objection has been referred within the first year of the publication of the neotype to the Judicial Commission for consideration.

Note. The term “**strain**” refers to the culture or subcultures of it, described in the original description.

Example: Roop *et al.* [6] proposed a neotype strain (strain VPI S-17 =ATCC 35980) for *Campylobacter sputorum* (Prévot 1940) Véron and Chatelain 1973 (Approved Lists 1980) because the type strain Forsyth ER33 was no longer extant. No objection has been referred and the neotype strain of *Campylobacter sputorum* is the strain VPI S-17=ATCC 35980.

Rule 18d

A strain suggested as a neotype but not formally proposed in accordance with the requirements of Rule 18c (suggested neotype) may not serve as a neotype until formally proposed and established.

Rule 18e

If an original strain that should constitute the type of a species is discovered subsequent to the formal proposal or establishment of a neotype for that species, the matter shall be referred to the Judicial Commission.

Rule 18f

If a description or illustration constitutes, or a dead preserved specimen has been designated the type of a species (Rule 18a(1)) and, later, a strain of this species is cultivated, then the type strain may be designated by the person who isolated the strain or by a subsequent author. This type strain shall then replace the description, illustration or preserved specimen as the nomenclatural type. The designation of a type strain in this manner must be published in the IJSEM, the authorship and date of priority of publication being determined by the valid publication of the name by the original author(s) (Rule 24b).

Rule 18g *Change in characters of type and neotype strains*

If a type or neotype strain has become unsuitable, owing to changes in its characters or for other reasons, then the matter should be referred to the Judicial Commission, which may decide to take action leading to replacement of the strain.

Rule 19 Reference strains

A **reference strain** is a strain that is neither a type nor a neotype strain but a strain used in comparative studies, e.g., taxonomic or serological, or for chemical assay.

A **reference strain** may, by subsequent action, be made a neotype, but otherwise has no formal status under this *Code*.

Type of a Genus

Rule 20a

The nomenclatural type (see Rule 15) of a genus or subgenus is the type species, i.e., the single species or one of the species included when the name was originally validly published. Only species whose names are validly published and legitimate may serve as types

Rule 20b *Designation by original author(s)*

If the author(s) of the effective publication of a generic or subgeneric name designated a type species, that species shall be accepted as the type species.

Rule 20c *Genus with only one species*

If the genus, when its name is validly published, included only one species, then that species is the type species irrespective of whether it is designated as the type.

Rule 20d

Editorial Note. The former Rule 20d has been deleted. This rule only remains here as a placeholder in order to avoid renumbering Rules 20e and above. Rule 20d should not be cited.

Recommendation 20d

Editorial Note. As the former Recommendation 20d has been deleted and remains here only as a placeholder, Recommendation 20d should not be cited.

Rule 20e

Editorial Note. The former Rule 20e has been deleted. This rule only remains here as a placeholder, in order to avoid renumbering Rules 20f and above. Rule 20e should not be cited.

Rule 20f *Retention of type species upon publication of a new generic name*

The valid publication of a new generic name as a deliberate substitute for an earlier one does not change the type species of the genus.

Example: The deliberate creation of *Xanthomonas* as a substitute for the name *Phytomonas* (an illegitimate name~~n~~, as it was already in use as the name of a protozoan genus) does not change the type species, which was *Phytomonas campestris* and which became *Xanthomonas campestris*.

Type of a Subgenus

Rule 20g

A genus and its type subgenus share the same type species.

Example: *Acetobacter aceti* (Pasteur 1864) Beijerinck 1898 (Approved Lists 1980) is the type species of the genus *Acetobacter* Beijerinck 1898 (Approved Lists 1980) and of its type subgenus, *Acetobacter* (Beijerinck 1898) Yamada and Kondo 1985.

Type of a Taxon from Genus to Order (Tribe, Family, Suborder, and Order)

Rule 21a

The nomenclatural type (see Rule 15) of a taxon above genus, up to and including order, is the included genus with a validly published and legitimate name on which the name of the relevant taxon is based. One taxon of each category must include the type genus. The names of the taxa that include the type genus must be formed by the addition of the appropriate suffix to the stem of the name of the type genus (see Rule 9).

Example: Order, *Pseudomonadales*; suborder, *Pseudomonadineae*; family, *Pseudomonadaceae*; tribe, *Pseudomonadeae*; type genus, *Pseudomonas*.

Rule 21b

If the name of a family was not formed in conformity with Rule 21a but its name has been conserved, then the type genus may be fixed by an Opinion of the Judicial Commission.

Example: The genus *Escherichia* is the type genus of the family *Enterobacteriaceae* (Opinion 15).

Type of a Taxon Higher than Order

Rule 22

The type of a domain, kingdom or phylum is one of the contained genera. If there is only one genus, this becomes the type. If there are two or more genera, the type shall be designated by the author(s) at the time of the proposal of the phylum name, although authors are encouraged to respect priority by considering which genus was described first. The type of a class or subclass is one of the contained genera. If only one genus name is validly published, this becomes the type. If two or more genera have validly published names, the type shall be designated by the author(s) at the time of the proposal of the name.

If the author(s) designated as the nomenclatural type of a taxon of a rank above genus, up to and including phylum, another taxon above the rank of genus but below the rank of that taxon and contained within that taxon, and if the nomenclatural type of this designated nomenclatural type is a genus with a validly published and legitimate name, then this genus automatically becomes the nomenclatural type of that taxon in place of the designated nomenclatural type.

If not designated, the type of a taxon higher than order may be later designated by an Opinion of the Judicial Commission.

Section 5. Priority, Effective and Valid Publication of Names

Rule 23a

Each taxon above and including species, up to and including order, with a given circumscription, position, and rank can bear only one correct name, i.e., the earliest that is in accordance with the Rules of this *Code*.

The name of a species is a binary combination of a generic name and specific epithet (see Rule 12a). In a given **position**, a species can bear only one correct epithet, that is, the earliest that is in accordance with the Rules of this *Code*.

Example: The species *Yersinia philomiragia* bears this name in the genus *Yersinia*. When placed in the genus *Francisella*, it bears the name *Francisella philomiragia*.

Note 1. In the case of a species, Rule 23a must be applied independently to the generic name and the specific epithet. The specific epithet remains the same on transfer of a species from one genus to another, except for necessary changes of the gender of adjectives used as specific epithets, i.e., to comply with Rule 12c(1), unless the specific epithet has been previously used in the name of another species or subspecies in the genus to which the species is transferred (see Rule 41a).

Example: *Marinobacterium georgiense* González et al. 1997 and *Pseudomonas iners* Iizuka and Komagata 1964 (Approved Lists 1980) were proposed as heterotypic synonyms by Satomi et al. (2002), who suggested the use of the name *Marinobacterium georgiense* González et al. 1997. However, the epithet in *Pseudomonas iners* Iizuka and Komagata 1964 (Approved Lists 1980) has priority over the epithet in *Marinobacterium georgiense* González et al. 1997. This necessitates the proposal of the new combination *Marinobacterium iners* (Iizuka and Komagata 1964) Tindall 2020 as the older heterotypic synonym of *Marinobacterium georgiense* González et al. 1997.

Note 2. The name of a subspecies is a ternary combination of a generic name, a specific epithet, and a subspecific epithet (see Rule 13c). In a given position, a subspecies can bear only one correct subspecific epithet, i.e., the earliest that is in accordance with the Rules of this *Code*. In the case of a subspecies, Rule 23a must be applied independently to the specific and subspecific epithets. The subspecific epithet remains the same on transfer of a subspecies from one species to another, except for necessary changes of the gender of adjectives used as specific epithets, i.e., to comply with Rule 12c(1), unless the subspecific epithet has been previously used in the name of another species or subspecies in the genus to which the subspecies is transferred (see Rule 41a).

Note 3. The date from which all priorities were determined under the previous revisions of the Code was 1 May 1753. After 1 January 1980, under Rule 24a, all priorities date from 1 January 1980 (see also Rule 24b).

Note 4. The Judicial Commission may make exceptions to Rule 23a by the addition of names to the list of **conserved names** (*nomina conservanda*) or to the list of **rejected names** (*nomina rejicienda*) (see Appendix 4). The Judicial Commission may correct the Approved Lists (see Rule 24a).

(1) A **conserved name** (*nomen conservandum*) is a name that must be used instead of all earlier **synonyms** and **homonyms**. A rejected name (*nomen rejiciendum*) is a name that must not be used to designate any taxon. Only the Judicial Commission can conserve or reject names (see also Rules 56a and 56b).

(2) **Opinions** on the conservation or rejection of names, issued by the Judicial Commission, are published with other Opinions in the IJSEM. Opinions are numbered serially.

Note 5. Names may be: **validly published**– the name is included in an effective publication and is accompanied by a description of the taxon or a reference to a description and certain other requirements (see Rules 27–32); **legitimate**—validly published and in accordance with the Rules; **illegitimate**—validly published and contrary to the Rules; **correct**– the name that must be adopted for a taxon under the Rules.

Rule 23b

The date of a name or epithet is that of its valid publication. For purposes of priority, only legitimate names and epithets are taken into consideration (see Rules 32b and 54).

Rule 24a

Valid publication of names (or epithets) that are governed by the Rules of this Code dates from the dates of publication of the *Code*.

Priority of publication dates from 1 January 1980. On that date, all names published prior to 1 January 1980 and included in the Approved Lists of Bacterial Names are treated, for nomenclatural purposes, as though they had been validly published for the first time on that date, the existing types being retained (but see Rule 24b).

Priority of publication for names of *Cyanobacteria* validly published under the provisions of the *International Code of Nomenclature for algae, fungi, and plants* is determined by Article 13.1 of that Code.

Note 1. Names of prokaryotes in the various taxonomic ranks published until 31 December 1977 were assessed by the Judicial Commission, with the assistance of taxonomic experts. Lists of names were prepared together with the names of the author(s) who originally proposed the names. These *Approved Lists of Bacterial Names* were approved by the ICSB and published in the IJSB on 1 January 1980. Names validly published between 1 January 1978 and 1 January 1980 were included in the *Approved Lists of Bacterial Names* (see Appendix 2).

No further names will be added to the Approved Lists. Those names validly published prior to 1 January 1980 but not included in the Approved Lists have no further standing in nomenclature. They were not added to the lists of *nomina rejicienda* and are thus available for reuse in the naming of new taxa. The reuse of a particular name cannot be recommended if such reuse is likely to result in confusion due to previous or continuing use of the name as a synonym, a strain designation, or for other reasons.

The *Approved Lists of Bacterial Names* contain for each name a reference to a description that has appeared in an effective publication and the type, whenever possible. In the case of species or subspecies, if a type strain is available, it is listed by its designation and the culture collection(s) from which it may be obtained is indicated. If such a strain is not available, a reference strain or reference material is indicated, if possible. Neotypes may be proposed, in conformity with Rule 18c on such lists. (For citation of names on the Approved Lists, see Rules 33b and 34a.)

Note 2. These Approved Lists may contain more than one name attached to the same type (**homotypic synonyms**) since the names on the list represent names that were accepted in prokaryotic nomenclature and taxonomy at the time of publication of the Approved Lists and represented the views of microbiologists who held different taxonomic opinions.

Note 3. Synonyms may be **homotypic synonyms** (i.e., more than one name has been associated with the same type) or **heterotypic synonyms** (i.e., different names have been associated with different types that, in the opinion of the microbiologist concerned, belong to the same taxon). The synonym first published is known as the **earlier synonym**, and subsequently published synonyms are known as **later synonyms.**

Note 4. **Homotypic synonyms** were previously referred to as objective synonyms. **Heterotypic synonyms** were previously referred to as subjective synonyms. **Earlier synonyms** were previously referred to as senior synonyms. **Later synonyms** were previously referred to as junior synonyms.

Publication of **homotypic synonyms** in the Approved Lists does not affect prokaryotic nomenclature any more than does the valid publication of homotypic synonyms in currently published prokaryotic taxonomic literature.

Examples: **Homotypic synonyms** – *Pseudomonas mallei* (Zopf 1885) Redfearn *et al.* 1966 (Approved Lists 1980) and *Burkholderia mallei* (Zopf 1885) Yabuuchi *et al.* 1993 are based on the same type. **Heterotypic synonyms** – Kelly and Wood [7] regard *Thiobacillus concretivorus* Parker 1945(Approved Lists 1980) as a heterotypic synonym of *Thiobacillus thiooxidans* Waksman and Joffe 1922(Approved Lists 1980). These two species have different types.

Note 5. A name contained in an Approved List may have a basonym (Rule 34a). If so, that basonym is also the basonym of any subsequent new combination or *nomen novum*, even if the basonym is not included in an Approved List and is not validly published.

Example. The basonym of *Janthinobacterium lividum* (Eisenberg 1891) De Ley et al. 1978 (Approved Lists 1980) is “*Bacillus lividus*” Eisenberg 1891.

Rule 24b

When the nomenclatural types of two or more taxa that are considered to be heterotypic synonyms, priority of the names or epithets and consequently which are the correct names or correct epithets are determined as follows (see also Rule 23a and 23b):

(1) If two or more names or epithets based on different nomenclatural types compete for priority (i.e., the names or combinations are considered to be heterotypic synonyms) and if all names or epithets were included on an Approved List, priority shall be determined by the date of the name or epithet given on the Approved List (i.e., before 1 January 1980) unless an earlier name or epithet is illegitimate (see Rule 23b). If two or more names or epithets are of the same date, the author who first unites the taxa has the right to choose one of them, and this choice must be followed.

(2) If two or more names or epithets based on different nomenclatural types compete for priority (i.e., the names or combinations are considered to be heterotypic synonyms) and one or more names or epithets appear on an Approved List while the others were otherwise

validly published after 1 January 1980, then priority is determined by the date of the name(s) or epithet(s), as given on the Approved List (i.e., before 1 January 1980) and the date of valid publication of the other name(s) or epithet(s) in the IJSB/IJSEM after 1 January 1980, unless an earlier name or epithet is illegitimate (see Rule 23b). If two or more names or epithets are of the same date, the author who first unites the taxa has the right to choose one of them, and this choice must be followed.

(3) If two or more names or epithets based on different nomenclatural types that are validly published between 1 January 1980 and 31 December 2020 (and therefore not included on the Approved Lists, 1980, or the Corrigenda, 1984) compete for priority (i.e., the names or combinations are considered to be heterotypic synonyms), priority is determined by the date of the valid publication (or announcement) of the name or epithet in the IJSB/IJSEM unless an earlier name or epithet is illegitimate (see Rule 23b).

(4) If two names or epithets appear in the same volume of the IJSB/IJSEM but in different articles, priority is determined by page number or the order of article publication; a name or epithet appearing on a lower page number or an article published earlier in the same issue is deemed to have priority. If two or more names or epithets that appear in the same article compete for priority (i.e., the names or combinations are considered to be heterotypic synonyms) the author who first unites the taxa has the right to choose one of them, and this choice must be followed. In order to implement Rule 24b (2) and 24b (3) in the fairest manner, as of 1st January 1988 (Validation List no 24 onwards), names submitted for inclusion in the Validation List will be allocated a number that reflects the date of receipt of the validation request in the form that is accepted for publication. Wherein names that were included in other printed or electronic publications as effective publications are validly published by announcement on the same Validation List in IJSEM, priority is established by the number allocated on the list. If two or more names or epithets on the same Validation List compete for priority (i.e., the names or combinations are considered to be heterotypic synonyms) and are attributed the same number (or no number was assigned) the author who first unites the taxa has the right to choose one of them, and this choice must be followed.

(5) If two names published after 1 January 2021 in different articles have the same publication date in the IJSEM, priority shall be determined by the date of acceptance for publication.

(6) If two names effectively published in other journals are validly published by announcement in the same Validation List in IJSEM, priority is established by the sequence number on the list.

Note. In order to implement Rule 24b(2) in the fairest manner, names submitted for inclusion in the Validation List will include a sequence number that reflects the date of receipt of the validation request in the form that is accepted for publication.

Example: Sly *et al.* [8] regard *Streptococcus caprinus* Brooker *et al.* 1996 as a heterotypic synonym of *Streptococcus gallolyticus* Osawa *et al.* 1996. *Streptococcus gallolyticus* (Validation List no. 56, priority number 2) having priority over *Streptococcus caprinus* (Validation List no. 56, priority number 7).

Rule 24c

The Judicial Commission may place on the list of **rejected names** (*nomina rejicienda*) a name previously published in an Approved List.

Rule 25a Effective publication

Effective publication is effected under this *Code* by making generally available, by sale or distribution to the scientific community, printed or electronic material for the purpose of providing a permanent record.

When a name of a new taxon is published in a work written in a language unfamiliar to the majority of workers in prokaryotic microbiology, it is recommended that the author(s) include(s) in the publication a description in English.

Note 1. Electronic publication must follow the tradition of publication of printed matter acceptable to this *Code*. Supplementary files attached to papers and papers published in e-print repositories cannot be considered effective publications.

Note 2. Changes to the metadata of an article by the publisher are not considered to be contrary to the intention of creating a permanent record.

Note 3. When a validly published name of a taxon was effectively published in a work that was subsequently retracted, the name remains validly published as long as the properties of the taxon can be accessed to comply with Rule 27 (2). When the properties of the taxon are no longer accessible, the matter must be referred to the Judicial Commission.

Example: The name *Gemella massiliensis* Mbogning Fonkou *et al*. 2023, effectively published in 2021, remains validly published after the effective publication was retracted in 2024, as long as the protologue can be accessed.

Rule 25b

No other kind of publication than that cited in Rule 25a is accepted as effective, nor are the following:

(1) Communication of new names at a meeting, in minutes of a meeting, or, after 1950, in abstracts of papers presented at meetings.

(2) Placing of names on specimens in collections or in listings or catalogues of collections.

(3) Distribution of microfilm, microcards, or matter reproduced by similar methods.

(4) Reports in ephemeral publications, newsletters, newspapers after 1900, or non-scientific periodicals.

(5) Inclusion of a name of a new taxon of prokaryote in a published patent application or issued patent.

(6) Making available electronic material in advance of publication (e.g., papers in press, or otherwise making unpublished manuscripts available in electronic format).

Rule 26a Date of publication

The publication date of a scientific work is the date of publication of the printed or electronic matter. The date given to the work containing the name or epithet must be regarded as correct, in the absence of proof to the contrary.

Rule 26b

The date of acceptance of an article for publication, if given in a publication, does not indicate the effective date of publication and has no significance in the determination of the priority of publication of names.

Valid and Invalid Publication

Rule 27

A name of a new taxon, or a new combination for an existing taxon (or a *nomen novum*, hereafter omitted for brevity), is not validly published unless the following criteria are met:

(1) The name or new combination must have appeared in an effective publication and the name or new combination must be published in the IJSB/IJSEM. For original articles appearing in the IJSB/IJSEM, this journal serves as the effective publication.

(2) The publication of the name or new combination in the IJSB/IJSEM is accompanied by a description of the taxon or by a reference to a description of the taxon that has appeared in an effective publication (see Rules 16, 25a and 25b and, for genus and species, Rules 29–32).

A formal description (‘**protologue**’) must be included in the publication in the IJSEM or in the effectively published description of the taxon published elsewhere. This description must contain the following elements:

(a). The name or new combination must be clearly stated and indicated as such (e.g., fam. nov., gen. nov., sp. nov., comb. nov., etc.).

(b) The derivation (etymology) of a name (and, if necessary, of a new combination) must be given. As of 1 January 2023, for all new combinations, names considered to be homotypic or heterotypic synonyms, together with their authors and dates of valid publication, are to be listed and the basonym indicated.

(c) The properties of the taxon being described must be given directly after (a) and (b). This may include reference to tables or figures in the same publication, or reference to a previous effective publication.

(d) All information contained in (c) must be accessible.

(3) The type of the taxon must be designated (see Rules 15, 16, 18a, b, f, 20a-c, 21a and 22). In the case of species and subspecies, including new combinations, the type strains must be deposited according to Rule 30 and the accession identifiers stated.

Note 1. Valid publication of the name of a taxon requires publication in the IJSB/IJSEM of the name of the taxon and reference to a description in an effective publication, whether in the

IJSB/IJSEM or in another publication. The date of valid publication is that of publication in the IJSB/IJSEM. The name may be mentioned in a previously published description, but the name is not validly published until its publication in the IJSB/IJSEM.

If the initial proposal of the name or new combination is not published in the IJSB/IJSEM, valid publication (**announcement** in a **Validation List**) of the name in the IJSB/IJSEM is primarily the responsibility of the author(s) of the name or new combination, together with the requirements of Rule 27(2) and (3) above. However, other individuals may also submit a name or new combination for announcement. The same applies to names or new combinations published in the IJSB/IJSEM that did not initially meet all the requirements for valid publication.

At the request of the Judicial Commission, the IJSB/IJSEM provides a Notification List that lists all nomenclatural changes as well as listing changes in taxonomic opinion that have occurred in an issue of the journal. After 1 January 2021, the Notification List will include a sequence number that provides the temporal order of publication of articles in an issue of the journal, in lieu of page number. This list has no formal status in prokaryotic nomenclature except to allow for orthographic and grammatical corrections to be made and to fairly establish priority of competing names with a sequence number in lieu of a page number.

In the case of a name of a new taxon, a type must be designated at the time of valid publication unless it can unambiguously be inferred (see Rule 16). In the case of a new combination for an existing taxon, the type must be stated. As of 1 January 2001, the type of a species or subspecies must be deposited in at least two publicly accessible culture collections in different countries from which subcultures must be available [see Rule 30 (3b)]. The description of the taxon should conform to minimal standards (see Recommendation 30).

Note 2. When a new species or a new combination results in the proposal of a new genus, both the genus name and the new species name or new combination must be validly published. Valid publication of the name of the new species or of the new combination alone does not constitute valid publication of the name of the new genus.

Rule 28a

Authors validly publishing a new name after 1 January 1980 may revive a name published prior to 1 January 1980 (see Rule 24a) but not listed in one of the Approved Lists of Bacterial Names unless the name is a *nomen rejiciendum*. The name may be used whether or not the new taxon is related in any way to the taxon to which the name was originally applied.

Authority for the name must be claimed by the new author. If the author wishes to indicate that the name is a revived name and is used to describe a taxon with the same circumscription, position, and rank as that given by the original author, this may be done by appending the abbreviation “nom. rev.” (**revived name**) to the name (see Rule 33c). The proposal must contain a brief diagnosis, i.e., a statement or list of features that led the author to conclude that the proposed taxon is sufficiently different from other recognized taxa to justify its revival. The data included in the statement may be taken from the earlier description and may include newer data. The description of the taxon and derivation of the name must conform to the requirements of Rule 27(2). The type must be designated [see Rule 27(3)].

Note 1. A new name that was previously published before 1 January 1980 is considered to be already validly published only if the name was included in the *Approved Lists of Bacterial Names*.

Note 2. Since revived names are treated as new names, they require valid publication, and the date of priority of a revived name is that of the publication in the IJSEM (see Rule 27).

Note 3. Searching for publication of names and descriptions included in effective publications prior to 1 January 1980 is no longer required. The *Approved Lists of Bacterial Names* form the foundation of a new prokaryotic nomenclature and taxonomy.

Rule 28b

A name or epithet is not validly published in the following circumstances:

(1) It was not accepted at the time of publication by the author(s) who published it. Names or epithets published with a question mark or other indication of taxonomic doubt, yet accepted by the author(s), are not validly published.

(2) It was merely proposed in anticipation of the future acceptance of the taxon concerned or the acceptance of a particular circumscription, position, or rank for the taxon that is being named or in anticipation of the future discovery of some hypothetical taxon.

Examples: (a) *Clostrinium* Fischer 1895 (Opinion 20: *Clostridium*); (b) *Corynebacterium hemophilum* Svendsen *et al.* 1947. "Its haemophilic properties might be used in coining a name, and the name *Corynebacterium hemophilum* is suggested in case further investigation should justify its rank as a species".

(3) It was mentioned incidentally. **Incidental mention** of a new name means mention by an author who does not clearly state or indicate that they are proposing a new name or combination.

Examples: (a) *Pseudobacterium* Trevisan 1888. (b) Raj [9] stated: "Also, recently another organism tentatively named as *Microcyclus marinus* was isolated from the ocean."

Valid Publication of the Name of a Genus or Subgenus, including a Monotypic Genus

Rule 29

For a generic or subgeneric name to be validly published, it must comply with the following conditions:

(1) It must be published in conformity with Rules 27 and 28b.

(2) The valid publication of a genus or subgenus name must include one or more new names or combinations validly published, according to Rule 30.

(3) A nomenclatural type must be selected at the time of valid publication from one of the species included in the genus or subgenus. In the case of a genus or subgenus containing a single species, that species serves as the type (see Rule 20c).

Instead of a new description of the genus or subgenus, a citation to a description or the properties of the genus or subgenus in a previous effective publication may be given. The same holds if a genus is lowered in rank to a subgenus or a subgenus elevated in rank to a genus.

In the case of a genus containing a single species, a combined generic and specific description may be given. In the case of a combined generic and specific description for a genus that contains a single species (see Rule 20c), the name of the new taxon is to be given (i.e., the genus name and the species epithet), indicating that it is both a novel genus and a novel species, gen. nov., sp. nov., followed by the etymology of the genus name and species epithet, in conformity with Rules 27 (2a) and (2b). The requirements of Rule 27 (2c), combining the information for the genus and species, are to be followed. At the time of valid publication, the nomenclatural type of the name at the rank of genus and the name at the rank of species must be given, in conformity with Rule 16 and 27 (3).

Example: *Propioniferax innocua* (Pitcher and Collins 1992) Yokota *et al.* 1994 or *Lamprocystis roseopersicina* (Kützing 1849) Schroeter 1886 (Approved Lists 1980).

Recommendation 29

A description of a genus or subgenus should mention the points in which the genus or subgenus differs from related genera or subgenera. Wherein possible, the family to which it belongs should be mentioned.

Valid Publication of the Name of a Species

Rule 30

For the name of a species to be validly published, it must conform to the following conditions.

(1) It must be published in conformity with Rules 27 and 28b.

(2) It must be published as a binary combination consisting of a genus name followed by a single species epithet (see Rule 12a).

(3) (a) Until 31 December 2000, before valid publication of the name of a new species, a nomenclatural type must be designated, according to Rule 18a (1). If the species is cultivated, a culture of the type strain should be deposited in at least one publicly accessible culture collection from which subcultures must be available. The designations allotted to the strain by the culture collections should be quoted in the published description.

(b) As of 1 January 2001, the valid publication of the name of a new species, or new combination previously represented by a viable culture, must include the designation of a type strain (see Rule 18a), and a viable culture of that strain must be deposited in at least two culture collections in different countries that are publicly accessible at the time of publication in the IJSEM and are able to provide the strain to the scientific community. At least one designation allotted to the type strain by a culture collection must be cited in effective publications.

The designations allotted to the type strain by the culture collections are to be quoted at the time of valid publication. Evidence must be presented that the cultures are present, viable, and available (see Rule 30 (4)) at the time of publication in the IJSEM. This does not affect nomenclatural types designated until 31 December 2000 under Rule 18a (1) and Rule 30 3(a).

Note. In exceptional cases, such as organisms requiring specialized facilities (e.g., Risk Group/Biological Safety Level 3, high pressure requirements, etc.), exceptions may be made to this Rule. Exceptions will be considered on individual basis by a committee consisting of the Chair of the ICSP, the Chair of the Judicial Commission and the Editor-in-Chief of the IJSEM. Exceptions will be made known at the time of publication.

Example: *Pyrococcus yayanosii* and *Promethearchaeum syntrophicum*.

For historical exemptions made as part of the transition to the new rules, see Judicial Opinion 81.

(4) Deposits to which access is restricted, such as safe deposits, deposits of strains made solely for current patent purposes, and deposits for which access is not possible until a national authority or any other third party grants permission, may not serve as deposits of type strains. Material Transfer Agreements or other contractual agreements may be attached to deposits of type strains only if these agreements do not prohibit the distribution of subcultures of the deposit for, at least, research for taxonomic purposes.

Names of cyanobacterial taxa validly published under the *International Code of Nomenclature for algae, fungi, and plants* and having one of the categories listed in Rule 5b are also considered validly published under the *International Code of Nomenclature of Prokaryotes* (see General Consideration 5). Conservation and rejection of cyanobacterial names under the *International Code of Nomenclature for algae, fungi, and plants* is recognized by the *International Code of Nomenclature of Prokaryotes*, unless it is overruled by the Judicial Commission. Names of cyanobacterial taxa validly published under the *International Code of Nomenclature for algae, fungi, and plants* but having other categories are not considered validly published under *the International Code of Nomenclature of Prokaryotes* but may serve as basonyms.

Recommendation 30

Before publication of the name and description of a new species, the examination and description should conform to the current **minimal standards** (if available) required for the relevant taxon of prokaryote.

Note 1. Lists of proposed **minimal standards** are prepared for prokaryotic taxa by experts for publication in the IJSEM (see Appendix 6). Such standards may include current tests for the establishment of generic identity and for the diagnosis of the species, i.e., an indication of characters that distinguish the species from others.

Note 2. The aim of proposed minimal standards is to provide guidance on the description of taxa for taxonomists seeking such advice. However, these standards are not to be applied in a way that contradicts Principle 1 (4).

Rule 31a

The name of a species or a subspecies is not validly published if the description is demonstrably ambiguous and cannot be critically identified for purposes of the precise application of the name of a taxon.

Examples: (a) '*Methanobacillus omelianskii*' Bryant et al. 1967, whose description included all component species, was treated as a single species and, thus, was not validly published; (b) *Syntrophobacter wolinii* Boone and Bryant 1984 is validly published, because the species description applies to one member of the syntrophic association with a hydrogen-producing organism.

Rule 31b

The name of a **consortium** is not regulated by this Code, and such a name is not validly published.

Example: '*Cylindrogloea bacterifera*' Perfiliev 1914.

Note. A **consortium** is an aggregate or association of two or more organisms.

Valid Publication of the Name of a Subspecies

Rule 32a

For the name of a subspecies to be validly published, the name of its species must be validly published and it must conform to the following conditions, unless it is created automatically under Rule 40d.

(1) It must be published in conformity with Rules 27 and 28b.

(2) It must be published as a **ternary combination** consisting of the generic name followed by a single specific epithet and this, in turn, by a single subspecific epithet, with the abbreviation "subsp." between the two epithets to indicate the rank (see Rule 13a).

Example: *Bacillus subtilis* subsp. *subtilis*.

(3) The author(s) must clearly indicate that a subspecies is being named.

Recommendation 32a

Recommendation 30 applies to the name of a subspecies with replacement of the word "species" by the word "subspecies".

Publication of a Specific or Subspecific Epithet

Rule 32b

A specific (or subspecific) epithet is not rendered illegitimate by publication of a species (or subspecies) name in which the generic name is illegitimate (see also Chapter 3, Section 8, and example for Rule 20f).

Section 6. Citation of Authors and Names

Proposal and Subsequent Citation of the Name of a New Taxon

Rule 33a

The authors must indicate that a name is being proposed for a new taxon and should do so by the addition of the appropriate abbreviation for the category to which the taxon belongs.

Note 1. Appropriate abbreviations are: '**dom. nov.'** for *dominium novum*, **regn. nov.** for *regnum novum*, '**phyl. nov.**' for *phylum novum*, **'class. nov.'** for *classis nova*, '**ord. nov.**' for *ordo novus*, '**gen. nov.**' for *genus novum*, '**sp. nov.**' for *species nova*, '**comb. nov.**' for *combinatio nova*. Similar abbreviations may be formed as required.

Note 2. Although words or abbreviations in Latin are usually printed in italics, such abbreviations as the above are frequently printed in Roman or boldface type when they follow a Latin scientific name, in order to differentiate them from the name and draw attention to the abbreviation.

Rule 33b

The citation of the name of a taxon that has been proposed previously should include both the name of the author(s) who first published the name and the year of publication. If there are more than two authors of the name, the citation includes only the first author followed by "*et al.*" and the year.

Examples: *Actinomyces bovis* Harz 1877 (Approved Lists 1980); *Acetobacterium woodii* Balch *et al*. 1977 (Approved Lists 1980).

Note 1. Correct citation of a name enables the date of publication to be verified, the original description to be found, and the use of the name by different authors for different organisms to be distinguished.

Example: *Mycobacterium terrae* Wayne 1966 (Approved Lists 1980), not "*Mycobacterium terrae*" Tsukamura 1966.

Note 2. Full citation of the publication should include reference to the page number(s) in the main text of the scientific work in which the name was proposed, not to the summary or abstract of that text, even if the proposal of the name is mentioned in that summary or abstract.

Example: *Bacillus subtilis* (Ehrenberg 1835) Cohn 1872, 174 (Approved Lists 1980). The page number "174" is the page in Cohn's publication [10] on which the proposal of the new combination occurs.
Example for a name published in the IJSEM after 1 January 2021: *Escherichia ruysiae* van der Putten *et al*. 2021, 004609, 6. The page number "6" is the page in article number 004609 on which the proposal of the new name occurs.

Note 3.

(1) The citation of a name that is included in an Approved List can include the name of the original author(s) and date of publication, followed by the words “Approved Lists” in parentheses.

Example: *Bacillus cereus* Frankland and Frankland 1887 (Approved Lists 1980); *Bacillus subtilis* (Ehrenberg 1835) Cohn 1872 (Approved Lists 1980).

(2) Alternatively, a name that is included in an Approved List may be cited simply by the addition of the words ‘‘Approved Lists 1980’’, in parentheses.
Examples: *Bacillus cereus* (Approved Lists 1980); *Bacillus subtilis* (Approved Lists 1980).

(3) If indication is given that a name is included in an Approved List without specification of that list, the abbreviation ‘‘**nom. approb.**’’ (*nomen approbatum*) may be appended to the name of the taxon.

Example: *Bacillus subtilis* nom. approb.

Rule 33c

If a name or epithet that was published prior to 1 January 1980 but not included in an Approved List is proposed for a different or for the same taxon, the name or epithet must be attributed to the author(s) of the proposal (Rule 28a), and the citation should be made according to Rules 33a, 33b, 34a and 34b.

Note 1. If a name or epithet is revived for the same taxon, the author(s) may indicate the fact by addition of the abbreviation ‘‘**nom. rev.**’’ (*nomen revictum*) after the correct abbreviation (Rule 33a) for the category concerned.

Example: *Actinobacillus seminis* sp. nov., nom. rev., or *Leptothrix discophora* sp. nov., nom. rev.

Note 2. If an author wishes to indicate the names of the original authors of a revived name, the author should do so by citation of the name of the taxon, followed by the word ‘‘**ex**’’ and the name of the original authors and the year of publication, in parentheses, followed by the abbreviation ‘‘nom. rev.’’

Example: Palleroni and Holmes (1981) proposed to revive *Pseudomonas cepacia* Burkholder 1950. An author who subsequently referred to this revived name should use the citation *Pseudomonas cepacia* (*ex* Burkholder 1950) Palleroni and Holmes 1981. If the name is subsequently revised, its origins should be perpetuated by the inclusion of the original citation in the form *Burkholderia cepacia* (Palleroni and Holmes 1981 *ex* Burkholder 1950) Yabuuchi *et al.* 1993.

Note 3. If an author wishes to indicate that a reused name has been used for a different taxon, indication is made by citation of the name and the author(s) and year of publication followed by the word ‘‘**non**’’ (or ‘‘not’’) and the name and year of the publication of the author(s) who first used the name.

Example: *Achromobacter* Yabuuchi and Yano 1981 *non Achromobacter* Bergey *et al.* 1923.

Rule 33d

If a name is revived under Rule 33c it may be revived in a new combination; that is, the revived species may be transferred to another genus, or the revived subspecies may be transferred to another species, at the time the name is revived. It is not necessary first to revive the name in the original combination.

Example: '*Actinobacterium meyeri*' has been revived by Cato *et al*. [11] as a species of the genus *Actinomyces* as *Actinomyces meyeri* nom. rev., comb. nov. Subsequent authors can cite it as *Actinomyces meyeri* (*ex* Prévot 1938) Cato *et al.* 1984.

Proposal and Subsequent Citation of a New Combination

Rule 34a

When authors transfer a species to another genus (Rule 41), or a subspecies to another species, the author who makes the transfer should indicate the formation of the **new combination** by the addition to the citation of the abbreviation ''**comb. nov.**'' (*combinatio nova*). The term 'new combination' also applies when a subspecies is elevated to the rank of a species and the subspecific epithet is retained (Rule 50a) or a species is lowered to the rank of subspecies and the specific epithet is retained (Rule 50b).

This form of citation should be used when authors retain the original species epithet or subspecies epithet in a new combination; however, if authors are obliged to substitute a new species epithet or subspecies epithet as a result of homonymy, the abbreviation ''**nom. nov.**'' (*nomen novum*) should be used [see Rule 41a(1)]. The original name is referred to as the **basonym**. The basonym itself has no basonym.

Example: *Anaerovibrio glycerini* Schauder and Schink 1996; *Anaerosinus glycerini* (Schauder and Schink 1996) Strömpl *et al.* 1999.

Note 1. If an author transfers a species that has been included in the Approved Lists to another genus, the proposal of the **new combination** should be made by the addition of the abbreviation ''**comb. nov.**'' (*combinatio nova*), followed by the name in parentheses under which it appeared in the Approved Lists.

Example: The species *Pseudomonas saccharophila* Doudoroff 1940 appeared on the Approved Lists and was transferred by Xie and Yokota [12] to the genus *Pelomonas*, then the proposal by Xie and Yokota would be as follows: *Pelomonas saccharophila* (Doudoroff 1940) comb. nov. Basonym: *Pseudomonas saccharophila* (Approved Lists 1980). Another author citing this proposal would then use the citation, *Pelomonas saccharophila* (Doudoroff 1940) Xie and Yokota 2005 comb. nov. (*Pseudomonas saccharophila* Approved Lists 1980).

Rule 34b

The citation of a **new combination** that has been previously proposed should include the name of the original author(s), in parentheses, followed by the name of the author(s) who proposed the new combination and the year of publication of the new combination.

Example: *Microbacterium oxydans* (Chatelain and Second) Schumann *et al.* 1999 or *Microbacterium oxydans* (Chatelain and Second 1966) Schumann *et al.* 1999.

Note 1. The inclusion of the date of the publication of the original author(s) of the name is preferred, although it is sometimes omitted, since the date can be expected to be found in the publication of the author(s) who proposed the new combination.

Example: *Microbacterium oxydans* (Chatelain and Second 1966) Schumann *et al.* 1999 is preferred to *Microbacterium oxydans* (Chatelain and Second) Schumann *et al.* 1999.

Note 2. However, when the authors who intended to form a new combination are obliged to substitute a new specific epithet to avoid homonymy [see Rule 41a(1)], the name of the author of the original specific epithet is omitted.

Example: *Flavobacterium hydatis* Bernardet *et al.* 1996 is correct, **not** *Flavobacterium hydatis* (Strohl and Tait 1978) Bernardet *et al.* 1996 [see Example to Rule 41a(1) for an explanation].

Rule 34c

When a taxon from subspecies to genus is altered in rank but retains its name or epithet, the original author(s) must be cited, in parentheses, followed by the name of the author(s) who effected the alteration and the year of publication.

Example: *Bifidobacterium globosum* (*ex* Scardovi *et al.* 1969) Biavati *et al.* 1982 to *Bifidobacterium pseudolongum* subsp. *globosum* (Biavati *et al.* 1982) Yaeshima *et al.* 1992.

Citation of the Name of a Taxon for which the Circumscription Has Been Emended

Rule 35

If an alteration of the diagnostic characters or of the circumscription of a taxon modifies the nature of the taxon, the author(s) responsible may be indicated by the addition to the author citation of the abbreviation ''**emend.**'' (*emendavit*) followed by the name of the author(s) responsible for the change.

Example: *Rhodopseudomonas* Czurda and Maresch 1937 emend. van Niel 1944 (see Opinion 49; Judicial Commission).

Citation of a Name Conserved so as to Exclude the Type

Rule 36

A name conserved so as to exclude the type is not to be ascribed to the original author(s), but the author(s) whose concept of the name is conserved must be cited as authority.

Example: The original type species of the genus *Aeromonas* was rejected as a *nomen dubium* (Opinion 48; Judicial Commission). The generic name *Aeromonas* is now attributed to Stanier 1943, not to Kluyver and van Niel 1936, and with a new type species, *A. hydrophila*.

Section 7. Changes in Names of Taxa as a Result of Transference, Union, or Change in Rank

Rule 37a

(1) The name of a taxon must be changed if the nomenclatural type of the taxon is excluded.

Example: On transferring the type species of the genus *Micropolyspora*, *Micropolyspora brevicatena*, to the genus *Nocardia*, Goodfellow and Pirouz [13] did not provide a solution for the taxonomic position of *Micropolyspora angiospora*, *Micropolyspora faeni*, *Micropolyspora internatus* and *Micropolyspora rectivirgula*, which they should have removed from the genus *Micropolyspora*.

(2) Retention of a name in a sense that excludes the type can only be effected by conservation and only by the Judicial Commission (see also Rule 23a). At the time of conservation, the new type is established by the Judicial Commission.

Rule 37b

A change in the name of a taxon is not warranted by an alteration of the diagnostic characters or of the circumscription. A change in a name may be required by one of the following.

(1) An Opinion of the Judicial Commission [see Rule 37a(2) above].

(2) Transfer of the taxon (see Rule 41).

(3) Union with another taxon (Rules 42–44 and 47b).

(4) Change of rank (Rules 48, 49, 50a, 50b).

Rule 38

When two or more taxa of the same rank are united, the name of the taxon under which they are united (and, therefore, the type of the taxon) is chosen by the rule of priority of publication.

Division of a Genus into Multiple Genera or Subgenera, and of a Subgenus into Subgenera

Rule 39a

If a genus is divided into two or more genera or subgenera, the generic name must be retained for one of these. If the name has not been retained (in a previous publication), it must be re-established under Rule 39b. (See Rule 49 when a subgenus is raised to genus).

Example: Ash *et al.* [4] proposed that the genus *Bacillus* be divided into the genera *Bacillus* and *Paenibacillus*, and the genus that contained the type species *Bacillus subtilis* must be named *Bacillus*.

Rule 39b

When a particular species has been designated as the type, the generic name must be retained for the genus that includes that species.

Rule 39c

The provisions of Rules 39a and 39b apply when a subgenus is divided into two or more subgenera, the original subgeneric name being retained for that subgenus that contains the type species.

Division of a Species into Multiple Species or Subspecies, and of a Subspecies into Multiple Subspecies

Rule 40a

When a species is divided into two or more species or subspecies, the specific epithet of the original species must be retained for one of the taxa into which the species is divided or, if the epithet has not been retained (in a previous publication), it must be re-established. (See Rule 50a when a subspecies is elevated to a species).

Rule 40b

The specific epithet must be retained for the species or subspecies that includes the type strain. When no type was designated, one must be designated.

Example: If the species *Bacillus subtilis* is divided into subspecies, the subspecies containing the type strain must be named *Bacillus subtilis* subsp. *subtilis*.

Rule 40c

The provisions of Rules 40a and 40b apply when a subspecies is divided into two or more subspecies, the original subspecies name being retained for that subspecies that contains the type strain.

Note. Although the specific and subspecific epithets in the name of a type subspecies are the same, they do not contravene Rule 12b because they are based on the same type.

Rule 40d

The valid publication of the name of a subspecies that does not include the type of the species, automatically creates the subspecies that includes the nomenclatural type of the species and whose name bears specific and subspecific epithets that are identical to the epithet of the name of the species, unless this subspecies is explicitly proposed in the same effective publication.

The author(s) of the species name must be cited as the author(s) of such an automatically created subspecific name.

Example: "*Vibrio subtilis*" Ehrenberg 1835 to *Bacillus subtilis* (Ehrenberg 1835) Cohn 1872 (Approved Lists 1980) comb. nov. to *Bacillus subtilis* subsp. *subtilis* subsp. nov. The correct authorship of the subspecies is *Bacillus subtilis* subsp. *subtilis* (Ehrenberg 1835) Nakamura *et al.* 1999 [Ehrenberg for the epithet and Nakamura for the new subspecies].

The authority of the species name must be cited as the authority in parentheses of the name of a subspecies that bears specific and subspecific epithets that are identical to the epithet of the name of the species.

Note 1. A consequence of the valid publication of a subspecific name that does not include the type of the species is that another subspecies that does include the type of the species and whose name bears the same specific and subspecific epithets as the name of the type must be validly published. Valid publication of the name at the rank of subspecies, which is based on the same type as that of the species and bears the same specific and subspecific epithets, must conform to Rules 28b and 32a.

Note 2. If names at the rank of subspecies that include the nomenclatural type of the species and whose name bears specific and subspecific epithets that are identical to the epithet of the name of the species, were not validly published, as specified under Rule 40d Note 1, they may by action of the Judicial Commission be ruled to have been validly published, as defined in Rule 46 of the 1975 and 1990 revisions of the *International Code of Nomenclature of Bacteria* and their authorships and dates of valid publication fixed accordingly.

Transfer of a Species to Another Genus

Rule 41a

When a species is transferred to another genus without any change of rank, the specific epithet must be retained, except for necessary changes of gender of adjectives used as specific epithets, to comply with Rule 12c(1), or it must be re-established if it has not been retained (in a previous publication), unless (see Rule 23a Note 1):

(1) The resulting binary combination would be a **later homonym.**

Example: Bernardet *et al.* [14] proposed *Flavobacterium hydatis* for *Cytophaga aquatilis* Strohl and Tait 1978 (Approved Lists 1980) on transfer to *Flavobacterium* because the name *Flavobacterium aquatile* already existed in that genus.

(2) There is available an earlier validly published and legitimate specific or subspecific epithet.

Example: *Paenimyroides marinum* (Song *et al*. 2013) Li 2024, rather than *Paenimyroides aquimaris* (Song *et al*. 2013) Zhang *et al*. 2023, must be proposed on transfer of *Myroides aquimaris* García-López *et al.* 2020 to *Paenimyroides* because there is an earlier validly published and legitimate specific epithet *marinum* in its basonym *Flavobacterium marinum* Song *et al.* 2013.

Rule 41b

If the name of a genus is changed, the specific epithets of the species included under the original generic name must be retained for the same species, when they are transferred to the new genus, except for necessary changes of gender of adjectives used as specific epithets, to comply with Rule 12c(1).

Union of Taxa of Equal Rank

Rule 42

In the case of subspecies, species, subgenera, and genera, if two or more of those taxa of the same rank are united, the oldest legitimate name or epithet is retained.

If the names or epithets are of the same date, the author or group of authors who first unites the taxa has the right to choose one of them, and that choice must be followed.

Recommendation 42

Authors who must choose between two generic names of the same date should note the following:

(1) Designate the name that is better known.

(2) Designate the name that was first accompanied by the description of a species.

(3) If both are accompanied by descriptions of species, designate the name that includes the larger number of species.

(4) In cases of equality with respect to these considerations, designate the more appropriate name.

Union of Genera as Subgenera

Rule 43

When several genera are united as subgenera of one genus, the subgenus that includes the type species of the genus under which union takes place must bear the same name as that genus.

Union of Species of Two or More Genera as a Single Genus

Rule 44

If two or more species of different genera are brought together to form a genus and if these species include the type species of one or more genera, the name of the genus is that associated with the type species having the earliest legitimate generic name.

If no type species is placed in the genus, a new generic name must be proposed and a type species designated.

Example: *Brevibacterium* Breed 1953. None of the included species was a type species of the genera from which the species were transferred, so a new name, *Brevibacterium*, was proposed, with *Brevibacterium linens* as the type species.

Union of Species as Subspecies

Rule 45

When several species are united as subspecies under one species, the subspecies that includes the type strain of the species under which they are united must be designated by the same epithet as the species.

Example: *Streptomyces griseus* subsp. *griseus* (see pp. 214 and 224 in Pridham *et al.* [15]).

Rule 46

Editorial Note. The former Rule 46 has been relocated as Rule 40d. This rule remains here only as a placeholder in order to avoid renumbering Rules 47a and above. Rule 46 should not be cited.

Union of Taxa above Species under a Higher Taxon

Rule 47a

Editorial Note. The former Rule 47a has been deleted. This rule remains here only as a placeholder in order to avoid renumbering Rules 47b and above. Rule 47a should not be cited.

Rule 47b

If no type genera were placed in the taxon, a new name based on the selected type must be proposed for the taxon.

Example: *Peptococcaceae* Rogosa 1971 (see p. 235 in Rogosa [16]).

Recommendation 47b

When two or more taxa of the same rank, from tribe through family, are united under a new taxon of higher rank for which there is no previous validly published name, consideration should be given to selecting the earliest legitimate genus name that is the nomenclatural type of one of the lower-ranking taxa to be the nomenclatural type of the higher-ranking taxon that also derives its name from the name of that genus.

Example: Buchanan in the publication by Breed *et al.* (1957) [17] placed the families *Beggiatoaceae* Migula 1894 (Approved Lists 1980) and *Vitreoscillaceae* Pringsheim 1949 (Approved Lists 1980) in the new order *Beggiatoales*, whose type is *Beggiatoa* Trevisan 1842 (Approved Lists 1980), which was validly published before *Vitreoscilla* Pringsheim 1949 (Approved Lists 1980) and was included in the family. In contrast, Breed *et al.* (1957) [17] chose *Pseudomonas* Migula 1894 (Approved Lists 1980) instead of *Spirillum* Ehrenberg 1832 (Approved Lists 1980) and *Nitrobacter* Winogradsky 1892 (Approved Lists 1980) to form the name of a new suborder: *Pseudomonadineae* Breed *et al*. 1957 (Approved Lists 1980).

Change in Rank

Rule 48

When the rank of a taxon between subgenus and order is changed, the stem of the name must be retained and only the suffix altered, unless the resulting name is contrary to a Rule (see Rule 9).

Example: Elevation of the suborder Nannocystineae Reichenbach 2007 ~~tribe *Pseudomonadeae*~~ to the order *Nannocystales* (Reichenbach 2007) Waite et al. 2020~~family *Pseudomonadaceae*~~.

Rule 49

When a genus is lowered in rank to subgenus, the original name must be retained, unless it is contrary to a Rule. This also applies when a subgenus is elevated to a genus.

Rule 50a

If a subspecies is elevated in rank to a species, the subspecific epithet in the name of the subspecies must become the specific epithet of the name of the species unless the resulting combination is illegitimate.

Example: *Campylobacter pylori* subsp. *mustelae* Fox *et al.* 1988 becomes *Campylobacter mustelae* (Fox *et al.* 1988) Fox *et al.* 1989.

Rule 50b

If a species is lowered in rank to a subspecies, the specific epithet in the name of the species must be used as the subspecific epithet of the name of the subspecies unless the resulting combination is illegitimate.

Example: *Bifidobacterium globosum* (*ex* Scardovi *et al.* 1969) Biavati *et al.* 1982 becomes *Bifidobacterium pseudolongum* subsp. *globosum* (Biavati *et al.* 1982) Yaeshima *et al.* 1992.

Section 8. Illegitimacy, Replacement, Rejection, and Conservation of Names and Epithets

Illegitimate Names

Rule 51a

A name contrary to a Rule is illegitimate and may not be used. However, a name of a taxon that is illegitimate when the taxon is in one taxonomic position is not necessarily illegitimate when the taxon is in another taxonomic position.

Example: If the species *Borrelia burgdorferi* Johnson et al. 1984 ≡ *Borreliella burgdorferi* (Johnson et al. 1984) Adeolu and Gupta 2015 is considered to have a position within the genus *Borreliella* Adeolu and Gupta 2015, the name *Borreliella burgdorferi* must be used. If the species is considered to have a position within the genus *Borrelia* Swellengrebel 1907 (Approved Lists 1980), the name *Borrelia burgdorferi* must be used.

Rule 51b

Among the reasons for which a name may be illegitimate are the following:

(1) If the taxon to which the name was applied, as circumscribed by the author(s), included the nomenclatural type of a name that the author(s) ought to have adopted under one or more of the Rules.

Example: If an author circumscribes a genus to include *Bacillus subtilis*, the type species of the genus *Bacillus*, then the circumscribed genus must be named *Bacillus*.

(2) If the author(s) did not adopt for a binary or ternary combination the earliest legitimate generic name, specific epithet, or subspecific epithet available for the taxon with its particular **circumscription**, **position**, and **rank**.

(3) If the specific epithet must be rejected under Rules 52 or 53.

(4) If a new name or combination validly published before 31 December 2000 is a **later homonym** of a name of a taxon of prokaryotes, fungi, algae, protozoa or viruses.

Example: *Phytomonas* Donovan 1909, a genus of flagellates, antedates *Phytomonas* Bergey *et al.* 1923, a genus of prokaryote (Opinion 14; Judicial Commission).

(5) If a new name or combination validly published on or after 1 January 2001 is a later **homonym** of a validly published name of a taxon of prokaryotes or a name or combination validly published or available under the *International Code of Nomenclature for algae, fungi, and plants* or the *International Code of Zoological Nomenclature*. This does not affect validly published names or combinations not treated as later homonyms prior to 1 January 2001.

Illegitimate Epithets

Rule 52

The following are not to be regarded as specific or subspecific epithets:

(1) A word or phrase that is not intended as a specific epithet.

Example: *Bacillus nova species* Matzuschita.

(2) A word that is an ordinal adjective higher than ten used for enumeration.

Example: *undecimus*, *duodecimus* etc.

(3) A number or letter.

Example: *α* in *Bacillus α* von Freudenreich.

Rule 53

An epithet is illegitimate if it duplicates a specific or subspecific epithet previously validly published for a species or subspecies of the same genus and if this species or subspecies is a different bacterium with a name based upon another type.

Example: *Bacillus pallidus* Scholz *et al.* 1988 is based on the nomenclatural type, strain H12; the specific epithet *pallidus* cannot be used for *Bacillus pallidus* Zhou *et al.* 2008, which is a different bacterium with a name based upon another type.

Replacement of Names or Epithets

Rule 54

A name or epithet illegitimate according to Rules 51b, 53 or 56a is replaced by the oldest legitimate name or epithet in a **binary** or **ternary combination** that in the new position will be in accordance with the Rules (but see Rule 56b).

If no legitimate name or epithet exists, one must be proposed. A specific epithet is not rendered illegitimate by publication of a species name in which the generic name is illegitimate (Rule 32b). An author may use such an epithet, provided that there is no obstacle to its employment in the new position or sense; the resultant combination is treated as a new name and is ascribed to the author(s) of the combination. However, the epithet is ascribed to the original author(s).

Rule 55

A validly published name or epithet is not illegitimate, and may not be replaced, merely because of the following:

(1) It is inappropriate.

Example: *Bacteroides melaninogenicus* does not produce melanin (see Schwabacher *et al.* [18]).

(2) It is disagreeable.

Example: *Myxococcus llanfairpwllgwyngyllgogerychwyrndrobwllllantysiliogogogochensis* Chambers *et al.* 2021.

(3) Another name or epithet is preferable.

(4) Another name or epithet is better known.

(5) It no longer describes the organism.

Example: *Haemophilus influenzae* (does not cause influenza).

(6) It has been cited incorrectly; an incorrect citation can be rectified by a later author.

Example: *Proteus morganii* Yale 1939 (see Lessel [19]).

(7) It contains a grammatical or orthographic error. See Section 9 for spelling corrections.

Rejection of Names or Epithets

Rule 56a

Only the Judicial Commission can place names and epithets on the list of **rejected names** (*nomina rejicienda*) (see Rule 23a, Note 4, and Appendix 4). A name or epithet may be placed on this list for various reasons, including the following:

(1) An **ambiguous name** (*nomen ambiguum*), i.e., a name that has been used with different meanings and thus has become a source of error.

Example: *Aerobacter* Beijerinck 1900 (Opinion 46; Judicial Commission).

(2) A **doubtful name** (*nomen dubium*), i.e., a name whose application is uncertain.

Example: *Leuconostoc citrovorum* (Opinion 45; Judicial Commission).

(3) A **name causing confusion** (*nomen confusum*), i.e., a name based upon a mixed culture.

Example: *Malleomyces* Hallier 1870.

(4) A **perplexing name** (*nomen perplexum*), a name whose application is known but causes uncertainty in prokaryotic nomenclature because of a similarity in spelling (see Rule 57c).

Example: *Mycobacterium marianum* (Opinion 53; Judicial Commission).

(5) A **perilous name** (*nomen periculosum*), i.e., a particular kind (see *Note 1*) of name whose application is likely to lead to accidents endangering health or life or of serious economic consequences.

Example: *Yersinia pseudotuberculosis* subsp. *pestis* (Lehmann and Neumann 1896) Bercovier *et al.* 1981 was rejected as a *nomen periculosum* in order to maintain *Yersinia pestis* (Lehmann and Neumann 1896) van Loghem 1944 (Approved Lists 1980) and *Yersinia pseudotuberculosis* (Pfeiffer 1889) Smith and Thal 1965 (Approved Lists 1980) as separate species (Opinion 60; Judicial Commission).

Note 1. This application is restricted to a proposed change in the specific epithet of a species that is widely recognized as contagious, virulent, or highly toxigenic, for example, to that of a subspecies of a species having a different host range or a degree of contagiousness or virulence. If the Judicial Commission recognizes a high order of risk to health, or of serious economic consequences, an Opinion may be issued that the taxon be maintained as a separate species, without prejudice to the recognition or acceptance of its genetic relatedness to another taxon.

Note 2. Rejecting an epithet within a name automatically places that epithet in all of the name's homotypic synonyms on the list of rejected epithets (Opinion 106), except for the synonyms in which that epithet is conserved (Opinion 60).

Conservation of Names or Epithets

Rule 56b

A **conserved name** (*nomen conservandum*) is a name that must be used instead of all earlier synonyms and homonyms. Similarly, a conserved epithet must be used instead of all earlier counterparts.

Note 1. A conserved name (*nomen conservandum*) is conserved against all other names for the taxon, whether these are cited in the corresponding list of rejected names or not, so long as the taxon concerned is not united with another taxon bearing a legitimate name. In the event of union or reunion with another taxon, the earlier of the two competing names is adopted in accordance with Rules 23a and 23b.

Note 2. Only the Judicial Commission can place names and epithets on the list of **conserved names** (*nomina conservanda*) (see also Rule 23a, Note 4, and Appendix 4).

Note 3. Conserving an epithet within a name automatically places that epithet in all of the name's homotypic synonyms on the list of conserved epithets (Opinion 106), except for the synonyms in which that epithet is rejected (Opinion 60).

Section 9. Orthography

Rule 57a

Any name or epithet must be written in conformity with the spelling of the word from which it is derived and in strict accordance with the rules of Latin and Latinization. Exceptions are provided for typographic and orthographic errors in Rule 61 and orthographic variants in Rules 62a and 62b (see also Appendix 9).

Note. Names of cyanobacterial taxa that are considered validly published under the *International Code of Nomenclature of Prokaryotes* because they are validly published under the *International Code of Nomenclature for algae, fungi, and plants* (see Rule 30) are spelled in accordance with the provisions of the latter *Code*.

Rule 57b

In this Code, orthographic variant means a name (or epithet) that differs from another name only in transliteration into Latin of the same word from a language other than Latin or in its grammatical correctness.

Example: *Haemophilus*, *Hemophilus*.

Rule 57c

If two or more names or two or more epithets in the same genus are so similar (although the words are from different sources) as to cause uncertainty, they may be treated as **perplexing names** (*nomina perplexa*) and the matter referred to the Judicial Commission [see Rule 56a(4)].

Note 1. **Orthographic variants** may be corrected by any author, provided this is done in accordance with the Note to Rule 61.

Note 2. **Perplexing names** may be placed on the list of rejected names only by the Judicial Commission, because decisions on the status of names derived from different sources differing in one or more letters affect many well-known names in the nomenclature of prokaryotes.

Example: The name *Mycobacterium marianum* Penso 1953 is placed on the list of *nomina rejicienda* as a *nomen perplexum* because it is a source of confusion for resembling the name *Mycobacterium marinum* Aronson 1926 (Approved Lists 1980).

Rule 58

If doubt exists about different spellings of the same name or epithet, or if two spellings are sufficiently alike so as to be confused, the question should be referred to the Judicial

Commission, which may issue an **Opinion**. If one of the spellings is preferred by the Judicial Commission, that spelling must be used by succeeding authors.

Example: The epithet ''*megaterium*'' (over ''*megatherium*'') in the species name *Bacillus megaterium* de Bary 1884 (Opinion 1; Judicial Commission).

Rule 59

An epithet, even one derived from the name of a person, must not be written with an initial capital letter.

Example: *Shigella flexneri* (named after Flexner).

Rule 60

Intentional Latinizations involving changes in orthography of personal names, particularly those of earlier authors, must be preserved.

Example: Chauveau has been Latinized as Chauvoe, and *Clostridium chauvoei* is derived from Chauvoe.

Typographic and Orthographic Errors

Rule 61

The **original spelling** of a name or epithet must be retained, except typographic or orthographic errors. Original spelling does not refer to the use of an initial capital letter or to diacritic signs.

Example: The original spelling was *Bacillus megaterium*, not *megatherium* (Opinion 1; Judicial Commission).

An unintentional typographical or orthographic error later corrected by the author(s) is to be accepted in its corrected form without affecting the status and date of valid publication of the name. It can also be corrected subsequently with or without mentioning that the spelling is corrected, although the abbreviation ''**corrig.**'' (corrigendum) may be appended to the name to draw attention to the correction. Succeeding authors may be unaware that the original usage was incorrect and use the spelling of the original authors. Other succeeding authors may follow the correction of previous authors or may independently correct the spelling, but in no case is the use of corrig. regarded as obligatory. None of these corrections affects the status and date of valid publication of the name.

Example: *Pasteurella mairi* (sic) Sneath and Stevens 1990. The typographic error was later corrected by Sneath [21] to *Pasteurella mairii*; this may be cited as *Pasteurella mairii* corrig.

Note. The liberty of correcting a name or epithet under Rules 61, 62a, and 62b must be used with reserve, especially if the change affects the first syllable and, above all, the first letter of the name or epithet.

Orthographic Variants by Transliteration

Rule 62a

Words differing only in transliteration into Latin from other languages that do not use the Latin alphabet are to be treated as **orthographic variants** unless they are used as the names of taxa based upon different types, when they are to be treated as **homonyms**.

Example: *Haemophilus* and *Hemophilus*.

Rule 62b

If orthographic variants exist based on the same type, and there is no clear indication that one is correct, authors have the right of choice.

Personal Names

Rule 63

The genitive and adjectival forms of a personal name are treated as different epithets and not as orthographic variants unless they are so similar as to cause confusion. For the Latinization of personal names, see Appendix 9.

Example: The epithets *pasteurii* (genitive noun) and *pasteurianum* (adjective) are treated as different epithets.

Diacritic Signs

Rule 64

Diacritic signs are not used in the nomenclature of prokaryotes. In names or epithets derived from words with such signs, the signs must be suppressed and the letters transcribed in accordance with established customs for their language of origin.

Gender of Names

Rule 65

The gender of generic names is governed by the following:

(1) A Latin or Classical Greek word adopted as a generic name retains the classical gender of its language of origin. Authors are recommended to give the gender of any proposed generic name.

Example: *Sarcina* (Latin feminine noun, a package).

In cases wherein the classical gender varies, the author has the right of choice between the alternatives (but see Opinion 3 of the Judicial Commission for the masculine gender of -*bacter*).

Example: -*incola* the gender may be masculine or feminine.

Doubtful cases should be referred to the Judicial Commission.

(2) Generic or subgeneric names that are modern compounds derived from two or more Latin or Greek words take the gender of the last component of the compound word.

Example: *Solibacillus* (masculine) a rod from soil, from Latin: *solum* (neuter), soil; and *bacillus* (masculine), little staff.

Note. As of 1 January 2023, generic names ending in –*oides* (from Gr. suff. –*eides* derived from Gr. neut. n. *eidos* that which is seen, form, shape, figure) will have the neuter gender, irrespective of the gender of the word or word element that precedes the –*oides* ending, and names ending in –*opsis* (from Gr. fem. n. *opsis* aspect, appearance) must be treated as feminine.

(3) Arbitrarily formed generic names or vernacular names used as generic names take the gender assigned to them by their authors, but must be based on the usage of comparable words in Latin where appropriate. If the original authors failed to indicate the gender, subsequent authors have the right of choice.

Examples: *Desemzia* Stackebrandt *et al.* 1999 was assigned the feminine gender; *Bergeyella* Vandamme *et al.* 1994 was assigned the feminine gender; *Aestuariivivens* Park *et al.* 2015 was given the neuter gender; no gender was assigned yet to *Pontivivens* Park *et al.* 2015; *Marivivens* Park *et al.* 2016 was given as masculine or feminine; a subsequent author may choose.

Section 10. *Candidatus* names

Rule 66

The *Candidatus* status should be used to propose names of prokaryotic taxa for which some information is available (Rules 67-68), but for which the requirements for valid publication of a name under Rule 27(3) are not met.

(1) A *Candidatus* name consists of the word "*Candidatus*", followed by a taxon name formed in accordance with Sections 2 and 3 of this *Code*.

(2) *Candidatus* names are not validly published and cannot be the correct name of a taxon. However, *Candidatus* names are regulated by this *Code* analogously to validly published names. Unless otherwise indicated, terms specific to the regulation of *Candidatus* names are derived from the analogous terms used for the regulation of names in other sections of this *Code* by adding the prefix *pro-*. *Candidatus* names have a nomenclatural pro-status and may have pro-standing in nomenclature (Rules 67-68), the main consequences of which are implemented by Rules 71-73.

(3) *Candidatus* names may be: pro-validly published—the name is not validly published but is included in an effective publication (Rules 25a-25b) and meets certain other requirements (Rules 67-68); pro-legitimate—the name is pro-validly published and would be legitimate (Section 8) if it was validly published; pro-illegitimate—pro-validly published and not pro-legitimate; pro-correct—the name that must be adopted for a taxon under the Rules if the taxon does not have a correct name and it is of interest to assign a name to it (Rule 71).

(4) A pro-validly published and pro-legitimate *Candidatus* name cannot compete with a validly published and legitimate name for priority (Rule 23a), but it can compete with

another pro-validly published and pro-legitimate *Candidatus* name for pro-priority (Rule 71), by analogy with Rule 23a.

(5) If a nomenclatural question arises in connection with a *Candidatus* name, which is not expressly dealt with in Section 10, the solution to be chosen is in accordance with the Rules and has the most obvious analogy to a nomenclatural solution provided for in other Sections of this *Code*. Doubtful cases should be referred to the Judicial Commission (Appendix 8). The replacement of a pro-illegitimate name or epithet is conducted by analogy with Rules 54-55, but see also Rule 72(4)(b).

Note. For the history of the *Candidatus* status see Appendix 11. Section 10 supersedes Appendix 11 in the event of a conflict.

Pro-valid publication of a Candidatus name

Rule 67

A *Candidatus* name for a new taxon, or a new combination or *nomen novum* for an existing *Candidatus* taxon, is not pro-validly published, unless the following criteria are met:

(1) The name meets the requirements for publication in Rule 27(1).

(2) The name meets the requirements for the formation of *Candidatus* names in Rule 66(1) in conjunction with the requirements for taxon descriptions in Rule 27(2).

(3) The name meets the requirements for nomenclatural types in Rule 27(3), except for the following:

(a) In the case of the name of a species or subspecies, the name does not meet the requirements of Rule 30 but meets the requirements of Rule 69.

(b) In the case of the name of a taxon above the rank of species, the name of its nomenclatural type is not validly published, but it is pro-validly published and pro-legitimate.

Note. Possible exceptions to Rule 67 are defined in Rule 68(2), Rule 68(3) and Rule 68(4).

Rule 68

The date of pro-valid publication of a *Candidatus* name is the date of its publication in the IJSEM.

(1) If the original proposal of the new *Candidatus* name or new *Candidatus* combination was not published in the IJSEM, pro-valid publication of the name may be achieved by its announcement in an IJSEM *Candidatus* List, by analogy with the announcement of a name in a Validation List for the purpose of its valid publication (Rule 27 Note 1).

(2) If a *Candidatus* name does not meet the requirements of Rule 67, but has been included in an IJSEM *Candidatus* List that was published before 1 January 2025, the name is pro-validly published.

(a) If the nomenclatural type of such a name was not mentioned in the *Candidatus* List, a nomenclatural type will be provided in an addendum to the respective *Candidatus* List, published separately in the IJSEM by the List Editors. Such an addendum does not affect the date of pro-valid publication. If several possible nomenclatural types in accordance with Rule 67(3) have been provided in the effective publication, one shall be selected.

(b) If a nomenclatural type in accordance with Rule 67(3) cannot be determined for a name that is pro-validly published because of its inclusion in a *Candidatus* List, this shall also be announced in an addendum to a *Candidatus* List, and the name considered pro-illegitimate.

(3) If a name meets the requirements given in Rule 67, with the exception of the use of the word "*Candidatus*" as stipulated by Rule 67(2), and the name is included in an IJSEM *Candidatus* List, then the name is pro-validly published and is considered to be a *Candidatus* name.

(4) When a name that has been considered validly published is found to not meet the requirements for nomenclatural types in Rule 27(3), but is found to meet the requirements of Rule 67(3), the name should be given *Candidatus* status by announcement in an IJSEM *Candidatus* List, stating the reasons for not meeting the requirements of Rule 27(3) and including the information required by Rule 67(3). The presumed date of valid publication of the name shall then become its date of pro-valid publication.

(5) A *Candidatus* name that has previously been pro-validly published in the IJSEM may be included in an IJSEM *Candidatus* List for the sole purpose of its orthographic or grammatical correction (Section 9). Such inclusion shall not affect the date of pro-valid publication.

Note. In order to determine the relative pro-priority of *Candidatus* names that were included in the same *Candidatus* List for the purpose of their pro-valid publication, each included name should be assigned a number reflecting the date of receipt of the request for inclusion in the *Candidatus* List, by analogy with Rule 24b(4). Within a *Candidatus* List which does not contain such numbers, relative pro-priority of each name is determined by the date of its original publication as indicated in the *Candidatus* List. *Candidatus* Lists published prior to 1 January 2025 may indicate more than one original publication per name. If so, the relative pro-priority of a name is determined by the date of the earliest indicated original publication of that name.

Nomenclatural type of a Candidatus name

Rule 69

The nomenclatural type of a pro-validly published *Candidatus* name is regulated by analogy with Section 4, except for the following:

(1) The kind of nomenclatural type of a pro-validly published *Candidatus* name at the rank of species or subspecies is one of the following (in order of decreasing preference for the kind of nomenclatural type):

(a) A culture containing living cells of the species or subspecies from which characters of use in taxonomy can be obtained, but which does not meet the requirements of Rule 30.

(b) A preserved specimen containing cells of the species or subspecies from which characters of use in taxonomy can be obtained.

(c) A genome sequence obtained from the species or subspecies and deposited in one of the databases belonging to the International Nucleotide Sequence Database Collaboration (INSDC).

(d) A gene sequence obtained from the species or subspecies and deposited in one of the databases belonging to the INSDC.

(2) For the purpose of designating a nomenclatural type under Rule 69(1)(a) or Rule 69(1)(b), the culture or preserved specimen must be deposited in an appropriate collection. Evidence of its availability from that collection must be provided at the time of publication in the IJSEM.

(3) For the purpose of designating a nomenclatural type according to Rule 69(1)(c) or according to Rule 69(1)(d), its INSDC nucleotide sequence accession numbers must be provided at the time of publication in the IJSEM, i.e., identifiers that can be used directly to obtain a nucleotide sequence from INSDC, without the need for an intermediate identifier. The INSDC nucleotide sequence accession numbers provided must specifically and completely cover the nucleotide sequence, and all associated sequence data must be available at the time of publication in the IJSEM. All sequence identifiers required to specifically and completely cover the sequence must be cited in the effective publication.

(a) If sequence identifiers other than INSDC nucleotide sequence accession numbers have been cited in the effective publication, INSDC nucleotide sequence accession numbers that can be unambiguously mapped to those identifiers must be provided when requesting the inclusion of the taxon name in an IJSEM *Candidatus* List.

(b) If INSDC nucleotide sequence accession numbers have been cited in the effective publication but not in the protologue (see Rule 27), these INSDC nucleotide sequence accession numbers must be provided when requesting the inclusion of the taxon name in an IJSEM *Candidatus* List.

(4) For the purpose of designating a nomenclatural type under Rule 69(1)(d), the protologue must include a statement indicating that, in the taxonomic opinion of the authors, the gene sequence is sufficiently specific to distinguish the taxon from other taxa of the same rank. If such a statement has not been included in the protologue (see Rule 27), the statement must be provided when requesting the inclusion of the taxon name in an IJSEM *Candidatus* List.
Note. Possible exceptions to Rule 69(2), Rule 69(3) and Rule 69(4) are defined in Rule 68(2). The nomenclatural type should conform to minimal standards, when these are available for the respective kind of nomenclatural type at the time of effective publication.

Rule 70

The nomenclatural type of a pro-validly published *Candidatus* name at the rank of species or subspecies may be replaced by another nomenclatural type, provided that the replacement type meets the requirements of Rule 69. Such a proposed replacement must be published in the IJSEM.

(1) The possible reasons for replacing the nomenclatural type of a *Candidatus* species or subspecies are the following:

(a) The nomenclatural type is no longer available. This includes deposits of living cultures or specimens that are no longer available, and sequence records that have been suppressed by INSDC.

(b) The kind of the proposed replacement type is preferred over the kind of the nomenclatural type as indicated in Rule 69(1).

(c) The proposed replacement type is of the same the kind as the designated nomenclatural type, but the proposed replacement type is of higher quality.

(2) The proposal of a replacement type in the IJSEM must state the reason for the replacement in sufficient detail. The proposal must also provide evidence that the replacement type belongs to the species or subspecies according to the taxonomic view expressed in the effective publication of the name of the species or subspecies.

(3) The proposal of a replacement type is void if it does not meet the requirements set out in this Rule. Doubtful cases should be referred to the Judicial Commission (Appendix 8).

Note. A replacement type must not be proposed if it meets the requirements of Rule 30. If it does, it should be used instead to propose the name of the species or subspecies for valid publication, provided that this can be done in accordance with Section 5 and Rule 72 of this *Code*.

Candidatus names with nomenclatural pro-standing

Rule 71

If a taxon with a given circumscription, position, and rank has no correct name (Rule 23a), the taxon can bear only one pro-correct name, i.e., the pro-validly published and pro-legitimate name with the earliest date of pro-valid publication (Rule 68). Exceptions to pro-priority can be made by conservation or rejection.

(1) A pro-validly published name or epithet cannot be conserved over a validly published name or epithet, but a pro-validly published name or epithet may be conserved by the Judicial Commission over another pro-validly published name or epithet by analogy with Rule 56b.

(2) The Judicial Commission may, by analogy with Rule 56a, place a pro-validly published name or epithet on the list of rejected names or epithets. Such rejection equally applies to validly published names or epithets.

Note. A taxon for which there is neither a validly published and legitimate name, nor a pro-validly published and pro-legitimate name, has neither a correct name nor a pro-correct name.

Rule 72

From 1 January 2025, when proposing a taxon name for the purpose of its valid publication under this *Code* (Rule 27), a pro-validly published and pro-legitimate *Candidatus* name, or epithet thereof, must be reused under certain conditions, depending on the taxonomic view expressed by the authors in the effective publication (Rules 25a25b) containing the proposal. Under all other conditions, a pro-validly published and pro-legitimate *Candidatus* name, or epithet thereof, must not be reused. This Rule is not retroactive.

(1) If a name above the rank of species is proposed for the purpose of its valid publication under this *Code*, and if, in the taxonomic opinion of the authors, the proposed name is a synonym of a pro-validly published and pro-legitimate *Candidatus* name of the same rank, then that *Candidatus* name must be reused, unless to do so would contravene a Rule of this *Code*.

(2) If a name at the rank of species or subspecies is proposed for the purpose of its valid publication under this *Code*, and if, in the taxonomic opinion of the authors, the proposed name is a synonym of a pro-validly published and pro-legitimate *Candidatus* name at the same rank and in the same position, then that *Candidatus* name must be reused, unless to do so would contravene a Rule of this *Code*.

(3) If a name at the rank of species or subspecies is proposed for the purpose of its valid publication under this *Code*, and if, in the taxonomic opinion of the authors, the proposed name is a synonym of a pro-validly published and pro-legitimate *Candidatus* name at the rank of species or subspecies and in a different position, then the final epithet of that *Candidatus* name must be reused, unless to do so would contravene a Rule of this *Code*.

(4) Not to reuse a pro-validly published and pro-legitimate *Candidatus* name above genus rank when proposing a name for valid publication, while considering the two names to be synonymous, is permitted where this is a necessary condition to form a name, according to the Rules of this *Code*.

Example. If the hypothetical genus with the pro-validly published and pro-legitimate name “*Candidatus* Dedyshiibacter” was the nomenclatural type of a family with the pro-validly published and pro-legitimate name, “*Candidatus* Dedyshiibacteraceae”, and if authors intended to validly publish the name of another genus in the same family, e.g., *Dedyshiibacteroides*, as well as the name of the family, but cannot propose the name *Dedyshiibacter* for valid publication, at the time, then they may propose the name *Dedyshiibacteroidaceae* for the family, but not *Dedyshiibacteraceae*. In this case, the authors should immediately list “*Candidatus* Dedyshiibacteraceae” as a synonym of *Dedyshiibacteroidaceae*.

Rule 73

The original authors of a *Candidatus* name or epithet reused under Rule 72, whether or not as a synonym, are cited by giving the name of the taxon, followed in parentheses by the word “*ex*” and the name of the original authors and the year of pro-valid publication (see Rule 14b and Rule 33c Note 2). Wherein other authors are subsequently to be cited in parentheses (Rule 34b), this form of citation is retained by analogy with the perpetuation of “*ex*” in Rule 33c Note 2. Rule 73 is retroactive, but failure to comply with it does not alter the nomenclatural status of a name.

Note. The original *Candidatus* name from which a reused epithet is taken is known as the pro-basonym, not the basonym (Rule 34a).

CHAPTER 4. ADVISORY NOTES

A. Suggestions for Authors and Publishers

An author who describes and names a new taxon must indicate the rank of the taxon concerned and, where possible, the rank and name of the next higher taxon (e.g., the name of the family to which a new genus is allocated or the name of the order in which a new family is placed). The title of the work concerned should indicate that a new name is published even if the name itself is not quoted in the title.

It is recommended to print scientific names by a different type face, e.g., italic, or by some other device to distinguish them from the rest of the text.

The name of a genus should be spelled without abbreviation the first time it is used with a specific epithet in a publication and in the summary of that publication.

Example: *Bacillus subtilis*.

Later use of the name of the species previously cited usually has the name of the genus abbreviated, commonly to the first letter of the generic name.

Example: *B. subtilis*.

If, however, species are listed belonging to two or more genera that have the same initial letter, the generic name should be used in full, or initial two-letter or three-letter abbreviations should be used. Some subcommittees on taxonomy have recommended three-letter abbreviations to be used in such cases.

B. Quotations of Authors and Names

(1) *Multiple authorship* (**et al.**). When the new name of a taxon is published under two authors, both are cited; when there are more than two authors and when there is no definite designation of a single individual as the author of the name, the citation may be made by listing the names of all the authors or by giving the name of the first author, followed by the abbreviation "*et al.*" (*et alii*).

(2) *Publication in the work of another author* (**in**). When a new name or combination by one author is published in a work of another author, the word "*in*" should be used in the literature cited to connect the names of the two authors. The name of the author of the name of the taxon precedes the name of the author in whose work it is contained.

Example: *Halobacterium* Elazari-Volcani 1957 *in* Breed *et al.* Bergey's Manual of Determinative Bacteriology, 7th ed., 1957, The Williams & Wilkins Co, Baltimore.

(3) Use of "**pro synon.**," "**ex**," "**non**," and "**sic**."

a. When citing a name published as a synonym, the words "as synonym" or "**pro synon.**" should be added to the citation. (For types of **synonym**, see Rule 24a.)

Example: *Wautersia eutropha* pro synon. *Cupriavidus necator*.

b. When an author publishes a name from a manuscript of another author, or revives another author's name (Rule 33c, Note 2), whether as a synonym or not, the word "*ex*" should be used to connect the names of the two authors. The name of the author who publishes the name precedes that of the original author.

Example: *Achromobacter xylosoxidans* (*ex* Yabuuchi and Ohyama 1971) Yabuuchi and Yano 1981 nom. rev. A subsequent author citing this revived name would use the citation *Achromobacter xylosoxidans* (*ex* Yabuuchi and Ohyama 1971) Yabuuchi and Yano 1981 or *Achromobacter xylosoxidans* Yabuuchi and Yano 1981.

c. When citing in synonymy a name invalidated by an earlier homonym, the citation should be followed by the name of the author of the earlier homonym preceded by the word "*non*", preferably with the date of publication added.

Example: *Achromobacter* Yabuuchi and Yano 1981 (*non Achromobacter* Bergey *et al.* 1923).

d. If a name or epithet is adopted with alterations from the form as originally published, including the use of a corrected spelling, the original spelling should be cited in any list of synonyms of the corrected name. The original spelling is followed by the term "*sic*" in parentheses to indicate that the original spelling is accurately cited.

Example: *Bacteroides tectum* (sic) Love *et al.* 1986, changed to *Bacterioides tectus* (corrig.) ("*corrigendum*") Love *et al.* 1986.

(4) *Nomen nudum.* In the citation of a **bare name** (*nomen nudum*), the status of the name should be indicated by adding "*nom. nud.*".

Note. A **bare name** (*nomen nudum*) means a name published without a description or a reference to a previously published description.

Example: *Moraxella* Bøvre 1979 (Supplementary information to Opinion 83).

(5) *Nomen conservandum*. A **conserved name** (*nomen conservandum*) shall be indicated by the addition of the abbreviation "*nom. cons.*" to the citation.

Example: *Pseudomonas* Migula 1894 nom. cons. (Opinion 5).

APPENDIX 1. CODES OF NOMENCLATURE

International Code of Nomenclature of Prokaryotes (ICNP)[1]

[1]. Formerly the *International Code of Nomenclature of Bacteria* (1966), and, earlier, the *International Code of Nomenclature of Bacteria and Viruses* (1958) and the *International Bacteriological Code of Nomenclature* (1948). Also known as the *Bacteriological Code*, and, since 2008, as the *Prokaryotic Code*.

This Appendix lists the current versions of Codes other than the ICNP. Details of earlier versions can be found in Appendix 1 of the 2008 and 2022 revisions of the ICNP [1,3].

Early drafts of the International *Bacteriological Code of Nomenclature* were published in 1947 [22] and reprinted in the *Journal of Bacteriology* in 1948 [23] and as a reprint in the *Journal of General Microbiology* in 1949 [24]. The first edition of the code approved by the Judicial Commission was published as an annotated book in 1958 as the *International Code of Nomenclature of Bacteria and Viruses* [25]. The 1966 revision was published as the *International Code of Nomenclature of Bacteria* in article form, in the *International Journal of Systematic Bacteriology*, as an update to Chapters 1–4 [26]. Subsequent editions were published as books in 1975 (1975 Revision) [27] and 1992 (1990 Revision) [28]. The 2008 revision as the *International Code of Nomenclature of Prokaryotes* was published as a supplement to the *International Journal of Systematic and Evolutionary Microbiology* in 2019 [3]. The 2022 revision was published as a special issue of the *International Journal of Systematic and Evolutionary Microbiology* in 2023 [1].

The Nomenclatural Code for Prokaryotes Described from Sequence Data (the SeqCode)

After the ICSP rejected in 2020 a proposal to allow use of DNA sequences as nomenclatural types for taxa of prokaryotes with a validly published name, a new Code of Nomenclature was developed that allows DNA sequences to serve as nomenclatural types of names designated to be validly published: the Nomenclatural Code for Prokaryotes Described from Sequence Data (the SeqCode) [29]. As this is in conflict with a previous, unambiguous ICSP decision, the SeqCode is not recognized by the ICSP and its institutions. As the rules of the ICNP apply to all prokaryotes (General Consideration 5), the SeqCode contravenes the ICNP.

International Code of Nomenclature for algae, fungi, and plants (ICN) [5][2]

Note: a new revision – the Madrid Code – is scheduled to be published in June 2025

[2]. Formerly the *International Code of Botanical Nomenclature* (ICBN) and earlier, the *International Rules of Botanical Nomenclature*. Also known informally as the *Botanical Code*.

International Code of Nomenclature for Cultivated Plants (ICNCP) [30][3]

[3]. Also known informally as the *Cultivated Plant Code*.

International Code of Zoological Nomenclature (ICZN Code) [31][4]

[4]. Also known informally as the *Zoological Code*. ICZN stands for the International Commission on Zoological Nomenclature.

International Code of Virus Classification and Nomenclature [32]

BioCode

In March 1994, a meeting was held in Egham, United Kingdom, to investigate the feasibility of harmonizing the five major Codes of Nomenclature. The project originally had an implementation goal of January 1, 2000, but failed to receive support from the individual codes of nomenclature. A revised draft of the BioCode was published in 2011 [33] and continues to seek support.

International Code of Phytosociological Nomenclature

In 1976, the International Society for Vegetation Science[5] published a formal code of nomenclature for communities of plant species, the *International Code of Phytosociological Nomenclature* (ICPN). The third edition of the code was jointly prepared by the IAVS and the Fédération Internationale de Phytosociologie (FIP) [34].

[5]. Now the International Association for Vegetation Science (IAVS).

The Phylogenetic Code of Biological Nomenclature (PhyloCode)

The idea of the PhyloCode was conceived in 2004. It aims at providing an alternative system for governing the application of names in ‘clades’, biological groups that consist of an ancestor (an organism, population, or species) and all of its descendants. The latest version is Version 6, published in 2020 [35]

APPENDIX 2. APPROVED LISTS OF BACTERIAL NAMES

The *Approved Lists of Bacterial Names* consist of two Lists that were published on 1 January 1980 in the IJSB [36]:

Approved List 1. Names of taxa above the rank of genus, pp. 231–238.

Approved List 2. Names of genera, species, and subspecies, pp. 239–420.

See also the Corrigenda (1984) [42] and the reprint of the Approved Lists (1989) [37].

For information about the history of the Approved Lists, see Sneath, 2005 [38].

APPENDIX 3. PUBLISHED SOURCES FOR NAMES OF PROKARYOTIC, ALGAL, PROTOZOAL, FUNGAL, AND VIRAL TAXA

The following publications are among the major references for names of prokaryotic, algal, protozoal, fungal, and viral taxa.

Following the introduction of the Approved Lists of Bacterial Names in 1980 [36–38], names published prior to 1980 that did not appear on either of the Approved Lists or the Corrigenda to the Approved Lists are not validly published unless subsequently validly published in accordance with the Rules of this Code. Information on many other names published prior to 1980 is found in the *Index Bergeyana* [20, 40].

Prokaryotic names validly published since 1980 are published in the IJSEM as articles, Notification Lists and Validation Lists [41, 42]. The first Validation List was published in Vol. 27, no. 3 of the IJSB in 1977; Notification Lists were first added in Vol. 41, no 3 of the IJSB in 1991.

Updated information-is found online on the website LPSN - List of Prokaryotic names with Standing in Nomenclature (www.bacterio.net; https://lpsn.dsmz.de/ (accessed: 4 October 2024 [TO BE UPDATED FURTHER] and in the following sources:

- for all groups of prokaryotes: *Bergey's Manual of Systematics of Archaea and Bacteria* [43].
- for pathovars and phytopathogenic bacteria: [44].
- for cyanobacteria: [45].
- for algae: [46–51].
- for protozoa: [52–55].
- for fungi: [56–60].
- for viruses: [61, 62].
- general: [63].

APPENDIX 4. CONSERVED AND REJECTED NAMES OF PROKARYOTIC TAXA
(*Nomina taxorum conservanda et rejicienda*)

List 1. *Conserved and rejected family names of prokaryotes (nomina familiarum conservanda et rejicienda)*

List 2. *Conserved and rejected names of genera of prokaryotes (nomiina generum conservanda)*

List 3. *Conserved specific epithets in names of species of prokaryotes (epitheta specifica conservanda)*

List 4. *Rejected names of divisions of prokaryotes (nominia divisionum rejicienda*)

List 5. *Rejected names of classes of prokaryotes (nomina classium rejicienda)*

List 6. *Rejected names of subclasses of prokaryotes* (*nomina subclassium rejicienda*)

List 7. *Rejected names of orders of prokaryotes (nomina ordinum rejicienda)*

List 8. *Rejected names of genera and subgenera of prokaryotes nomina generum et subgenerum rejicienda)*

List 9. *Rejected specific and subspecific epithets in names of species and subspecies of prokaryotes (epitheta specifica et subspecifica rejicienda)*

The citations are (unless otherwise indicated) to the volumes, pages, and dates of the *International Bulletin of Bacteriological Nomenclature and Taxonomy* until vol. 15 (1965). From vol. 16 (1966) through vol. 49 (1999) the citations are for the *International Journal of Systematic Bacteriology* and thereafter of the *International Journal of Systematic and Evolutionary Microbiology*.

List 1. *Conserved and rejected family names of prokaryotes (nomina familiarum conservanda et rejicienda)*

**Conserved name (*nomen conservandum*)**	**Name of type genus of conserved family**	**Rejected name (*nomen rejiciendum*)**	**Opinion no.**	**Citations**
Enterobacteriaceae	*Escherichia* Castellani and Chalmers 1919, p. 841	*Bacteriaceae* (see Opinion 4,4:142 [1954])	15	8:73–74 (1958)<br>32:464–465 (1982)<br>35:272–273 (1985)<br>36:577–578 (1986)

List 2. *Conserved and rejected names of genera of prokaryotes (nomiina generum conservanda)*

**Conserved generic names (*nomina generum conservanda*)**	**Name of type species of conserved genus**	**Opinion no.**	**Citations**
Aeromonas Stanier 1943	*Aeromonas hydrophila* (Chester 1901) Stanier 1943	48	23:473–474 (1973)
Agrobacterium Conn 1942	*Agrobacterium tumefaciens* (Smith and Townsend 1907) Conn 1942	33	20:10 (1970)
Arthrobacter Conn and Dimmick 1947	*Arthrobacter globiformis* (Conn 1928) Conn and Dimmick 1947	24	8:171–172 (1958)
Bacillus Cohn 1872	*Bacillus subtilis* Cohn 1872	A. (1936)	Proc. 2nd Internatl. Congr. Microbiol. London, 1936; *Journal of Bacteriology*, 33:445 (1937); *International Code of Nomenclature of Bacteria and Viruses* (1958), p. 148
Beggiatoa Trevisan 1842	*Beggiatoa alba* (Vaucher 1803) Trevisan 1845, *Oscillatoria alba* Vaucher 1803	13	4:151–156 (1954)
Chlorobacterium Lauterborn 1916	*Chlorobacterium symbioticum* Lauterborn 1916	6	4:143 (1954)

Chromobacterium Bergonzini 1880	*Chromobacterium violaceum* Bergonzini 1880	16	8:151–152 (1958)
Enterobacter Hormaeche and Edwards 1960	*Enterobacter cloacae* (Jordan 1890) Hormaeche and Edwards 1960	28	13:38 (1963)
Escherichia Castellani and Chalmers 1919	*Escherichia coli* (Migula 1895) Castellani and Chalmers 1919 (basonym *Bacillus coli* Migula 1895, hyponym *Bacterium coli commune* Escherich 1885)	15	8:73–74 (1958)
Gallionella Ehrenberg 1838	*Gallionella ferruginea* Ehrenberg 1838	9	4:146–147 (1954)
Klebsiella Trevisan 1885	*Klebsiella pneumoniae* (Schroeter 1886) Trevisan 1887 (*Bacterium pneumoniae-crouposae* Zopf 1885)	13	4:151–156 (1954)
Kurthia Trevisan 1885	*Kurthia zopfii* (Kurth 1883) Trevisan 1885 (*Bacterium zopfii* Kurth 1883)	13	4:151–156 (1954)
Lactobacillus Beijerinck 1901	*Lactobacillus delbrueckii* Beijerinck 1901 (*non Lactobacillus caucasicus* Beijerinck 1901)	38	21:104 (1971)
Leptotrichia Trevisan 1879	*Leptotrichia buccalis* (Robin 1853) Trevisan 1879 (*Leptothrix buccalis* Robin 1853)	13	4:151–156 (1954)
Listeria Pirie 1940	*Listeria monocytogenes* (Murray, Webb, and Swann 1926) Pirie 1940 (*Bacterium monocytogenes* Murray *et al.* 1926)	12	4:150–151 (1954)
Methanococcus (Approved Lists 1980) emend. Mah and Kuhn 1984	*Methanococcus vannielii* Stadtman and Barker 1951 (Approved Lists 1980)	62	36: 91 (1986)
Methanosarcina (Approved Lists 1980) emend. Mah and Kuhn 1984	*Methanosarcina barkeri* (Approved Lists 1980) emend. Mah and Kuhn 1984	63	36:492 (1986)
Moraxella Lwoff 1939	*Moraxella lacunata* (Eyre 1900) Lwoff 1939	41	21:106 (1971)
Mycoplasma Nowak 1929	*Mycoplasma mycoides* (Borrel *et al.* 1910) Freundt 1955	22	8:166–168 (1958)
Neisseria Trevisan 1885	*Neisseria gonorrhoeae* (Zopf 1885) Trevisan 1885 (*Merismopedia gonorrhoeae* Zopf 1885)	13	4:151–156 (1954)
Nitrobacter Winogradsky 1892	*Nitrobacter winogradskyi* Winslow *et al.* 1917	23	8:169–170 (1958)
Nitrosococcus Winogradsky 1892	*Nitrosococcus nitrosus* (Migula 1900) Buchanan 1925	23	8:169–170 (1958)

Nitrosomonas Winogradsky 1892	*Nitrosomonas europaea* Winogradsky 1892	23	8:169–170 (1958)
Nocardia Trevisan 1889	*Nocardia asteroides* (Eppinger 1891) Blanchard 1896 (replacing *Nocardia farcinica* Trevisan 1889)	13<br>58	3:87–100 (1953)<br>3:141–154 (1953)<br>4:151–156 (1954)<br>35:538 (1985)
Pasteurella Trevisan 1887	*Pasteurella multocida* (Lehmann and Neumann 1899) Rosenbusch and Marchant 1939 (replacing *Pasteurella choleraegallinarum* Trevisan 1887)	13<br>58	4:151–156 (1954)<br>35:538 (1985)
Pediococcus Claussen 1903	*Pediococcus damnosus* Claussen 1903	52	26:292 (1976)
Proteus Hauser 1885	*Proteus vulgaris* Hauser 1885	131	XX:XXX (20XX) PENDNG
Pseudomonas Migula 1894	*Pseudomonas aeruginosa* (Schroeter 1872) Migula 1900 (*Bacterium aeruginosum* Schroeter 1872)	5	2:121–122 (1952)
Rhizobium Frank 1889	*Rhizobium leguminosarum* (Frank 1879) Frank 1889 (*Schinzia leguminosarum* Frank 1879)	34	20:11–12 (1970)
Rickettsia da Rocha-Lima 1916	*Rickettsia prowazekii* da Rocha-Lima 1916	19	8:158–159 (1958)
Rhodococcus Zopf 1891	*Rhodococcus rhodochrous* (Zopf 1891) Tsukamura 1974	130	74:006414 (2024)
Rhodopseudomonas Czurda and Maresch emend. van Niel 1944	*Rhodopseudomonas palustris* (Molisch 1907) van Niel 1944 (*Rhodobacillus palustris* Molisch 1907)	49	24:551 (1974)
Selenomonas von Prowazek 1913	*Selenomonas sputigena* (Flügge 1886) Boskamp 1922 (basonym *Spirillum sputigenum* Flügge 1886)	21	8:163–165 (1958)
Staphylococcus Rosenbach 1884	*Staphylococcus aureus* Rosenbach 1884	17	8:153–154 (1958)
Vibrio Pacini 1854	*Vibrio cholerae* Pacini 1854	31	15:185–186 (1965)

List 3. *Conserved specific epithets in names of species of prokaryotes (epitheta specifica conservanda)*

**Conserved specific epithets (*epitheta specifica conservanda*)**	**Name of species in which specific epithet is conserved**	**Opinion no.**	**Citations**
acidilactici	*Pediococcus acidilactici* Lindner 1887	68	46:835 (1996)
agalactiae	*Streptococcus agalactiae* Lehmann and Neumann 1896 (*Streptococcus agalactiae contagiosae* Kitt 1893)	8	4:145–146 (1954)
avium	*Mycobacterium avium* Chester 1901	47	23:472 (1973)
botulinum	*Clostridium botulinum* (van Ermengem 1896) Bergey *et al.* 1923	69	49:339 (1999)
boydii	*Shigella boydii* Ewing 1949	11	4:148–150 (1954)
cholerae	*Vibrio cholerae* Pacini 1854	31	15:185–186 (1965)
enterica	*Salmonella enterica* (*ex* Kauffmann and Edwards 1952) Le Minor and Popoff 1987	80	55:519–520 (2005)
faecalis	*Streptococcus faecalis* Andrewes and Horder 1906	30	13:167 (1963)
fermentum	*Lactobacillus fermentum* Beijerinck 1901	50	24:551–552 (1974)
flexneri	*Shigella flexneri* Castellani and Chalmers 1919 (*Bacillus dysenteriae* Flexner 1900)	11	4:148–150 (1954)
forsythia	*Tannerella forsythia* (Tanner *et al.* 1986) Sakamoto *et al.* 2002	85	58:1974 (2011)
fortuitum	*Mycobacterium fortuitum* da Costa Cruz 1938	51	24:552 (1974)
meningitidis	The meningococcus (*Diplococcus intracellularis meningitides* Weichselbaum 1887)	35	20:13–14 (1970)
pestis	*Yersinia pestis* (Lehmann and Neumann 1899) van Loghem 1944	60	35:540 (1985)
phenylpyruvica	*Moraxella phenylpyruvica* Bøvre and Henriksen 1967	42	21:107 (1971)
prowazekii	*Rickettsia prowazekii* da Rocha-Lima 1916	19	8:158–159 (1958)
ramosa	*Pasteuria ramosa* Metchnikoff 1888 emend. Starr *et al.* 1983	61	36:119 (1986)
rhusiopathiae	*Erysipelothrix rhusiopathiae* (Migula 1900) Buchanan 1918	32	20:9 (1970)
sonnei	*Shigella sonnei* (Levine 1920) Weldin 1927 (*Bacterium sonnei* Levine 1920)	11	4:148–150 (1954)
sphaeroides	*Rhodopseudomonas sphaeroides* van Niel 1944	43	21:108 (1971)

sporogenes	*Clostridium sporogenes* (Mechnikoff 1908) Bergey *et al.* 1923	69	49:339 (1999)
typhi	*Salmonella typhi* (Schroeter 1886) Warren and Scott 1930 (*Bacillus typhi* Schroeter 1886)	18	13:31–33 (1963), see also 8:155–156 (1958)

List 4. *Rejected names of divisions of prokaryotes (nomina divisionum rejicienda)*

**Rejected division names (*nomina divsionum rejicienda*)**	**Opinion no.**	**Citations**
Firmicutes corrig. Gibbons and Murray 1978	129	74:006064 (2024)
Gracilicutes Gibbons and Murray 1978	129	74:006064 (2024)

List 5. *Rejected names of classes of prokaryotes (nomina classium rejicienda)*

**Rejected class names (*nomina classium rejicienda*)**	**Opinion no.**	**Citations**
Acidobacteria Cavalier-Smith 2002	79 (suppl.)	64:3599–3602 (2014)
Alphabacteria Cavalier-Smith 2002	79 (suppl.)	64:3599–3602 (2014)
Anoxyphotobacteria (Gibbons and Murray 1978) Murray 1988	128	73:005796 (2023)
Arabobacteria Cavalier-Smith 2002	79 (suppl.)	64:3599–3602 (2014)
Archaeobacteria Murray 1988	128	73:005796 (2023)
Archaeoglobea Cavalier-Smith 2002	79 (suppl.)	64:3599–3602 (2014)
Arthrobacteria Cavalier-Smith 2002	79 (suppl.)	64:3599–3602 (2014)
Bacteria Haeckel 1894 (Approved Lists 1980)	128	73:005796 (2023)
Chlamydiae Cavalier-Smith 2002	79 (suppl.)	64:3599–3602 (2014)
Chlorobacteria Cavalier-Smith 2002	79 (suppl.)	64:3599–3602 (2014)
Chlorobea Cavalier-Smith 2002	79 (suppl.)	64:3599–3602 (2014)
Chromatibacteria Cavalier-Smith 2002	79 (suppl.)	64:3599–3602 (2014)
Chroobacteria Cavalier-Smith 2002	79 (suppl.)	64:3599–3602 (2014)
Crenarchaeota Cavalier-Smith 2002	79 (suppl.)	64:3599–3602 (2014)

Deltabacteria Cavalier-Smith 2002	79 (suppl.)	64:3599–3602 (2014)
Epsilobacteria Cavalier-Smith 2002	79 (suppl.)	64:3599–3602 (2014)
Ferrobacteria Cavalier-Smith 2002	79 (suppl.)	64:3599–3602 (2014)
Firmibacteria Murray 1988	128	73:005796 (2023)
Flavobacteria Cavalier-Smith 2002	79 (suppl.)	64:3599–3602 (2014)
Gloeobacteria Cavalier-Smith 2002	79 (suppl.)	64:3599–3602 (2014)
Hadobacteria Cavalier-Smith 2002	79 (suppl.)	64:3599–3602 (2014)
Halomebacteria Cavalier-Smith 2002	79 (suppl.)	64:3599–3602 (2014)
Hormogoneae Cavalier-Smith 2002	79 (suppl.)	64:3599–3602 (2014)
Methanothermea Cavalier-Smith 2002	79 (suppl.)	64:3599–3602 (2014)
Microtatobiotes Philip 1956 (Approved Lists 1980)	128	73:005796 (2023)
Oxyphotobacteria (ex Gibbons and Murray 1978) Murray 1988	128	73:005796 (2023)
Photobacteria Gibbons and Murray 1978 (Approved Lists 1980)	128	73:005796 (2023)
Picrophilea Cavalier-Smith 2002	79 (suppl.)	64:3599–3602 (2014)
Planctomycea Cavalier-Smith 2002	79 (suppl.)	64:3599–3602 (2014)
Proteobacteria Stackebrandt *et al*. 1988	128	73:005796 (2023)
Protoarchaea Cavalier-Smith 2002	79 (suppl.)	64:3599–3602 (2014)
Schizomycetes Nägeli 1857 (Approved Lists 1980)	128	73:005796 (2023)
Scotobacteria Gibbons and Murray 1978 (Approved Lists 1980)	128	73:005796 (2023)
Spirochaetes Cavalier-Smith 2002	79 (suppl.)	64:3599–3602 (2014)
Streptomycetes Cavalier-Smith 2002	79 (suppl.)	64:3599–3602 (2014)
Teichobacteria Cavalier-Smith 2002	79 (suppl.)	64:3599–3602 (2014)
Togobacteria Cavalier-Smith 2002	79 (suppl.)	64:3599–3602 (2014)

List 6. *Rejected names of subclasses of prokaryotes* (*nomina subclassium rejicienda*)

**Rejected subclass names (*nomina subclassium rejicienda*)**	**Opinion no.**	**Citations**
Anoxyphotobacteriae Gibbons and Murray 1978 (Approved Lists 1980)	129	74:006064 (2024)

List 7. *Rejected names of orders of prokaryotes (nomina ordinum rejicienda)*

**Rejected order names (*nomina ordinum rejicienda*)**	**Opinion no.**	**Citations**
Acidobacteriales Cavalier-Smith 2002	79 (suppl.)	64:3599–3602 (2014)
Actinoplanales Cavalier-Smith 2002	79 (suppl.)	64:3599–3602 (2014)
Cenarchaeales Cavalier-Smith 2002	79 (suppl.)	64:3599–3602 (2014)
Chroococcales Cavalier-Smith 2002	79 (suppl.)	64:3599–3602 (2014)
Geovibriales Cavalier-Smith 2002	79 (suppl.)	64:3599–3602 (2014)
Gloeobacterales Cavalier-Smith 2002	79 (suppl.)	64:3599–3602 (2014)
Nostocales Cavalier-Smith 2002	79 (suppl.)	64:3599–3602 (2014)
Oscillatoriales Cavalier-Smith 2002	79 (suppl.)	64:3599–3602 (2014)
Picrophilales Cavalier-Smith 2002	79 (suppl.)	64:3599–3602 (2014)
Pleurocapsales Cavalier-Smith 2002	79 (suppl.)	64:3599–3602 (2014)
Stigonematales (ex Geitler 1925) Cavalier-Smith 2002	79 (suppl.)	64:3599–3602 (2014)
Streptomycetales Cavalier-Smith 2002	79 (suppl.)	64:3599–3602 (2014)

List 8. *Rejected names of genera and subgenera of prokaryotes nomina generum et subgenerum rejicienda)*

**Rejected generic or subgeneric names (*nomina generum et subgenerum rejicienda*)**	**Names of type species of rejected genera or subgenera**	**Notes**	**Opinion no.**	**Citations**
Aerobacter Beijerinck 1900	*Aerobacter aerogenes* (Kruse 1896) Beijerinck 1900	*Nomen ambiguum*	46	21:110 (1971)
Astasia Meyer 1897	*Astasia asterospora* Meyer 1897	Later homonym of *Astasia* Ehrenberg 1830 (Protozoa)	14	4:156–158 (1954)
Astasia Pribram 1929	None designated. No species listed.	Later homonym of protozoan generic name *Astasia* Ehrenberg 1830	14	4:156–158 (1954)

Babesia Trevisan 1889	*Babesia xanthopyrethica* (sic) Trevisan 1880 (*Streptococcus xanthopyreticus* Trevisan 1887)	The later homonym *Babesia* Starcovici 1893 is in common use as the name of a protozoan genus. *Nomen confusum*	13	4:151–156 (1954)
Bacteriopsis Trevisan 1885 (subgenus)	*Bacteriopsis rasmussenii* Trevisan 1885 (Leptothrix I Rasmussen 1883)	*Nomen confusum*	13	4:151–156 (1954)
Bacterium Ehrenberg 1828	*Bacterium triloculare* Ehrenberg 1828	*Nomen dubium*	4 (revised)	4:142 (1954) see also 1:145–146 (1951) and 3:141–154 (1953)
Billetia Trevisan 1889	*Billetia laminariae* (Billet 1888) Trevisan 1889 (*Bacterium laminariae* Billet 1888)	*Nomen dubium*	13	4:151–156 (1954)
Castellanella Pacheco and Rodrigues 1930	*Castellanella alcalescens* (Andrewes 1918) Pacheco and Rodrigues 1930 (*Bacillus alkalescens* Andrewes 1918)	Illegitimate later homonym of *Castellanella* Chalmers 1918 (Protozoa)	14	4:156–158 (1954)
Cenomesia Trevisan 1889	*Cenomesia albida* Trevisan 1889	*Nomen dubium*	13	4:151–156 (1954)
Chlorobacterium Guillebeau 1890	*Chlorobacterium lactis* Guillebeau 1890		6	4:143 (1954)
Chromobacterium Bergonzini 1879	None designated		16	8:151–152 (1958)
Cloaca Castellani and Chalmers 1919	*Cloaca cloacae* (Jordan 1890) Castellani and Chalmers 1919		28	13:38 (1963)
Coccomonas Orla-Jensen 1921	None designated. No species included.	Later illegitimate homonym of *Coccomonas* Stein 1878 (Protozoa)	14	4:156–158 (1954)
Cornilia Trevisan 1889	*Cornilia alvei* (Cheshire and Cheyne 1885) Trevisan 1889 (*Bacillus alvei* Cheshire and Cheyne 1885)		13	4:151–156 (1954)
Dicoccia Trevisan 1889	*Dicoccia glossophila* Trevisan 1889		13	4:151–156 (1954)
Eucornilia Trevisan 1889 (subgenus)	*Cornilia* (*Eucornilia*) *alvei* (Cheshire and Cheyne 1885) Trevisan 1889 (*Bacillus alvei* Cheshire and Cheyne 1885)		13	4:151–156 (1954)
Eumantegazzaea	*Mantegazzaea* (*Eumantegazzaea*)	*Nomen dubium*	13	4:151–156 (1954)

Trevisan 1889 (subgenus)	*cienkowskii* Trevisan 1889			
Eupacinia Trevisan 1889 (subgenus)	*Pacinia* (*Eupacinia*) *putrifica* (Flügge 1886) Trevisan 1889 (*Bacillus putrificus coli* Flügge 1886)	*Nomen confusum*	13	4:151–156 (1954)
Euspirillum Trevisan 1889 (subgenus)	*Spirillum* (*Euspirillum*) *undula* (Mueller 1873) Ehrenberg 1830 (*Vibrio undula* Mueller 1773)		13	4:151–156 (1954)
Gaffkya Trevisan 1885	*Gaffkya tetragena* (Gaffky 1881) Trevisan 1885		39	21:104–105 (1971)
Herellea De Bord 1942	*Herellea vaginicola* De Bord 1942		40	21:105–106 (1971)
Leptotrichiella Trevisan 1889 (subgenus)	*Leptotrichia* (*Leptotrichiella*) *amphibola* Trevisan 1889	*Nomen dubium*	13	4:151–156 (1954)
Listerella Pirie 1927	*Listerella hepatolytica* Pirie 1927 (*Bacterium monocytogenes* Murray *et al.* 1926)	Illegitimate later homonym of *Listerella* Jahn 1906 (Myxomycetes)	14	4:156–158 (1954)
Mantegazzaea Trevisan 1879	*Mantegazzaea cienkowskii* Trevisan 1879	*Nomen dubium*	13	4:151–156 (1954)
Methanothrix Huser *et al.* 1983	*Methanothrix soehngenii* Huser *et al.* 1983[1]	*Nomen confusum* (type species)	75	58:1753–1754 (2008)
Mima De Bord 1939, 1942	*Mima polymorpha* De Bord 1939, 1942		40	21:105–106 (1971)
Nitromonas Winogradsky 1890	None designated		23	8:169–170 (1958)
Nitromonas Orla-Jensen 1909	None designated		23	8:169–170 (1958)
Octopsis Trevisan 1885	*Octopsis cholerae-gallinarium* Trevisan 1885 (*Micrococcus cholerae-gallinarum* Zopf 1885)		13	4:151–156 (1954)
Palmula Prévot 1938	*Palmula spermoides* Prévot 1938	Illegitimate later homonym of *Palmula* Lea 1833 (Protozoa)	14	4:156–158 (1954)
Pelczaria Poston 1994	*Pelczaria aurantia* Poston 1994		78	55:515 (2005)

Perroncitoa Trevisan 1889	*Perroncitoa scarlatinosa* (Trevisan 1879) Trevisan 1889 (*Micrococcus scarlatinosus* Trevisan 1879)	*Nomen dubium*	13	4:151–156 (1954)
Pfeifferella Buchanan 1918	*Pfeifferella mallei* (Zopf 1885) Buchanan 1918 (*Bacillus mallei* Zopf 1885)	Illegitimate later homonym of *Pfeifferella* Labbé 1899 (Protozoa)	14	4:156–158 (1954)
Phytomonas Bergey *et al.* 1923	*Phytomonas campestris* (Pammel 1895) Bergey *et al.* 1923 (*Bacillus campestris* Pammel 1895)	Illegitimate later homonym of *Phytomonas* Donovan 1909 (Protozoa)	14	4:156–158 (1954)
Pleurospora Trevisan 1889 (subgenus)	*Cornilia* (*Pleurospora*) *tremula* (Koch 1877) Trevisan 1889 (*Bacillus tremulus* Koch 1877)	*Nomen dubium*	13	4:151–156 (1954)
Polymonas Lieske 1928	*Polymonas tumefaciens* (Smith and Townsend 1907) Lieske 1928 (*Bacterium tumefaciens* Smith and Townsend 1907)		33	20:10 (1970)
Pseudospira Trevisan 1889 (subgenus)	*Pacinia* (*Pseudospira*) *choleraeasiaticae* Trevisan 1889		13	4:151–156 (1954)
Pseudospirillum Trevisan 1889 (subgenus)	*Spirillum* (*Pseudospirillum*) *amphibolum* Trevisan 1889	*Nomen dubium*	13	4:151–156 (1954)
Rhizomonas Orla-Jensen 1909 *Rhizomonas* (van Bruggen *et al.* 1990)	None designated. No species included	Later homonym of *Rhizomonas* Kent 1880 (Protozoa) Reaffirmed by Judicial Commission 1999	14	4:156–158 (1954) 50:2242 (2000)
Rhodosphaera Buchanan 1918	*Rhodosphaera capsulata* (Molisch 1907) Buchanan 1918 (*Rhodococcus capsulatus* Molisch 1907)	Later homonym of *Rhodosphaera* Haeckel 1881 (Protozoa)	14	4:156–158 (1954)

[1]This opinion was reconsidered in 2014 by Opinion 75 Supplementary (**64**:3597–3598) and *Methanothrix* Huser *et al.* 1983 is not considered as a rejected name.

List 9. *Rejected specific and subspecific epithets in names of species and subspecies of prokaryotes (epitheta specifica et subspecifica rejicienda)*

**Rejected specific and subspecific epithets (*epitheta specifica et subspecifica rejicienda*)**	**Name of species in which specific or subspecific epithet is rejected**	**Opinion no.**	**Citations**
anaerobius	*Peptococcus anaerobius* (Hamm 1912) Douglas 1957	56	32:468 (1982)
aquae	*Mycobacterium aquae* Jenkins *et al.* 1972	55	32:467 (1982)
aurantia	*Pelczaria aurantia* Poston 1994	78	55:515 (2005)
caucasicus	*Lactobacillus caucasicus* Beijerinck 1901	38	21:104 (1971)
citrovorum	*Leuconostoc citrovorum* (Hammer 1920) Hucker and Pederson 1931	45	21:109–110 (1971)
denitrificans	*Pseudomonas denitrificans* (Christensen 1903) Bergey *et al.* 1923	54	32:466 (1982)
diversus	*Citrobacter diversus* (Burkey 1928) Werkman and Gillen 1932	67	43:392 (1993)
fosteri	*Thermomicrobium fosteri* Phillips and Perry 1976 (Approved Lists 1980)	107	72:005197(2022)
gallicida	*Pasteurella gallicida* (Burrill 1883) Buchanan 1925	58	35:538 (1985)
hoagii	*Corynebacterium hoagii* (Morse 1912) Eberson 1918 (Approved Lists 1980); *Rhodococcus hoagii* (Morse 1912) Kämpfer *et al.* 2014	106	72:005197(2022)
liquefaciens	*Aerobacter liquefaciens* Beijerinck 1901	48	23:473–474 (1973)
marianum	*Mycobacterium marianum* Penso 1953	53	28:334 (1978)
methanica	*Methanosarcina methanica* (Smit 1930) Kluyver and van Niel 1936 (Approved Lists 1980)	63	36:492 (1986)
pestis	*Yersinia pseudotuberculosis* subsp. *pestis* (van Loghem 1944) Bercovier *et al.* 1981	60	35:540 (1985)
polymorpha	*Mima polymorpha* (De Bord 1939) De Bord 1942	40	21:105–106 (1971)
punctata	*Aeromonas punctata* (Zimmermann 1890) Snieszko 1957 (Approved Lists 1980)	123	72:005708 (2022)
putrificum	*Clostridium putrificum* (Trevisan 1889) Reddish and Rettger 1922	69	49:339 (1999)
scabies	*Streptomyces scabies* (*ex* Thaxter 1891) Lambert and Loria 1989	79	70:1439-1440 (2020)
soehngenii	*Methanothrix soehngenii* Huser *et al.* 1983[2]	75	58:1753–1754 (2008)

thermoautotrophica	*Moorella thermoautotrophica* (Wiegel *et al.* 1992) Collins *et al.* 1994	115	72:005481 (2022)
thermophila	*Methanothrix thermophila* Kamagata *et al.* 1992	75 (suppl.)	64:3597–3598 (2014)
vaginicola	*Herellea vaginicola* De Bord 1942	40	21:105–106 (1971)
variabilis	*Halomonas variabilis* (Fendrich 1989)	93	64:3588–3589 (2014)

[2]This opinion was reconsidered in 2014 by Opinion 75 Supplementary (64:3597–3598). *Methanothrix soehngenii* Huser *et al.* 1983 is not considered as a rejected name.

APPENDIX 5. OPINIONS RELATING TO THE NOMENCLATURE OF PROKARYOTES

**List of Opinions**			
Opinions issued by the International Committee on Bacteriological Nomenclature at the Second International Congress for Microbiology, London, 1936			
**Opinion**	**Title**	**Reference and notes**	**Result**
A	Conservation of the generic name *Bacillus* Cohn 1872, designation of the type species, and of the type strain of the species	*J Bacteriol* 1937;33:445–447; and *International Code of Nomenclature of Bacteria and Viruses* (1958), p. 148	(a) It was agreed that *Bacillus* Cohn 1872 should be designated as a *genus conservandum*.<br>(b) It was agreed that the type species of *Bacillus* should be designated as *Bacillus subtilis* Cohn 1872 *emendavit* Prazmowski 1880.<br>(c) It was agreed that the type (or standard) strain should be the Marburg strain.<br>(d) It was agreed that cultures of the type (or standard) strain of *Bacillus subtilis* together with complete description should be maintained at each of the recognized Type Culture Collections.<br>(e) It was agreed that the genus *Bacillus* should be so defined as to exclude bacterial species that do not produce endospores.<br>(f) It was agreed that the term *Bacillus* should be used as a generic name and that it should be differentiated from the terms “bacillus,” “bacille,” and “Bazillus” used as morphological designations.
B	Generic homonyms in the group *Protista*	*J Bacteriol* 1937;33:445–447; *International Code of Nomenclature of Bacteria and Viruses* (1958), p. 148	(a) It was agreed that generic homonyms are not permitted in the group *Protista*.<br>(b) It was agreed that it is advisable to avoid homonyms amongst *Protista* on the one hand, and a plant or animal on the other.
C	Capitalization of specific epithets derived from names of persons	*J Bacteriol* 1937;33:445–447; *International Code of Nomenclature of Bacteria and Viruses* (1958), p. 148	It was agreed that while specific substantive names derived from names of persons may be written with a capital initial letter, all other specific names must be written with a small initial letter.<br>*Note*. This Opinion is revoked by Rule 59 of this *Code*, and Recommendation 27h of the 1958 and 1966 editions of the *International Code of Nomenclature of Bacteria (and Viruses)* stated: “A specific epithet, even one derived from the name of a person, should not be written with an initial capital letter.”

List of Opinions			
Opinions issued by the Judicial Commission			
**Opinion**	**Title**	**Reference and notes**	**Result**
1	The correct spelling of the specific epithet in the species name *Bacillus megaterium* de Bary 1884	*Int Bull Bacteriol Nomencl Taxon* 1951;1:35–36	The spelling *megaterium* of the specific epithet in *Bacillus megaterium* de Bary is preferred to the spelling *megatherium*.
2	The combining forms (stems) of compound bacterial generic names ending in -*bacterium*, -*bacter*, or -*bactrum* (-*bactron*)	*Int Bull Bacteriol Nomencl Taxon* 1951;1:37–38	The combining form or stem of the last component of names ending in -*bacterium* is -*bacteri*, of those ending in –*bactrum* or *bactron* is -*bactr*, and of those ending in -*bacter* is -*bacter*. Family names derived from such generic names have, respectively, the endings -*bacteriaceae*, -*bactraceae*, and -*bacteraceae*.
3	Gender of bacterial names ending in -*bacter*	*Int Bull Bacteriol Nomencl Taxon* 1951;1 (part 2):36–37, and 1952;1:84–85 in re-issue of volume (1951)	*rejiciendum* (rejected generic name).<br>(2) The bacterial family name *Bacteriaceae* The names of bacterial genera that end in -*bacter* should be regarded as having the masculine gender.
4 (revised)	Rejection of generic name *Bacterium* Ehrenberg	*Int Bull Bacteriol Nomencl Taxon* 1954;4:142) see also 1651;1:145–146 and 1953;3:141–154 Minute 9	(1) The bacterial generic name *Bacterium* Ehrenberg 1828 is recognized as a *nomen generis rejiciendum (rejected generic name)*.<br>(2) The bacterial family name *Bacteriaceae* is recognized as a *nomen familiae rejiciendum* (rejected family name).
5	Conservation of the generic name *Pseudomonas* Migula 1894 and designation of *Pseudomonas aeruginosa* (Schroeter) Migula 1900 as type species	*Int Bull Bacteriol Nomencl Taxon* 1952;2:121–122	(1) The generic name *Pseudomonas* Migula 1894 is conserved and placed in the list of *nomina generum conservanda*.<br>(2) The generic name *Pseudomonas* Migula 1894 is associated with the species designated and described by Migula 1895.<br>(3) The type species of the genus *Pseudomonas* Migula 1894 is *Pseudomonas aeruginosa* (Schroeter) Migula 1900 (*Bacterium aeruginosum* Schroeter 1872, *Bacillus pyocyaneus* Gessard 1882, *Pseudomonas pyocyanea* Migula 1895).
6	Conservation of the generic name *Chlorobacterium*	*Int Bull Bacteriol Nomencl Taxon* 1954;4:143	The bacterial generic name *Chlorobacterium* Lauterborn 1916 is conserved against the earlier homonym *Chlorobacterium* Guillebeau 1890. The generic name *Chlorobacterium* Guillebeau 1890 is placed in the list of *nomina generum*

	Lauterborn 1916 against *Chlorobacterium* Guillebeau 1890		*rejicienda*.
7	Nomenclature of the organism associated with granuloma venereum	*Int Bull Bacteriol Nomencl Taxon* 1954;4:144, synonymy of *Calymmatobacterium granulomatis* Aragão and Vianna 1913	The bacterial species names *Encapsulatus inguinalis* Bergey *et al*. 1923, *Klebsiella granulomatis* Bergey *et al*. 1925, *Donovania granulomatis* Anderson, de Monbreun, and Goodpasture 1944 are later synonyms of *Calymmatobacterium granulomatis* Aragão and Vianna 1913.
8	The correct species name of the streptococcus of bovine mastitis	*Int Bull Bacteriol Nomencl Taxon* 1954;4:145–146**,** conservation of the specific epithet *agalactiae* in the combination *Streptococcus agalactiae* Lehmann and Neumann 1896	The species name *Streptococcus agalactiae* Lehmann and Neumann 1896 is conserved against all synonyms having priority.
9	Conservation of the bacterial generic name *Gallionella*	*Int Bull Bacteriol Nomencl Taxon* 1954;4:146–147, conservation of *Gallionella Ehrenberg* 1838, with type species *Gallionella ferruginea* Ehrenberg	*Gallionella* Ehrenberg is placed in the list of conserved names of bacterial genera (*nomina generum conservanda*) with the type species *Gallionella ferruginea* Ehrenberg.
10	Invalidity of the bacterial generic name *Müllerina* de Petschenko 1910 and of the species name *Müllerina paramecia*	*Int Bull Bacteriol Nomencl Taxon* 1954;4:147–148**,** and status of *Drepanospira* de Petschenko 1911 and *Drepanospira muelleri* de Petschenko 1911	The generic name *Müllerina* de Petschenko 1910 and the species name *Müllerina paramecii* de Petschenko 1910 were not accepted by the author, hence were not validly published and are without standing in nomenclature. The later names *Drepanospira* de Petschenko 1911 and *Drepanospira muelleri* de Petschenko 1911 were validly published and are not later synonyms.
11	Nomenclature of species in the bacterial genus *Shigella*	*Int Bull Bacteriol Nomencl Taxon* 1954;4:148–150**,** validity of publication of the names *Shigella dysenteriae* (Shiga) Castellani and Chalmers 1919, and conservation of the specific epithets *flexneri*, *boydii*, and *sonnei* in, respectively, the species names *Shigella flexneri* Castellani and Chalmers 1919, *Shigella boydii* Ewing 1949, and	(1) *Shigella dysenteriae* (Shiga) Castellani and Chalmers 1919 was validly published and is legitimate as the name of the bacterium described by Shiga (1898).<br>(2) The specific epithet *flexneri* in the species name *Shigella flexneri* Castellani and Chalmers 1919 is designated as a conserved specific epithet (*epitheton specificum conservandum*) for the species first described as *Bacillus dysenteriae* Flexner 1900.<br>(3) The species name *Shigella boydii* Ewing 1949 was validly published and is legitimate. The specific epithet *boydii* in the species name *Shigella boydii* is conserved (*epitheton specificum conservandum*).<br>(4) The species name *Shigella sonnei* (Levine) Weldin 1927 was validly published and is legitimate. The specific epithet sonnei in the species name *Shigella sonnei* is

		Shigella sonnei (Levine) Weldin 1927, and emendation, *Int Bull Bacteriol Nomencl Taxon* 1960;10:85 and 1963;13:31	conserved (*epitheton specificum conservandum*).<br>(5) A type or standard culture is to be designated by the *Enterobacteriaceae* Subcommittee on Bacteriological Nomenclature for each of the four species. Such cultures as far as possible shall be maintained in each of the national Type Culture Collections and in the International Shigella Center, Chamblee, Georgia, U.S.A. (*now in the Centers for Disease Control*, *Atlanta*, *Georgia*).<br>(6) A culture belonging to the species *Shigella dysenteriae*, *Shigella flexneri*, *Shigella boydii*, or *Shigella sonnei* may be completely identified by appending the appropriate serotype number (arabic) to the name.
12	Conservation of *Listeria* Pirie 1940 as a generic name in bacteriology	*Int Bull Bacteriol Nomencl Taxon* 1954;4:150–151, type species *Listeria monocytogenes* (Murray, Webb, and Swann) Pirie 1940	*Listeria* Pirie 1940 (type species *Listeria monocytogenes* (Murray, Webb, and Swann) Pirie 1940) shall be placed in the list of conserved names of bacterial genera (*nomina generum conservanda*).
13	Conservation and rejection of names of genera of bacteria proposed by Trevisan 1842–1890	*Int Bull Bacteriol Nomencl Taxon* 1954;4:151–156, conservation of generic names *Beggiatoa*, *Klebsiella*, *Kurthia*, *Leptotrichia*, *Neisseria*, *Nocardia*, *Pasteurella*; rejection of generic names *Babesia*, *Bacteriopsis*, *Billetia*, *Cenomesia*, *Cornilia*, *Dicoccia*, *Eucornilia*, *Eumantegazzaea*, *Eupacinia*, *Euspirillum*, *Leptotrichiella*, *Mantegazzaea*, *Octopsis*, *Perroncitoa*, *Pleurospora*, *Pseudospira*, *Pseudospirillum*; illegitimate generic names *Bollingera*, *Rasmussenia*, *Schuetzia*, *Winogradskya*; of indeterminate status, *Gaffkya*, *Pacinia*	1. Generic names proposed by Trevisan placed in the list of conserved generic names (*nomina generum conservanda*).<br><br>FOR THE CORRECT FORMATTING OF THE GREEN HIGHLIGHTED PARTS OF THE TABLE – DIVISION OF THIS COLUMN INTO MULTIPLE COLUMNS, SEE THE PUBLISHED VERSION OF THE 2022 REVISION<br><br>Names of genera and subgenera Type species<br><br>*Beggiatoa* Trevisan 1842 (p. 56)<br>*Beggiatoa alba* (Vaucher) Trevisan 1845 (*Oscillatori alba* Vaucher 1803)<br><br>*Klebsiella* Trevisan 1885 (p. 105)<br>*Klebsiella pneumoniae* (Schroeter) Trevisan 1887 (*Bacterium pneumoniae crouposae* Zopf 1885)<br><br>*Kurthia* Trevisan 1885 (p. 92)<br>*Kurthia zopfii* (Kurth) Trevisan 1885 (*Bacterium zopfii* Kurth 1883)

			Leptotrichia Trevisan 1879 (p. 138)<br>*Leptotrichia buccalis* (Robin) Trevisan 1879 (*Leptothrix buccalis* Robin 1853)<br><br>*Neisseria* Trevisan 1885 (p. 105)<br>*Neisseria gonorrhoeae* Trevisan 1885<br><br>This generic name was omitted in error in the published Opinion and authority is *Int Bull Bacteriol Nomencl Taxon* 1953;3:141–154 (1953, Minute 7, File 56) and *Int Bull Bacteriol Nomencl Taxon* 1953;3:87–100.<br><br>*Pasteurella* Trevisan 1887 (p. 94)<br>*Pasteurella choleraegallinarum* Trevisan 1887 (but see Opinion 58)<br><br>2. Generic names proposed by Trevisan placed in the list of rejected generic names (*nomina generum rejicienda*).<br><br>Names of genera and subgenera Type species<br><br>*Babesia* Trevisan 1889 (p. 29)<br>*Babesia xanthopyrethica* (sic) Trevisan 1889 (*Streptococcus xanthopyreticus* Trevisan 1887)<br><br>*Bacteriopsis* Trevisan 1885 (p. 103)<br>*Bacteriopsis rasmussenii* Trevisan 1885 (Leptothrix I Rasmussen 1883)<br><br>*Billetia* Trevisan 1889 (p. 11)<br>*Billetia laminariae* (Billet) Trevisan 1889 (*Bacterium laminariae* Billet 1888)<br><br>*Cenomesia* Trevisan 1889 (p. 1039)<br>*Cenomesia albida* Trevisan 1889<br><br>*Cornilia* Trevisan 1889 (p. 21)<br>*Cornilia alvei* (Flügge) Trevisan 1889 (*Bacillus alvei* Flügge 1886)

			Dicoccia Trevisan 1889 (p. 26)<br>*Dicoccia glossophila* Trevisan 1889<br><br>*Eucornilia* Trevisan 1889 (p. 21) (Subgenus)<br>*Cornilia* (*Eucornilia*) *alvei* Trevisan 1889 (*Bacillus alvei* Cheshire and Cheyne 1885)<br><br>*Eumantegazzaea* Trevisan 1889 (p. 942) (Subgenus)<br>*Mantegazzaea* (*Eumantegazzaea*) I *cienkowskii* Trevisan 1879<br><br>*Eupacinia* Trevisan 1889 (p. 23) (Subgenus)<br>*Pacinia* (*Eupacinia*) *putrifica* Trevisan 1889 (*Bacillus putrificus coli* Flügge 1886)<br><br>*Euspirillum* Trevisan 1889 (p. 24) Subgenus)<br>*Spirillum* (*Euspirillum*) *undula* (Mueller) Ehrenberg 1830 (*Vibrio undula* Mueller 1773)<br><br>*Leptotrichiella* Trevisan 1889 (p. 935) (Subgenus)<br>*Leptotrichia* (*Leptotrichiella*) *amphibola* Trevisan 1889<br><br>*Mantegazzaea* Trevisan 1879 (p. 137)<br>*Mantagazzaea cienkowskii* Trevisan 1879<br><br>*Octopsis* Trevisan 1885 (p. 102)<br>*Octopsis choleraegallinarum* Trevisan 1885 (*Micrococcus cholerae-gallinarum* Zopf 1885)<br><br>*Perroncitoa* Trevisan 1889 (p. 29)<br>*Perroncitoa scarlatinosa* (Trevisan) Trevisan 1889 (*Micrococcus scarlatinosus* Trevisan 1879)<br><br>*Pleurospora* Trevisan 1889 (p. 22) (Subgenus)<br>*Cornilia* (*Pleurospora*) *tremula* (Koch) Trevisan 1889 (*Bacillus tremulus* Koch 1877)

			Pseudospira Trevisan 1889 (p. 23) (Subgenus)<br>*Pacinia* (*Pseudospira*) *cholerae-asiaticae* Trevisan 1885 (*Vibrio cholera* Pacini 1854)<br><br>*Pseudospirillum* Trevisan 1889 (p. 25) (Subgenus)<br>*Spirillum* (*Pseudospirillum*) *amphibolum* Trevisan 1889<br><br>3. Trevisan's generic names which, as later homonyms or synonyms, are regarded as illegitimate.<br><br>Names of genera and subgenera Type species<br><br>*Bollingera* Trevisan 1889 (p. 26)<br>*Bollingera equi* (Rivolta) Trevisan (1889) (*Zoogloea pulmonis equi* Bollinger 1870)<br><br>*Rasmussenia* Trevisan 1889 (p. 930)<br>*Rasmussenia buccalis* (Robin) Trevisan 1889 (*Leptothrix buccalis* Robin 1853)<br><br>*Schuetzia* Trevisan 1889 (p. 29)<br>*Schuetzia poelsii* Trevisan 1889 (*Streptococcus equi* Sand and Jensen 1888)<br><br>*Winogradskya* Trevisan 1889 (p. 12)<br>*Winogradskya ramigera* (Itzigsohn) Trevisan 1889 (*Zoogloea ramigera* Itzigsohn 1867)<br><br>4. Trevisan's generic names whose status is indeterminate.<br><br>Names of genera and subgenera Type species<br><br>*Gaffkya* Trevisan 1885 (p. 105); but see Opinion 39<br>*Gaffkya tetragena* (Gaffky) Trevisan 1885 (*Micrococcus tetragenus* Gaffky 1883)<br><br>*Pacinia* Trevisan 1885 (p. 83); but see Opinion 31

			Pacinia choleraeasiaticae Trevisan 1885
14	Names of bacterial genera to be rejected as later synonyms of names of genera of protozoa	*Int Bull Bacteriol Nomencl Taxon* 1954;4:156–158, rejection of *Astasia* Meyer 1897, *Astasia* Pribram 1929, *Castellanella* Pacheco and Rodrigues 1930, Charon Holmes 1948, *Coccomonas* Orla-Jensen 1921, *Listerella* Pirie, 1927, *Palmula* Prévot 1938, *Pfeifferella* Buchanan 1918, *Phytomonas* Bergey *et al.* 1923, *Rhizomonas* Orla-Jensen 1909, *Rhodosphaera* Buchanan 1918	The following names proposed for bacterial genera are found to be later homonyms of names applied to genera of protozoa. Rule 24 of the *International Code of Nomenclature of Bacteria and Viruses* (new Rule 51b) states that such later homonyms are illegitimate in bacteriology. These names are placed in the list of names of bacterial genera to be rejected (*nomina generum bacteriorum rejicienda*).<br><br>FOR THE CORRECT FORMATTING OF THE GREEN HIGHLIGHTED PARTS OF THE TABLE – DIVISION OF THIS COLUMN INTO MULTIPLE COLUMNS, SEE THE PUBLISHED VERSION OF THE 2022 REVISION<br><br>Rejected names of bacterial genera<br>Names of protozoan genera having priority<br><br>*Astasia* Meyer 1897<br>*Astasia* Ehrenberg 1830<br><br>*Astasia* Pribram 1929<br><br>*Castellanella* Pacheco and Rodrigues 1930<br>*Castellanella* Chalmers 1918<br><br>*Charon* Holmes 1948 (a genus of viruses)<br>*Charon* Karsch 1879<br><br>*Coccomonas* Orla-Jensen 1921<br>*Coccomonas* Stein 1878<br><br>*Listerella* Pirie 1927<br>*Listerella* Jahn 1906<br><br>*Palmula* Prévot

			Palmula Lea 1833<br><br>*Pfeifferella* Buchanan 1918<br>*Pfeifferella* Labbé 1899<br><br>*Phytomonas* Bergey *et al.* 1923<br>*Phytomonas* Donovan 1909<br><br>*Rhizomonas* Orla-Jensen 1909<br>*Rhizomonas* Kent 1880<br><br>*Rhodosphaera* Buchanan 1918<br>*Rhodosphaera* Haeckel 1881
15	Conservation of the family name *Enterobacteriaceae*, of the name of the type genus, and designation of the type species	*Int Bull Bacteriol Nomencl Taxon* 1958;8:73–74, with type genus *Escherichia* Castellani and Chalmers 1919 as conserved generic name and type species *Escherichia coli* (Migula) Castellani and Chalmers 1919	(1) The family name *Enterobacteriaceae* Rahn 1937 (p. 280) is placed in the list of family names (*nomina conservanda familiarum*).<br>(2) The genus *Escherichia* Castellani and Chalmers 1919 (p. 941) is designated as the type genus of the family *Enterobacteriaceae* Rahn 1937.<br>(3) The generic name *Escherichia* Castellani and Chalmers 1919 (p. 941) is placed in the list of conserved generic names (*nomina generum conservanda*).<br>(4) The type species of the genus *Escherichia* Castellani and Chalmers 1919 [p. 941 is *Escherichia coli* (Migula) Castellani and Chalmers 1919 p. 941], basonym *Bacillus coli* Migula 1895 (p. 27); hyponym *Bacterium coli commune* Escherich 1885 (p. 518).
16	Conservation of the generic name *Chromobacterium* Bergonzini 1880 and designation of the type species and the neotype culture of the type species	*Int Bull Bacteriol Nomencl Taxon* 1958;8:151–152	(1) The generic name *Chromobacterium* Bergonzini 1879 is rejected and placed in the list of *nomina generum rejicienda*.<br>(2) The generic name *Chromobacterium* Bergonzini 1880 is conserved and placed in the list of *nomina generum conservanda*.<br>(3) The type species of the genus *Chromobacterium* Bergonzini 1880 is *Chromobacterium violaceum* Bergonzini 1880.<br>(4) A neotype strain of *Chromobacterium violaceum* Bergonzini 1880 is designated and has been deposited in the American Type Culture Collection, Washington, D.C. (ATCC 12472) and in the National Collection of Type Cultures, London (NCTC 9757).

17	Conservation of the generic name *Staphylococcus* Rosenbach, designation of *Staphylococcus aureus* as the nomenclatural type of the genus *Staphylococcus* Rosenbach, and designation of a neotype culture of *Staphylococcus aureus* Rosenbach	*Int Bull Bacteriol Nomencl Taxon* 1958;8:153–154	(1) The generic name *Staphylococcus* Rosenbach 1884 is conserved and placed in the list of *nomina generum conservanda*.<br>(2) *Staphylococcus aureus* Rosenbach 1884 is recognized as the nomenclatural type species of the genus *Staphylococcus* Rosenbach 1884.<br>(3) The strain labeled NCTC 8532 in the National Collection of Type Cultures, London, is designated as the neotype strain of the species *Staphylococcus aureus* Rosenbach 1884.
18	Conservation of *typhi* in the binary combination *Salmonella typhi*	*Int Bull Bacteriol Nomencl Taxon* 1958;8:31–33, see also 1958;8:158–159	The specific epithet *typhi* in the name of the species *Salmonella typhi* (Schroeter) Warren and Scott is conserved over the specific epithet *typhosa* in the name of the species *Salmonella typhosa* (Zopf) White 1930, with the recognition of *Bacillus typhi* Schroeter 1886 as the basonym.
19	Conservation of the generic name *Rickettsia* da Rocha-Lima and of the species name *Rickettsia prowazekii* da Rocha-Lima	*Int Bull Bacteriol Nomencl Taxon* 1958;8:158–159	The generic name *Rickettsia* da Rocha-Lima is conserved against *Stricheria* Stempell, and the specific epithet *prowazekii* in the species name *Rickettsia prowazekii* da Rocha-Lima is conserved against the specific epithet *jurgensi* first used in the species name *Stricheria jurgensi* Stempell.
20	Status of new generic names of bacteria published without names of included species	*Int Bull Bacteriol Nomencl Taxon* 1958;8:160–162	(1) *Name of a hypothetical genus.* A hypothetical genus is one in which no species is described, named, or cited; the existence of the genus is predicated upon the future discovery and description of species as yet unknown. A name applied to a hypothetical genus is not validly published and is to be placed in the list of *nomina rejicienda*.<br>(2) *Name of a "temporary" genus.* A generic name proposed for a genus whose sole function is stated to be to serve as the temporary generic haven for insufficiently described species, which species may be allocated later to an appropriate genus or genera, is not validly published. Such a name may be placed

			in the list of *nomina rejicienda*.<br>(3) *Name of a new genus with a described species that is neither named nor identified with a previously named species.* A new generic name published in a combined description of a genus and species, without the species being named, without citation of a previously and effectively published description of the species, and without subsequent acceptance of the generic name and naming of the species by a later author, should be regarded as not validly published. Such a generic name may be placed in the list of *nomina rejicienda*.<br>However, if a later author has recognized the generic name and has used it with a specific epithet in naming the species described by the first author, particularly if there has been later general acceptance of the name, there may be validation of the generic name as proposed by its author, with the name of the species ascribed to the later author who gave it. Proposals for such validations of names should be made to the Judicial Commission for appropriate action.<br>(4) *Name of a new genus proposed to include one or more previously described and named species, but without simultaneous publication of the new binary combination of generic name and specific epithet.* A published generic name applied to a new genus in which the generic name is not used in a binary combination in naming any species, but in which there is citation of a previously and effectively published description of a species under another name, is regarded as validly published and the consequent *combinationes novae* ascribed likewise to the author of the generic name.
21	Conservation of the generic name *Selenomonas* von Prowazek	*Int Bull Bacteriol Nomencl Taxon* 1958;8:163–165, with type species *Selenomonas sputigena* (Flügge) Boskamp 1922	(1) The generic name *Selenomonas* von Prowazek 1913 was validly published with an accompanying description of the genus.<br>(2) The species *Spirillum sputigenum* Flügge 1886 was characterized and adequate references to description given. The species was assigned to the genus *Selenomonas*.<br>(3) *Selenomonas sputigena* (Flügge) Boskamp 1922 (basonym *Spirillum sputigenum* Flügge) is designated as the type species of *Selenomonas* von Prowazek.<br>(4) The generic name *Selenomonas* von Prowazek 1913 is placed in the list of *nomina generum conservanda*.
22	Status of the generic	*Int Bull Bacteriol Nomencl Taxon*	(1) The generic name *Asterococcus* Borrel, Dujardin-Beaumetz, Jeantet, and Jouan

	name *Asterococcus* and conservation of the generic name *Mycoplasma*	1958;8:166–168, illegitimacy of *Asterococcus* Borrel *et al*. 1910, conservation of *Mycoplasma* Nowak 1929 with type species *Mycoplasma mycoides* (Borrel *et al*.) Freundt 1955	1910 is a later homonym of *Asterococcus* Scherffel 1908 and hence illegitimate.<br>(2) The generic name *Mycoplasma* Nowak 1929 is placed in the list of bacterial *nomina generum conservanda* as the first legitimate generic name proposed to replace *Asterococcus* Borrel *et al*. The type species is *Mycoplasma mycoides* (Borrel *et al*.) Freundt 1955 (basonym *Asterococcus mycoides* Borrel *et al*.).
23	Rejection of the generic names *Nitromonas* Winogradsky 1890 and *Nitromonas* Orla-Jensen 1909, conservation of the generic names *Nitrosomonas* Winogradsky 1892, *Nitrosococcus* Winogradsky 1892, and *Nitrobacter* Winogradsky 1892, and the designation of the type species of these genera	*Int Bull Bacteriol Nomencl Taxon* 1958;8:169–170, **t**ype species are respectively *Nitrosomonas europaea* Winogradsky 1892, *Nitrosococcus nitrosus* (Migula) Buchanan 1925, and *Nitrobacter winogradskyi* Winslow *et al*. 1917	(1) The generic name *Nitromonas* Winogradsky 1890 is placed in the list of *nomina generum rejicienda*.<br>(2) The generic name *Nitromonas* Orla-Jensen 1909 is a later homonym of *Nitromonas* Winogradsky 1890 and a later synonym of *Nitrobacter* Winogradsky (1892). It is placed in the list of *nomina generum rejicienda*.<br>(3) The generic name *Nitrosomonas* Winogradsky 1892 is placed in the list of *nomina generum conservanda* with *Nitrosomonas europaea* Winogradsky 1892 as the nomenclatural type species.<br>(4) The generic name *Nitrosococcus* Winogradsky 1892 is placed in the list of *nomina generum conservanda*, with the species described by Winogradsky and later named *Nitrosococcus nitrosus* (Migula) Buchanan 1925 as the nomenclatural type species.<br>(5) The generic name *Nitrobacter* Winogradsky 1892 is placed in the list of *nomina generum conservanda*, with the species described by Winogradsky and later named *Nitrobacter winogradskyi* Winslow *et al*. 1917 as the nomenclatural type species.
24	Rejection of the generic name *Arthrobacter* Fischer 1895 and conservation of the generic name *Arthrobacter* Conn and Dimmick 1947	*Int Bull Bacteriol Nomencl Taxon* 1958;8:171–172, conservation was effected though its mention was omitted in the Opinion itself. The title of the Opinion explicitly states that *Arthrobacter* Conn and Dimmick is conserved.	(1) The name *Arthrobacter* proposed by Fischer in 1895 as the name of a hypothetical genus of bacteria was not validly published and has no standing in nomenclature.<br>(2) The generic name *Arthrobacter* Conn and Dimmick 1947 was validly published as a *nomen novum*. It is not an emendation of *Arthrobacter* Fischer 1895 nor a later homonym.
25	Rejection of names of bacteria in certain publications of Trécul, Hallier, Billroth, and	*Int Bull Bacteriol Nomencl Taxon* 1963;13:33–35	(1) The specific, subgeneric, generic or other names proposed in the several publications listed below were not validly published as names of taxa of bacteria and have no standing in bacteriological nomenclature. These publications are included in the list of Rejected Publications as authorized in Paragraph 8 under

	Ogston		"Functions of the Judicial Commission," in Section IV of the *International Code of Nomenclature of Bacteria and Viruses*:<br>(a) Trécul A. Production de plantules amylifères dans les cellules végétales pendant la putréfaction. Chlorophylle cristallisée. C. R. Acad. Sci. Paris 1865;61:432–436.<br>(b_1) Hallier, Ernst. Die pflanzlichen Parasiten des menschlichen Körpers für Aerzte, Botaniker und Studierende zugleich als Einleitung in das Stadium der niederen Organismen. Leipzig; 1866.<br>(b_2) Hallier, Ernst. Mikroskopische Untersuchungen. Zwei neue Untersuchungen über den *Micrococcus*. Flora N.S. 1868;26:654–657.<br>(b_3) Hallier E. Mykologische Untersuchungen. III. Untersuchungen der Parasiten beim Tripper, beim weichen Schanker, bei der Syphilis und bei der Rotzkranheit der Pferde. Flora N.S. 1868;26:289–301.<br>(b_4) Hallier, Ernst. Die Parasiten der Infektionskrankheiten. *Z Parasitenkd* 1870;2:113–132.<br>(c) Billroth CAT. Untersuchungen über die Vegetationsformen von *Coccobacteria septica*. Berlin; 1874<br>(d1) Ogston, Alex. Micrococcus poisoning. *J Anat Physiol* 1882;16:526–567.<br>(d2) Ogston, Alex. Micrococcus poisoning (cont.). *J Anat Physiol* 1883;17:24–58.<br>(2) Names proposed in the above-listed publications of Trécul, Hallier, Billroth, and Ogston have in some cases been adopted by later authors as the names of bacterial taxa and one or other of the four authors named cited as author. In such cases the name of the taxon is to be ascribed to the first subsequent authors whose publication meets the requirements of valid publication as prescribed in the *International Code of Nomenclature of Bacteria and Viruses* (Rule 11 [now Rule 27]).
26	Designation of neotype strains (cultures) of type species of the bacterial genera *Salmonella*, *Shigella*, *Arizona*, *Escherichia*, *Citrobacter*, and *Proteus* of the family	*Int Bull Bacteriol Nomencl Taxon* 1963;13:35–36, and 1864;14:57	Neotype cultures of *Salmonella cholerae-suis*, *S. typhi-murium*, *Shigella dysenteriae*, *Arizona arizonae*, *Escherichia coli*, *Citrobacter freundii*, and *Proteus vulgaris* were approved.<br><br>FOR THE CORRECT FORMATTING OF THE GREEN HIGHLIGHTED PARTS OF THE TABLE – DIVISION OF THIS COLUMN INTO MULTIPLE COLUMNS, SEE THE PUBLISHED VERSION OF THE 2022 REVISION

	Enterobacteriaceae		Name of species Catalogue no.<br>NCTC London<br>ATCC Washington<br><br>*Salmonella cholerae-suis* (sic) (Smith) Weldin 1927. Type species of genus *Salmonella* Lignières 1900. 5735 13312<br>*Salmonella typhi-murium* (sic) (Loeffler) Castellani and Chalmers 1919 74 13311<br>*Shigella dysenteriae* (Shiga) Castellani and Chalmers 1919. Type species of genus *Shigella* Castellani and Chalmers 1919. 4837 13313<br>*Arizona arizonae* Kauffmann and Edwards 1952. Type species of genus *Arizona* Kauffmann and Edwards 1952. 8297 13314<br>*Escherichia coli* (Migula) Castellani and Chalmers 1919. Type species of genus *Escherichia* Castellani and Chalmers 1919. 9001 11775<br>*Citrobacter freundii* (Braak) Werkman and Gillen 1932. Type species of genus *Citrobacter* Werkman and Gillen 1932. 9750 8090<br>*Proteus vulgaris* Hauser 1885. Type species of genus *Proteus* Hauser 1885. 4175 13315
27	Designation of the neotype strain of *Streptococcus agalactiae* Lehmann and Neumann	*Int Bull Bacteriol Nomencl Taxon* 1963;13:37	The strain Stableforth G19 is designated as the neotype strain of *Streptococcus agalactiae* Lehmann and Neumann. This neotype strain is catalogued in the National Collection of Type Cultures as NCTC 8181 and in the American Type Culture Collection as ATCC 13813.
28	Rejection of the bacterial generic name *Cloaca* Castellani and Chalmers andacceptance of *Enterobacter* Hormaeche and Edwards as a bacterial generic name with type species *Enterobacter cloacae* (Jordan) Hormaeche and	*Int Bull Bacteriol Nomencl Taxon* 1963;13:38, conservation was effected by statement in the Summary though omitted in the title and in the Opinion itself.	The generic name *Cloaca* Castellani and Chalmers is rejected and replaced by the generic name *Enterobacter* Hormaeche and Edwards with the type species *Enterobacter cloacae* (Jordan) Hormaeche and Edwards: the basonym is *Bacillus cloacae* Jordan.

	Edwards		
29	Designation of strain ATCC 3004 (IMRU 3004) as the neotype strain of *Streptomyces albus* (Rossi Doria) Waksman and Henrici	*Int Bull Bacteriol Nomencl Taxon* 1963;13:123–124	The strain labeled ATCC 3004 in the American Type Culture Collection, Washington, D.C., and also known as IMRU 3004 (Institute of Microbiology, Rutgers University) is designated as the neotype strain of *Streptomyces albus* (Rossi Doria) Waksman and Henrici 1943.
30	Conservation of the specific epithet *faecalis* in the species name *Streptococcus faecalis* Andrewes and Horder 1906	*Int Bull Bacteriol Nomencl Taxon* 1963;13:167	The specific epithet *faecalis* in the species name *Streptococcus faecalis* Andrewes and Horder 1906 is conserved against the specific epithets in *Streptococcus liquefaciens* Sternberg 1892, *S. zymogenes* McCallum and Hastings 1899, and all other earlier synonymous specific epithets in the genus *Streptococcus*.
31	Conservation of *Vibrio* Pacini 1854 as a bacterial generic name, conservation of *Vibrio cholerae* Pacini 1854 as the nomenclatural type species of the bacterial genus *Vibrio*, and designation of neotype strain of *Vibrio cholerae* Pacini	*Int Bull Bacteriol Nomencl Taxon* 1965;15:185–186	*Vibrio cholerae* Pacini 1854 is conserved as the name of the type species of the bacterial genus *Vibrio* Pacini 1854, the bacterial generic name *Vibrio* Pacini 1854 is placed in the list of conserved bacterial generic names (*nomina generum conservanda*), and National Collection of Type Cultures NCTC 8021 (American Type Culture Collection, ATCC 14035) is designated as the neotype of the species *Vibrio cholerae* Pacini 1854.
32	Conservation of the specific epithet *rhusiopathiae* in the scientific name of the organism known as *Erysipelothrix rhusiopathiae* (Migula 1900) Buchanan 1918	*Int J Syst Bacteriol* 1970;20:9	The specific epithet *rhusiopathiae* in the scientific name of the organism known as *Erysipelothrix rhusiopathiae* (Migula 1900) Buchanan 1918 is conserved against the specific epithet *insidiosa* (basonym *Bacillus insidiosus* Trevisan 1885) and against all other specific epithets applied to this organism.
33	Conservation of the	*Int J Syst Bacteriol* 1970;20:10, type	The generic name *Agrobacterium* Conn 1942 is conserved against the name

	generic name *Agrobacterium* Conn 1942	species *Agrobacterium tumefaciens* (Smith and Townsend) Conn 1942	Polymonas Lieske 1928, which is placed in the list of *nomina generum rejicienda*. The type species, by original designation, is *Agrobacterium tumefaciens* (Smith and Townsend 1907) Conn 1942: the basonym is *Bacterium tumefaciens* Smith and Townsend 1907.
34	Conservation of the generic name *Rhizobium* Frank 1889	*Int J Syst Bacteriol* 1970;20:11–12, type species *Rhizobium leguminosarum* Frank 1889	The generic name *Rhizobium* Frank 1889 is conserved against *Phytomyxa* Schroeter 1886 and all earlier synonyms. The type species is *Rhizobium leguminosarum* (Frank 1879) Frank 1889; the basonym is *Schinzia leguminosarum* Frank 1879.
35	Conservation of the specific epithet *meningitidis* in the scientific name of the meningococcus	*Int J Syst Bacteriol* 1970;20:13–14, and designation of neotype strain (genus is now *Neisseria*)	The specific epithet "*meningitidis*" is conserved in the scientific name of the meningococcus (*Diplococcus intracellularis meningitidis* Weichselbaum) against all earlier specific epithets. The neotype strain of this organism is ATCC 13077 (=Sara E. Branham M1027=NCTC 10025).
36	Designation of strain ATCC 10145 as the neotype strain of *Pseudomonas aeruginosa* (Schroeter) Migula	*Int J Syst Bacteriol* 1970;20:15–16	The neotype strain of *Pseudomonas aeruginosa* (Schroeter) Migula is ATCC 10145=CCEB 481=IBCS 277=NCIB 8295=NCTC 10332=NRRL B-771=RH 815.
37	Designation of strain ATCC 13525 as the neotype strain of *Pseudomonas fluorescens* Migula	*Int J Syst Bacteriol* 1970;20:17–18	The neotype strain of *Pseudomonas fluorescens* Migula is ATCC 13525=CCEB 546=NCIB 9046=NCTC 10038=RH 818=M. Rhodes 28/5.
38	Conservation of the generic name *Lactobacillus* Beijerinck	*Int J Syst Bacteriol* 1971;21:104, with new type species *Lactobacillus delbrueckii* Beijerinck 1901 and neotype strain	The generic name *Lactobacillus* Beijerinck 1901 is conserved over *Saccharobacillus* van Laer 1892 and all earlier objective synonyms. The type species of this genus is *Lactobacillus delbrueckii* Beijerinck 1901, the neotype strain of which is ATCC 9649=NCDO213. The name *Lactobacillus delbrueckii* Beijerinck 1901, although used by Beijerinck as a simplified version of the subspecific name "*Lactobacillus fermentum* var. *delbrucki*," shall be held to be validly published by Beijerinck as a species name. The name *Lactobacillus caucasicus* Beijerinck 1901 is placed in the list of rejected names, and *L. caucasicus* ceases to be the type species of *Lactobacillus* Beijerinck.

39	Rejection of the generic name *Gaffkya* Trevisan	*Int J Syst Bacteriol* 1971;21:104–105	The generic name *Gaffkya* Trevisan 1885 is placed on the list of rejected names.
40	Rejection of the names *Mima* De Bord and *Herellea* De Bord and of the specific epithets *polymorpha* and *vaginicola* in *Mima polymorpha* De Bord and *Herellea vaginicola* De Bord, respectively	*Int J Syst Bacteriol* 1971;21:105–107, and loss of standing in nomenclature of the tribal name *Mimeae* De Bord 1939	The generic names *Mima* De Bord 1939, 1942 and *Herellea* De Bord 1942 are placed on the list of rejected names. The specific epithets *polymorpha* and *vaginicola* in *Mima polymorpha* De Bord 1939, 1942 and *Herellea vaginicola* De Bord 1942 respectively are placed on the list of rejected epithets. The tribal name *Mimeae* De Bord 1939, 1942 therefore loses its standing in nomenclature.
41	Conservation of the generic name *Moraxella* Lwoff	*Int J Syst Bacteriol* 1971;21:106, type species *Moraxella lacunata* (Eyre) Lwoff 1939, and neotype strain	The generic name *Moraxella* Lwoff 1939 is conserved over *Diplobacillus* McNab 1904 and over all earlier objective synonyms. The type species is *Moraxella lacunata* (Eyre) Lwoff 1939, and the neotype strain of this species is Morax =ATCC 17967.
42	Conservation of the specific epithet "*phenylpyruvica*" in the name *Moraxella phenylpyruvica* Bøvre and Henriksen	*Int J Syst Bacteriol* 1971;21:107, conservation over epithet polymorpha in the name *Moraxella polymorpha* Flamm 1957, and neotype strain	The specific epithet "*phenylpyruvica*" in the name *Moraxella phenylpyruvica* Bøvre and Henriksen 1967 is conserved against the specific epithet "*polymorpha*" in the name of the earlier objective synonym *Moraxella polymorpha* Flamm 1957 and against the specific epithets in all other earlier objective synonyms. The neotype strain of *Moraxella phenylpyruvica* is 2863 (=ATCC 23333 = NCTC 10526).
43	Conservation of the specific epithet "*sphaeroides*" in the name *Rhodopseudomonas sphaeroides* van Niel	*Int J Syst Bacteriol* 1971;21:108, and neotype strain	The specific epithet "*sphaeroides*" in the name *Rhodopseudomonas sphaeroides* van Niel 1944 is conserved against the specific epithet "*minor*" in the name of the earlier subjective synonym *Rhodococcus minor* and against the specific epithets in the names of all earlier objective synonyms of *Rhodopseudomonas sphaeroides*. The neotype strain is van Niel's ATH 2.4.1 (=ATCC 17023).
44	Validation of the generic name *Chloropseudomonas* Czurda and Maresch 1937 and designation of the type species	*Int J Syst Bacteriol* 1971;21:109, type species *Chloropseudomonas ethylica* Shaposhnikov *et al.* 1960	The generic name *Chloropseudomonas* is held to be validly published by Czurda and Maresch 1937. The type species is *Chloropseudomonas ethylica* Shaposhnikov, Kondratieva, and Fedorov 1960.

45	Rejection of the name *Leuconostoc citrovorum* (Hammer) Hucker and Pederson	*Int J Syst Bacteriol* 1971;21:109–110	The name *Leuconostoc citrovorum* (Hammer 1920) Hucker and Pederson 1931, together with its objective synonyms, is regarded as a *nomen dubium* and is placed on the list of rejected names.
46	Rejection of the generic name *Aerobacter* Beijerinck	*Int J Syst Bacteriol* 1971;21:110	The generic name *Aerobacter* Beijerinck 1900 is regarded as a *nomen ambiguum* and is placed on the list of rejected generic names.
47	Conservation of the specific epithet *avium* in the scientific name of the agent of avian tuberculosis	*Int J Syst Bacteriol* 1973;23:472	The specific epithet *avium* is conserved against the specific epithet *tuberculosis-gallinarum* and all earlier objective synonyms in the scientific name of the agent of avian tuberculosis. The name *Mycobacterium avium* shall be held to be validly published by Chester in 1901. The neotype strain of *M. avium* Chester is ATCC 25291.
48	Rejection of the name *Aerobacter liquefaciens* Beijerinck and conservation of the name *Aeromonas* Stanier with *Aeromonas hydrophila* as the type species	*Int J Syst Bacteriol* 1973;23:473–474	The name *Aerobacter liquefaciens* Beijerinck 1900 is a *nomen dubium* and, together with all objective synonyms of this name, is placed on the list of rejected names. The generic name *Aeromonas* Stanier 1943, with type species *Aeromonas hydrophila* (Chester 1901) Stanier 1943, is conserved. The name *Aeromonas* is not to be attributed to Kluyver and van Niel. The neotype strain of *A. hydrophila* is ATCC 7966.
49	Conservation of the generic name *Rhodopseudomonas* Czurda and Maresch emend. van Niel	*Int J Syst Bacteriol* 1974;24:551	The generic name *Rhodopseudomonas* Czurda and Maresch 1937 emend. van Niel 1944 is conserved over all earlier objective synonyms; the type species is *Rhodopseudomonas palustris* (Molisch 1907) van Niel 1944 (basonym *Rhodobacillus palustris* Molisch 1907).
50	Conservation of the epithet *fermentum* in the combination *Lactobacillus fermentum* Beijerinck	*Int J Syst Bacteriol* 1974;24:551–552	The species name *Lactobacillus fermentum* Beijerinck 1901 shall be held to be validly published by Beijerinck 1901 as the name of a bacterial species, and the epithet *fermentum* in the combination *Lactobacillus fermentum* Beijerinck 1901 is conserved over the epithets in all other objective synonyms. The neotype strain of *Lactobacillus fermentum* is ATCC 4931.
51	Conservation of the epithet *fortuitum* in the combination	*Int J Syst Bacteriol* 1974;25:552	The specific epithet *fortuitum* in the name *Mycobacterium fortuitum* da Costa Cruz 1938 is conserved against the epithet *ranae* in the subjective synonym *Mycobacterium ranae* Bergey *et al.* 1923 and against the specific epithets in the

	Mycobacterium fortuitum da Costa Cruz		names of all objective synonyms of *Mycobacterium fortuitum* and *Mycobacterium ranae*. The type strain of *Mycobacterium fortuitum* is ATCC 6841.
52	Conservation of the generic name *Pediococcus* Claussen with the type species *Pediococcus damnosus* Claussen	*Int J Syst Bacteriol* 1976;26:292, replacement of type species *P. cerevisiae* by *P. damnosus*	The generic name *Pediococcus* Claussen 1903 is conserved over *Pediococcus* Balcke 1884 and all earlier objective synonyms. The type species is *Pediococcus damnosus* Claussen 1903, and the neotype strain is Be.l (=NCDO 1832). *Pediococcus* Balcke 1884 and the species name *Pediococcus cerevisiae* Balcke 1884 are not validly published.
53	Rejection of the species name *Mycobacterium marianum* Penso 1953	*Int J Syst Bacteriol* 1978;28:334, confusion between the epithets *marianum* and *marinum*	The species name *Mycobacterium marianum* Penso 1953 is placed on the list of *nomina rejicienda* as a *nomen perplexum* because it is a source of confusion.
54	Rejection of the species name *Pseudomonas denitrificans* (Christensen) Bergey *et al*. 1923	*Int J Syst Bacteriol* 1982;32:466	The species name *Pseudomonas denitrificans* (Christensen) Bergey *et al*. 1923 is placed on the list of *nomina rejicienda* as a *nomen ambiguum* because it is a source of confusion.
55	Rejection of the species name *Mycobacterium aquae* Jenkins *et al*. 1972	*Int J Syst Bacteriol* 1982;32:467	The species name *Mycobacterium aquae* Jenkins *et al*. 1972 is placed on the list of *nomina rejicienda* as a *nomen ambiguum* because it is a source of confusion.
56	Rejection of the species name *Peptococcus anaerobius* (Hamm) Douglas 1957	*Int J Syst Bacteriol* 1982;32:468	The species name *Peptococcus anaerobius* (Hamm) Douglas 1957 is placed on the list of *nomina rejicienda* as a *nomen dubium* and a *nomen perplexum* because it is a source of confusion.
57	Designation of *Eubacterium limosum* (Eggerth) Prévot 1938 as the type species of *Eubacterium*	*Int J Syst Bacteriol* 1983;33:434, replacement of type species *E. foedans* by *E. limosum*	The type species of the genus *Eubacterium* Prévot 1938 is designated *E. limosum* (Eggerth) Prévot 1938 (type strain, ATCC 8486).
58	Confirmation of the type species in the Approved Lists as nomenclatural types including	*Int J Syst Bacteriol* 1985;35:538, confirmation of new type species for *Nocardia* and *Pasteurella* (see Opinion 13) and rejection of *P.*	The names (*Editorial Note*. This should read “The types.”) of the bacterial taxa cited in the Approved Lists of Bacterial Names are formally and explicitly confirmed as correct and supersede any others in use before the appearance of the lists but without prejudice to the powers of the Judicial Commission to amend

	recognition of *Nocardia asteroides* (Eppinger 1891) Blanchard 1896 and *Pasteurella multocida* (Lehmann and Neumann 1899) Rosenbusch and Marchant 1939 as the respective type species of the genera *Nocardia* and *Pasteurella* and rejection of the type species name *Pasteurella gallicida* (Burrill 1883) Buchanan 1925	*gallicida* as an objective synonym of *P. multocida* (*Editorial Note*: As stated in the title and summary, the Opinion also confirms the nomenclatural types in the Approved Lists, but without prejudice to the powers of the Judicial Commission to amend them.)	them. The species names *Nocardia asteroides* (Eppinger 1891) Blanchard 1896 and *Pasteurella multocida* (Lehmann and Neumann 1899) Rosenbusch and Marchant 1939 are the valid type species of their respective genera, thus reversing those elements of Opinion 13 that apply to these two genera. The species name *Pasteurella gallicida* (Burrill 1883) Buchanan 1925 is placed on the list of *nomina rejicienda*.
59	Designation of NCIB 11664 in place of ATCC 23767 (NCIB 4112) as the type strain of *Acetobacter aceti* subsp. *xylinum* (sic) (Brown 1886) De Ley and Frateur 1974	*Int J Syst Bacteriol* 1985;35:539. The epithet *xylinum* should be spelled *xylinus* (see Opinion 3).	The type strain of *Acetobacter aceti* subsp. *xylinus* is NCIB 11664 (=NCIB 4112B) not ATCC 23767 (=NCIB 4112=NCIB 11301=CIP 57.14).
60	Rejection of the name *Yersinia pseudotuberculosis* subsp. *pestis* (van Loghem) Bercovier *et al.* 1981 and conservation of the name *Yersinia pestis* (Lehmann and Neumann) van Loghem	*Int J Syst Bacteriol* 1985;35:540, see also Rule 56a(5)	The name *Yersinia pseudotuberculosis* subsp. *pestis* (van Loghem) Bercovier *et al.* 1981 is placed on the list of *nomina rejicienda* because the use of the name could have serious consequences for human welfare and health. The name *Yersinia pestis* is conserved for the plague bacillus. The opinion does not challenge the scientific evidence, which indicates the taxonomic relatedness of bacteria named *Yersinia pestis* and *Yersinia pseudotuberculosis*.

	1944 for the plague bacillus		
61	Rejection of the type strain of *Pasteuria ramosa* (ATCC 27377) and conservation of the species *Pasteuria ramosa* Metchnikoff 1888 on the basis of the type descriptive material	*Int J Syst Bacteriol* 1986;36:119	Strain ATCC 27377 is rejected as the type strain of the species *Pasteuria ramosa* Metchnikoff 1888 because it is quite different from the bacteria observed and described by Metchnikoff and to which he gave the name *Pasteuria ramosa*: *Pasteuria ramosa* is conserved with the description of Metchnikoff, as amended by Starr *et al*. 1983, serving as the type species. (*Editorial Note*. This should read "serving as the type.")<br>In issuing this opinion, the Judicial Commission declines to comment on the assignment of strain ATCC 27377 to another genus because this is a taxonomic matter and not one of nomenclature.
62	Transfer of the type species of the genus *Methanococcus* to the genus *Methanosarcina* as *Methanosarcina mazei* (Barker 1936) comb. nov. et emend. Mah and Kuhn 1984 and conservation of the genus *Methanococcus* (Approved Lists 1980) emend. Mah and Kuhn 1984 with *Methanococcus vannielii* (Approved Lists 1980) as the type species	*Int J Syst Bacteriol* 1986;36:491	*Methanococcus mazei*, the type species of the genus *Methanococcus*, is transferred to the genus *Methanosarcina* as *Methanosarcina mazei* (Barker 1936) comb. nov. et emend. Mah and Kuhn 1984. The genus *Methanococcus* (Approved Lists 1980) emend. Mah and Kuhn 1984 is conserved with *Methanococcus vannielii* Stadtman and Barker 1951 (Approved Lists 1980) as the type species.
63	Rejection of the type species *Methanosarcina methanica* (Approved Lists 1980) and conservation of the genus *Methanosarcina*	*Int J Syst Bacteriol* 1986;36:492	*Methanosarcina methanica* (Approved Lists 1980), the nomenclatural type species of the genus *Methanosarcina* (Approved Lists 1980), is placed on the list of *nomina rejicienda* as a *nomen dubium et confusum* because it is a source of doubt and confusion. The genus *Methanosarcina* (Approved Lists 1980) emend. Mah and Kuhn 1984 is conserved with *Methanosarcina barkeri* (Approved Lists 1980) as the type species.

	(Approved Lists 1980) emend. Mah and Kuhn 1984 with *Methanosarcina barkeri* (Approved Lists 1980) as the type species		
64	Designation of strain MF (DSM 1535) in place of strain M.o.H. (DSM 863) as the type strain of *Methanobacterium formicicum* Schnellen 1947, and designation of strain M.o.H. (DSM 863) as the type strain of *Methanobacterium bryantii* (Balch and Wolfe in Balch, Fox, Magrum, Woese, and Wolfe 1979, 284) Boone 1987, 173	*Int J Syst Bacteriol* 1992;42:654; doi:10.1099/00207713-42-4-654	The type strain of *Methanobacterium formicicum* is strain MF (DSM 1535), replacing strain M.o.H. (DSM 863). *Methanobacterium bryantii* is reinstated with its type strain M.o.H. (DSM 863).
65	Designation of strain VPI D 19B-28 (ATCC 35185) in place of strain VPI 10068 (ATCC 33150) as the type strain of *Selenomonas sputigena* (Flügge 1886) Boskamp 1922	*Int J Syst Bacteriol* 1992;42:655; doi:10.1099/00207713-42-4-655	The type strain of *Selenomonas sputigena* is VPI D 19B-2 (ATCC 35185), replacing VPI 10068 (ATCC 33150). (NB VPI D 19B-28 is the correct number, not VPI D 19B-29, which is given in the ATCC catalog, 17th ed.).
66	Designation of strain NS 51 (NCTC 12261) in place of strain NCTC 3165 as the type strain of	*Int J Syst Bacteriol* 1993;43:391; doi:10.1099/00207713-43-2-391	The type strain of *Streptococcus mitis* is NS 51 (NCTC 12261), replacing NCTC 3165.

	Streptococcus mitis Andrewes and Horder 1906		
67	Rejection of the name *Citrobacter diversus* Werkman and Gillen 1932	*Int J Syst Bacteriol* 1993;43:392; doi:10.1099/00207713-43-2-392	The name *Citrobacter diversus* Werkman and Gillen 1932 is placed on the list of *nomina rejicienda* because it was incorrectly used by Ewing and Davis in 1972 as the name for a new species that cannot be considered identical to the organism described by Werkman and Gillen and thus is a *nomen dubium*.
68	Designation of strain B213c (DSM 20284) in place of Strain NCDO 1859 as the type strain of *Pediococcus acidilactici* Lindner 1887	*Int J Syst Bacteriol* 1996;46:835; doi:10.1099/00207713-46-3-835	*Pediococcus acidilactici* is conserved with neotype strain B213c (=DSM 20284), which replaces NCDO 1859.
69	Rejection of *Clostridium putrificum* and conservation of *Clostridium botulinum* and *Clostridium sporogenes*	*Int J Syst Bacteriol* 1999;49:339; doi:10.1099/00207713-49-1-339	The name *Clostridium putrificum* is rejected while *Clostridium botulinum* is conserved for toxigenic strains and *Clostridium sporogenes* is conserved for nontoxigenic strains.
70	Replacement of strain NCTC 4175, since 1963 the neotype strain of *Proteus vulgaris*, with strain ATCC 29905	*Int J Syst Bacteriol* 1999;49:1949; doi:10.1099/00207713-49-4-1949	The Judicial Commission decided that strain NCTC 4175, used as the neotype strain of *Proteus vulgaris* since 1963, be replaced by strain ATCC 29905.
71	Valid publication of the genus name *Thermodesulfobacterium* and the species names *Thermodesulfobacterium commune* Zeikus *et al.* 1983 and *Thermodesulfobacteriu*	*Int J Syst Evol Microbiol* 2003;53:927; doi:10.1099/ijs.0.02494-0	The Judicial Commission of the International Committee on Systematics of Prokaryotes decided that the date of valid publication of the genus name *Thermodesulfobacterium* and of the species names *Thermodesulfobacterium commune* and *Thermodesulfobacterium thermophilum* is 1995. *Thermodesulfobacterium mobile* Rozanova and Pivovarova 1988 is an illegitimate, later synonym of *Thermodesulfobacterium thermophilum*.

	m thermophilum (*ex Desulfovibrio thermophilus* Rozanova and Khudyakova 1974)		
72	Strain DSM 6035 is the type strain of *Lactobacillus panis* Wiese *et al*. 1996	*Int J Syst Evol Microbiol* 2003;53:920; doi:10.1099/ijs.0.02495-0	The Judicial Commission of the International Committee on Systematics of Prokaryotes decided that strain DSM 6035 is the type strain of *Lactobacillus panis* with the consequence that the name *Lactobacillus panis* has been validly published.
73	*Paenibacillus durus* (Collins *et al*. 1994, formerly *Clostridium durum* Smith and Cato 1974) has priority over *Paenibacillus azotofixans* (Seldin *et al*. 1984)	*Int J Syst Evol Microbiol* 2003;53:931; doi:10.1099/ijs.0.02496-0	The Judicial Commission adjusted the gender of the specific epithet to *durus* (masculine) and decided that the name *Paenibacillus durus* has priority over *Paenibacillus azotofixans*; furthermore, it was decided that the type strain of *Paenibacillus durus* is VPI 6563 (=ATCC 27763=DSM 1735), not P3L5 (=ATCC 35681). The name *Paenibacillus azotofixans* is a later synonym of *Paenibacillus durus*.
74	Strain NCIMB 13488 may serve as the type strain of *Halorubrum trapanicum*	*Int J Syst Evol Microbiol* 2003;53:933; doi:10.1099/ijs.0.02497-0	The Judicial Commission decided that *Halorubrum trapanicum* strain NCIMB 13488 will not be the neotype, but since it is derived from strain NRC 34021, which in turn is derived from Petter's original isolate, it is 'a strain on which the original description was based' [Rule 18c of the *Bacteriological Code* (1990 Revision); Lapage *et al*., 1992], and may therefore also serve as the type strain of the species.
75	Rejection of the genus name *Methanothrix* with the species *Methanothrix soehngenii* Huser *et al*. 1983 and transfer of *Methanothrix thermophila* Kamagata *et al*. 1992 to the genus *Methanosaeta* as *Methanosaeta thermophila* comb. nov.	*Int J Syst Evol Microbiol* 2008;58:1753–1754; doi:10.1099/ijs.0.2008/005355-0	The Judicial Commission of the International Committee on Systematics of Prokaryotes has decided to place the genus *Methanothrix* with the species *Methanothrix soehngenii* Huser *et al*. 1983 on the list of *nomina rejicienda*, based on the fact that it is not represented by an axenic culture and contravenes Rule 31a of the *International Code of Nomenclature of Bacteria*. The species *Methanothrix thermophila* is transferred to the genus *Methanosaeta* as *Methanosaeta thermophila* (Kamagata *et al*. 1992) Boone and Kamagata 1998 comb. nov.

75 (suppl.)	The genus name *Methanothrix* Huser *et al*. 1983 and the species combination *Methanothrix soehngenii* Huser *et al*. 1983 do not contravene Rule 31a and are not to be considered as rejected names, the genus name *Methanosaeta* Patel and Sprott 1990 refers to the same taxon as *Methanothrix soehngenii* Huser *et al*. 1983 and the species combination *Methanothrix thermophila* Kamagata *et al*. 1992 is rejected	*Int J Syst Evol Microbiol* 2014;64:3597–3598; doi:10.1099/ijs.0.069252-0	The Judicial Commission affirms that the genus name *Methanothrix* Huser *et al*. 1983 and the species combination *Methanothrix soehngenii* Huser *et al*. 1983 do not contravene Rule 31a and are not to be considered as rejected names. The genus name *Methanosaeta* Patel and Sprott 1990 applies to the same taxon as *Methanothrix* Huser *et al*. 1983 and is therefore a later heterotypic synonym. The combinations *Methanothrix thermoacetophila* corrig. Nozhevnikova and Chudina 1988 and *Methanothrix thermophila* Kamagata *et al*. 1992 are considered to refer to the same taxon, a consequence of which is that *Methanothrix thermophila* Kamagata *et al*. 1992 contravenes Rule 51b and is placed on the List of Rejected Names.
76	Strain NBRC (formerly IFO) 3782 is the type strain of *Streptomyces rameus* Shibata 1959	*Int J Syst Evol Microbiol* 2005;55:511; doi:10.1099/ijs.0.63545-0	The Judicial Commission of the International Committee for Systematics of Prokaryotes decided that strain NBRC (formerly IFO) 3782 (=No. 43797), which was the originally designated type strain, has to replace ATCC 21273 as the type strain of *Streptomyces rameus*. ATCC 21273 was given as the type strain in the Approved Lists 1980.
77	The type species of the genus *Paenibacillus* Ash *et al*. 1994 is *Paenibacillus polymyxa*	*Int J Syst Evol Microbiol* 2005;55:513; doi:10.1099/ijs.0.63546-0	The Judicial Commission of the International Committee for Systematics of Prokaryotes decided that the type species of the genus *Paenibacillus* is *Paenibacillus polymyxa*.
78	Rejection of the genus name *Pelczaria* with the species *Pelczaria*	*Int J Syst Evol Microbiol* 2005;55:515; doi:10.1099/ijs.0.63547-0	The Judicial Commission of the International Committee for Systematics of Prokaryotes has decided to place the genus *Pelczaria* with the species *Pelczaria aurantia* on the list of *nomina rejicienda*, due to the lack of an authentic type or

	aurantia Poston 1994		neotype strain.
79	The nomenclatural types of the orders *Acholeplasmatales*, *Halanaerobiales*, *Halobacteriales*, *Methanobacteriales*, *Methanococcales*, *Methanomicrobiales*, *Planctomycetales*, *Prochlorales*, *Sulfolobales*, *Thermococcales*, *Thermoproteales* and *Verrucomicrobiales* are the genera *Acholeplasma*, *Halanaerobium*, *Halobacterium*, *Methanobacterium*, *Methanococcus*, *Methanomicrobium*, *Planctomyces*, *Prochloron*, *Sulfolobus*, *Thermococcus*, *Thermoproteus* and *Verrucomicrobium*, respectively	*Int J Syst Evol Microbiol* 2005;55:517–518; doi:10.1099/ijs.0.63548-0	The Judicial Commission corrected the nomenclatural types of twelve orders, for which, in violation of Rules 15 and 21a of the *Bacteriological Code* (1990 Revision), families instead of genera had been proposed as nomenclatural types. The following orders have the following genera as nomenclatural types: order *Acholeplasmatales* Freundt *et al.* 1984, genus *Acholeplasma* Edward and Freundt 1970 (Approved Lists 1980); *Halanaerobiales* Rainey and Zhilina 1995, *Halanaerobium* Zeikus *et al.* 1984; *Halobacteriales* Grant and Larsen 1989, *Halobacterium* Elazari-Volcani 1957 (Approved Lists 1980); *Methanobacteriales* Balch and Wolfe 1981, *Methanobacterium* Kluyver and van Niel 1936 (Approved Lists 1980); *Methanococcales* Balch and Wolfe 1981, *Methanococcus* Kluyver and van Niel 1936 emend. Barker 1936 (Approved Lists 1980); *Methanomicrobiales* Balch and Wolfe 1981, *Methanomicrobium* Balch and Wolfe 1981; *Planctomycetales* Schlesner and Stackebrandt 1987, *Planctomyces* Gimesi 1924 (Approved Lists 1980); *Prochlorales* (*ex* Lewin 1977) Florenzano *et al.* 1986, *Prochloron* (*ex* Lewin 1977) Florenzano *et al.* 1986; *Sulfolobales* Stetter 1989, *Sulfolobus* Brock *et al.* 1972 (Approved Lists 1980); *Thermococcales* Zillig *et al.* 1988, *Thermococcus* Zillig 1983; *Thermoproteales* Zillig and Stetter 1982, *Thermoproteus* Zillig and Stetter 1982; *Verrucomicrobiales* Ward-Rainey *et al.* 1996, *Verrucomicrobium* Schlesner 1988.
79 (suppl.)	Names at the rank of class, subclass and order, their typification and current status	*Int J Syst Evol Microbiol* 2014;64:3599–3602; doi:10.1099/ijs.0.069310-0	The attention of the Judicial Commission was drawn to issues relating to the use of names at the rank of class, subclass and order and the nomenclatural type of names at the rank of class and subclass that were not covered by Opinion 79. The Judicial Commission ruled that names at the rank of class and order proposed by Cavalier-Smith (*Int J Syst Evol Microbiol* 2002;52:7–76) are placed on the List of

			Rejected Names (*nomina rejicienda*) and the use of names proposed in that publication above the rank of class is actively discouraged. In addition, a list of names at the rank of class, subclass and order is given where the nomenclatural type, description or circumscription is unclear or where they otherwise appear to be not in accordance with the Rules of the *International Code of Nomenclature of Bacteria*.
80	The type species of the genus *Salmonella* Lignieres 1900 is *Salmonella enterica* (*ex* Kauffmann and Edwards 1952) Le Minor and Popoff 1987, with the type strain LT2^T, and conservation of the epithet *enterica* in *Salmonella enterica* over all earlier epithets that may be applied to this species	*Int J Syst Evol Microbiol* 2005;55:519–520; doi:10.1099/ijs.0.63579-0	The Judicial Commission of the International Committee for Systematics of Prokaryotes has decided that the type species of the genus *Salmonella* Lignieres 1900 is *Salmonella enterica* (*ex* Kauffmann and Edwards 1952) Le Minor and Popoff 1987 and that the type strain of this species is strain LT2^T. In addition, the epithet *enterica* in *Salmonella enterica* is conserved over all earlier epithets that may be applied to this species.<br>The Judicial Commission is aware that this Opinion has consequences for the nomenclature and taxonomy of this group of organisms. Refer to accompanying commentary and references in the Opinion.
81	Status of strains that contravene Rules 27 (3) and 30 of the *International Code of Nomenclature of Bacteria*	*Int J Syst Evol Microbiol* 2008;58:1755–1763; doi:10.1099/ijs.0.2008/005264-0	Based on a list of 205 names proposed in original articles in the *International Journal of Systematic and Evolutionary Microbiology* or cited in Validation Lists from January 2001 that are not in accordance with Rules 27(3) and 30 of the *International Code of Nomenclature of Bacteria* (the *Code*), the Judicial Commission rules that names contained in lists 2–4 are considered to be validly published and that deposit in more than one collection in different countries is documented. Names included in list 1 are only considered validly published if evidence is presented that the strains have been deposited in additional collections, as laid down by Rules 27(3) and 30 of the *Code*.
82	The type strain of *Lactobacillus casei* is ATCC 393, ATCC 334 cannot serve as the type	*Int J Syst Evol Microbiol* 2008;58:1764–1765; doi:10.1099/ijs.0.2008/005330-0	The Judicial Commission affirms that typification of *Lactobacillus casei* is based on ATCC 393, that ATCC 334 is a member of a different taxon and that the publication rejecting the name *Lactobacillus paracasei* (and its included subspecies) together with the revival of the name '*Lactobacillus zeae*' contravenes Rules 51b (1) and (2)

	because it represents a different taxon, the name *Lactobacillus paracasei* and its subspecies names are not rejected and the revival of the name '*Lactobacillus zeae*' contravenes Rules 51b (1) and (2) of the *International Code of Nomenclature of Bacteria*		of the *International Code of Nomenclature of Bacteria*.
83	The subgenus names *Moraxella* subgen. *Moraxella* and *Moraxella* subgen. *Branhamella* and the species names included within these taxa should have been included on the Approved Lists of Bacterial Names and a ruling on the proposal to make changes to Rule 34a	*Int J Syst Evol Microbiol* 2008;58:1766–1767; doi:10.1099/ijs.0.2008/005272-0	The Judicial Commission of the International Committee for Systematics of Prokaryotes rules that the following names should have been included on the Approved Lists of Bacterial Names, *Moraxella* (subgen. *Branhamella* Bøvre 1979), *Moraxella* (subgen. *Moraxella* Lwoff 1939), *Moraxella* (subgen. *Branhamella* Bøvre 1979) *catarrhalis*, Moraxella (subgen. *Branhamella* Bøvre 1979) *caviae*, *Moraxella* (subgen. *Branhamella* Bøvre 1979) *ovis*, *Moraxella* (subgen. *Moraxella* Lwoff 1939) *atlantae*, *Moraxella* (subgen. *Moraxella* Lwoff 1939) *bovis*, *Moraxella* (subgen. *Moraxella* Lwoff 1939) *lacunata*, *Moraxella* (subgen. *Moraxella* Lwoff 1939) *nonliquefaciens*, *Moraxella* (subgen. *Moraxella* Lwoff 1939) *osloensis*, *Moraxella* (subgen. *Moraxella* Lwoff 1939) *phenylpyruvica*. Proposals to alter Rule 34a were rejected.
83 (suppl.)	The subgenus names *Moraxella* and *Branhamella* (in the genus *Moraxella*) are not in accordance with the *International Code of Nomenclature of*	*Int J Syst Evol Microbiol* 2014;64:3595–3596; doi:10.1099/ijs.0.069245-0	The publication of Opinion 83, which dealt with the valid publication of the subgenus names *Moraxella* and *Branhamella* (in the genus *Moraxella*), has highlighted a problem relating to the absence of descriptions associated with these names at the time they were effectively published. This calls into question whether the ruling outlined in Opinion 83, that these names should have qualified for inclusion on the Approved Lists of Bacterial Names, and their inclusion on Validation List 15 are not in accordance with Rule 27 of the *International Code of*

	Bacteria and are therefore not validly published		*Nomenclature of Bacteria* governing the valid publication of a name. The subgenus names *Moraxella* and *Branhamella* (in the genus *Moraxella*) are not to be considered to be included on the Approved Lists of Bacterial Names, nor are they to be considered to be validly published by inclusion on Validation List 15.
84	The genus name *Sinorhizobium* Chen *et al*. 1988 is a later synonym of *Ensifer* Casida 1982 and is not conserved over the latter genus name, and the species name '*Sinorhizobium adhaerens*' is not validly published	*Int J Syst Evol Microbiol* 2008;58:1973; doi:10.1099/ijs.0.2008/005991-0	The Judicial Commission affirms that the genus name *Sinorhizobium* Chen *et al*. 1988 is a later synonym of *Ensifer* Casida 1982, and that the former genus name is not conserved over the latter genus name. The species name '*Sinorhizobium adhaerens*' is not validly published.
85	The adjectival form of the epithet in *Tannerella forsythensis* Sakamoto *et al*. 2002 is-retained and the name is corrected to *Tannerella forsythia* Sakamoto *et al*. 2002	*Int J Syst Evol Microbiol* 2008;58:1974; doi:10.1099/ijs.0.2008/006007-0	The Judicial Commission rules that the adjectival form is conserved in the specific epithet *forsythia* in *Tannerella forsythia*.
86	Necessary corrections to the Approved Lists of Bacterial Names according to Rule 40d (formerly Rule 46)	*Int J Syst Evol Microbiol* 2008;58:1975; doi:10.1099/ijs.0.2008/006015-0	The Judicial Commission affirms that, according to Rule 40d, formerly Rule 46, of the *Bacteriological Code*, the authorship of a number of subspecies names included on the Approved Lists of Bacterial Names must be corrected. These names are *Acetobacter aceti* subsp. *aceti*, *Acetobacter pasteurianus* subsp. *pasteurianus*, *Bacteroides melaninogenicus* subsp. *melaninogenicus*, *Campylobacter fetus* subsp. *fetus*, *Mycobacterium chelonae* subsp. *chelonae*, *Propionibacterium freudenreichii* subsp. *freudenreichii*, *Selenomonas ruminantium* subsp. *ruminantium*, *Streptoverticillium fervens* subsp. *fervens*, *Veillonella parvula* subsp. *parvula* and *Zymomonas mobilis* subsp. *mobilis*.
87	*Corynebacterium ilicis* is	*Int J Syst Evol Microbiol* 2008;58:	The Judicial Commission rules that the name *Corynebacterium ilicis* Mandel *et al*.

	typified by ICMP 2608 =ICPB CI144, *Arthrobacter ilicis* is typified by DSM 20138 =ATCC 14264 =NCPPB 1228 and the two are not homotypic synonyms, and clarification of the authorship of these two species	1976–1978; doi:10.1099/ijs.0.2008/006221-0	1961 is represented by the type strain ICMP 2608 =ICPB CI144 and is reported to be a plantpathogenic species. *Arthrobacter ilicis* is represented by the type strain DSM 20138 =ATCC 14264 =NCPPB 1228 and is not a homotypic synonym of *Corynebacterium ilicis* Mandel *et al.* 1961, and is reported not to be a plant pathogen. The authorship is to be cited as *Arthrobacter ilicis* Collins *et al.* 1982 and typification and the description of this species are found in Collins *et al.* (1981) [Collins MD, Jones D, Kroppenstedt RM. *Zentralbl Bakteriol Parasitenkd Infektionskr Hyg Abt I Orig C* 1981;2:318–323].
88	The status of the name *Lactobacillus rogosae* Holdeman and Moore 1974	*Int J Syst Evol Microbiol* 2014;64:3578–3579; doi:10.1099/ijs.0.069146-0	The Judicial Commission affirms that the combination *Lactobacillus rogosae* Holdeman and Moore 1974 represented by the type strain ATCC 27753 listed on the Approved Lists of Bacterial Names does not appear to be currently represented by an extant type strain. Further work is needed to determine whether a derivative of the original type can be found or whether a neotype can be designated.
89	The epithet *aurantiaca* in *Micromonospora aurantiaca* Sveshnikova *et al.* 1969 (Approved Lists 1980) is illegitimate and requires a replacement epithet	*Int J Syst Evol Microbiol* 2014;64:3580–3581; doi:10.1099/ijs.0.069153-0	The Judicial Commission affirms that the combination *Micromonospora aurantiaca* Sveshnikova *et al.* 1969 (Approved Lists 1980) may not serve as the correct name of the taxon because Rule 12b states that no specific or subspecific epithets within the same genus may be the same if based on different types and the specific epithet *aurantiaca* in *Micromonospora aurantiaca* Sveshnikova *et al.* 1969 (Approved Lists 1980) is the same as the subspecific epithet *aurantiaca* in *Micromonospora carbonacea* subsp. *aurantiaca* Luedemann and Brodsky 1964 (Approved Lists 1980) and the latter has priority. According to Rule 53, the duplication of the same specific or subspecific epithet based on different types creates an illegitimate epithet with the principle of priority determining which is to be replaced as specified in Rule 54. The replacement of the specific epithet aurantiaca in *Micromonospora aurantiaca* Sveshnikova *et al.* 1969 (Approved Lists 1980) also requires that the authorship of the original authors is retained. However, action of this nature requires that the original epithet is maintained in the original combination. There currently appears to be no mechanisms where such action can be taken.

90	The combination *Enterobacter agglomerans* is to be cited as *Enterobacter agglomerans* (Beijerinck 1888) Ewing and Fife 1972 and the combination *Pantoea agglomerans* is to be cited as *Pantoea agglomerans* (Beijerinck 1888) Gavini *et al*. 1989	*Int J Syst Evol Microbiol* 2014;64:3582–3583; doi:10.1099/ijs.0.069161-0	The Judicial Commission affirms that, according to information presented to it, the combination *Enterobacter agglomerans* is to be cited as *Enterobacter agglomerans* (Beijerinck 1888) Ewing and Fife 1972 and the combination *Pantoea agglomerans* is to be cited as *Pantoea agglomerans* (Beijerinck 1888) Gavini *et al*. 1989.
91	ATCC 43642 replaces ATCC 23581 as the type strain of *Leptospira interrogans* (Stimson 1907) Wenyon 1926	*Int J Syst Evol Microbiol* 2014;64:3584–3585; doi:10.1099/ ijs.0.069179-0	The Judicial Commission affirms that, according to information presented to it, the type strain of *Leptospira interrogans* (Stimson 1907) Wenyon 1926 designated on the Approved Lists of Bacterial Names (ATCC 23581) has been shown not to represent an authentic culture of strain RGA (a member of the serovar Icterohaemorrhagiae) and ATCC 43642, derived from an authentic strain of strain RGA, a member of the serovar Icterohaemorrhagiae, is designated the type strain of *Leptospira interrogans* (Stimson 1907) Wenyon 1926.
92	The Request for an Opinion that the current use of the genus name *Mycoplasma* be maintained and *Mycoplasma coccoides* be considered a legitimate name is denied	*Int J Syst Evol Microbiol* 2014;64:3586–3587; doi:10.1099/ijs.0.069187-0	The Judicial Commission affirms that the request that the current use of the genus name *Mycoplasma* be maintained and *Mycoplasma coccoides* be considered a legitimate name is denied.
93	The designated type strain of *Pseudomonas halophila* Fendrich 1989 is DSM 3051, the designated type strain of	*Int J Syst Evol Microbiol* 2014;64:3588–3589; doi:10.1099/ijs.0.069195-0	The Judicial Commission affirms that, according to information presented to it, the designated type strain of *Pseudomonas halophila* Fendrich 1989 is DSM 3051 (replacing DSM 3050) and the designated type strain of *Halovibrio variabilis* Fendrich 1989 is DSM 3050 (replacing DSM 3051). A new name, “*Halomonas utahensis*” (Fendrich 1989) Sorokin and Tindall 2006 nom. nov., is created for the

	Halovibrio variabilis Fendrich 1989 is DSM 3050, a new name *Halomonas utahensis* (Fendrich 1989) Sorokin and Tindall 2006 is created for DSM 3051 when treated as a member of the genus *Halomonas*, the combination *Halomonas variabilis* (Fendrich 1989) Dobson and Franzmann 1996 is rejected, the combination *Halovibrio denitrificans* Sorokin *et al*. 2006 is validly published with an emendation of the description of the genus *Halovibrio* Fendrich 1989 emend. Sorokin *et al.* 2006		species represented by DSM 3051 when treated as a member of the genus *Halomonas*, because the combination *Halomonas halophila* (Quesada *et al.* 1984) Dobson and Franzmann 1996 has priority based on the fact that the epithet *halophila* in the combination *Halomonas halophila* (Quesada *et al*. 1984) Dobson and Franzmann 1996 (basonym *Deleya halophila* Quesada *et al*. 1984) has priority over the epithet halophila should the taxon *Pseudomonas halophila* Fendrich 1989 be treated as a member of the genus *Halomonas*. The combination *Halomonas variabilis* (Fendrich 1989) Dobson and Franzmann 1996 is rejected. The combination *Halovibrio denitrificans* Sorokin *et al*. 2006 is validly published with an emendation of the description of the genus *Halovibrio* Fendrich 1989 emend. Sorokin *et al*. 2006.
94	*Agrobacterium radiobacter* (Beijerinck and van Delden 1902) Conn 1942 has priority over *Agrobacterium tumefaciens* (Smith & Townsend 1907) Conn 1942 when the two are treated as members of	*Int J Syst Evol Microbiol* 2014;64:3590–3592; doi:10.1099/ijs.0.069203-0	The Judicial Commission affirms that, according to the Rules of the *International Code of Nomenclature of Bacteria* (including changes made to the wording), the combination *Agrobacterium radiobacter* (Beijerinck and van Delden 1902) Conn 1942 has priority over the combination *Agrobacterium tumefaciens* (Smith and Townsend 1907) Conn 1942 when the two are treated as members of the same species based on the principle of priority as applied to the corresponding specific epithets. The type species of the genus is *Agrobacterium tumefaciens* (Smith and Townsend 1907) Conn 1942, even if treated as a later heterotypic synonym of *Agrobacterium radiobacter* (Beijerinck and van Delden 1902) Conn 1942.

	the same species based on the principle of priority and Rule 23a Note 1 as applied to the corresponding specific epithets		*Agrobacterium tumefaciens* (Smith and Townsend 1907) Conn 1942 is typified by the strain defined on the Approved Lists of Bacterial Names and by strains known to be derived from the nomenclatural type.
95	The combinations *Lysobacter enzymogenes* subsp. enzymogenes Christensen and Cook 1978, *L. enzymogenes* subsp. *cookii* Christensen 1978 and *Streptococcus casseliflavus* (Mundt and Graham 1968) Vaughan *et al*. 1979 were in accordance with the *International Code of Nomenclature of Bacteria* at the time of publication in the *International Journal of Systematic Bacteriology*, but are not to be considered to be included on the Approved Lists of Bacterial Names	*Int J Syst Evol Microbiol* 2014;64:3920-3921; doi:10.1099/ijs.0.069211-0	The Judicial Commission affirms that, according to information presented to it, the combination *Lysobacter enzymogenes* subsp. *enzymogenes* Christensen and Cook 1978, the combination *Lysobacter enzymogenes* subsp. *cookii* Christensen 1978 and the combination *Streptococcus casseliflavus* (Mundt and Graham 1968) Vaughan *et al.* 1979 were in accordance with the wording of the 1975 and 1990 revisions of the *International Code of Nomenclature of Bacteria* but they are not to be considered to be included on the Approved Lists of Bacterial Names.
96	The properties given at the time of publication for the designated type strain of *Leifsonia rubra*	*Int J Syst Evol Microbiol* 2014;64:3593–3594; doi:10.1099/ijs.0.069229-0	The Judicial Commission affirms that, according to information presented to it, the type strain of *Leifsonia rubra* Reddy *et al.* 2003 designated in the original publication as strain CMS 76r and deposited as MTCC 4210, DSM 15304, CIP 107783 and JCM 12471 does not have properties corresponding with those of the

	Reddy *et al*. 2003, CMS 76r does not correspond with those of MTCC 4210, DSM 15304, CIP 107783 and JCM 12471 that are deposited as representing the type strain		strains held in those collections under those accession numbers. The species *Leifsonia rubra* Reddy *et al.* 2003 was not represented by an authentic deposit of a type strain at the time of effective publication in the pages of the *International Journal of Systematic and Evolutionary Microbiology*.
97	Denial of the recommendation for the conservation of the name *Streptomyces scabies*	*Int J Syst Evol Microbiol* 2020;70:1439–1440; doi:10.1099/ijsem.0.003921	The Judicial Commission denied the request for the conservation of the name *Streptomyces scabies*, ruling that the continued use of the correction *Streptomyces scabiei* is allowed.
98	The name *Bacillus aeolius* is not validly published	*Int J Syst Evol Microbiol* 2020;70:1439–1440; doi:10.1099/ijsem.0.003921	The Judicial Commission denied the request to place the name *Bacillus aeolius* on the list of rejected names. In the absence of authentic type material, the name *Bacillus aeolius* is not validly published, based on the wording of Rules 18a, 27(3) and 30(3b).
99	The name *Pectinatus portalensis* is not validly published	*Int J Syst Evol Microbiol* 2020;70:1439–1440; doi:10.1099/ijsem.0.003921	The Judicial Commission denied the request to place the name *Pectinatus portalensis* on the list of rejected names. In the absence of authentic type material, the name *Pectinatus portalensis* is not validly published, based on the wording of Rules 18a, 27(3) and 30(3b).
100	A neotype strain does not need to be designated for *Eubacterium rectale*	*Int J Syst Evol Microbiol* 2020;70:5177–5181; doi: 10.1099/ijsem.0.004390	Based on the wording of Rule 18c, the Judicial Commission denied the request for the recognition of strain A1-86 as the neotype strain of *Eubacterium rectale*, ruling that strain VPI 0990 (=ATCC 33656=CIP 105953=DSM 3377=JCM 17463=KCTC 5835=LMG 30912) is considered to be a duplicate isolate of the same strain as VPI 0989 (=ATCC 25578) and may serve as the nomenclatural type.
101	Strain ATCC 25946 (=DSM 14877) serves as the type strain of *Melittangium lichenicola* instead of ATCC 25944 (=DSM 2275)	*Int J Syst Evol Microbiol* 2020;70:5177–5181; doi: 10.1099/ijsem.0.004390	The Judicial Commission approved a request about the type strain of *M. lichenicola*, ruling: (i) that the strain deposited as ATCC 25944 (=M155=DSM 2275) does not conform with the published morphological description of *M. lichenicola*, and that this strain should not serve as the type strain because it is not an authentic representative of the designated type strain; (ii) that the reference strain Windsor M201 (=ATCC 25946=DSM 14877=NBRC 100091) should serve as the type strain of *M. lichenicola*; and (iii) that the Approved Lists of Bacterial

			Names must be corrected accordingly.
102	Strain Cc m8 (=DSM 14697=CIP 109128=JCM 12621) is an established neotype strain for the species *Myxococcus macrosporus*, replacing the designated type strain Windsor M271, and strain Mx s8 (=DSM 14675=JCM 12634) is an established neotype strain for the species *Myxococcus stipitatus*, replacing the designated type strain Windsor M78	*Int J Syst Evol Microbiol* 2020;70:5177–5181; doi: 10.1099/ijsem.0.004390	Windsor M271 and Windsor M78 are not herbarium material and hence cannot be considered preserved specimens under Rule 18a(1); *Corallococcus macrosporus* (*ex* Krzemieniewska and Krzemieniewski 1926) Reichenbach 2007 and *Myxococcus macrosporus* (Krzemieniewska and Krzemieniewski 1926) Zahler and McCurdy 1974 (Approved Lists 1980) should share the same nomenclatural type; strain Cc m8 (=DSM 14697=CIP 109128=JCM 12621) is an established neotype strain for the species *Myxococcus macrosporus*, replacing the designated type strain Windsor M271; strain Mx s8 (=DSM 14675=JCM 12634) is an established neotype strain for the species *Myxococcus stipitatus*, replacing the designated type strain Windsor M78.
103	Rejection of the name *Spirillum volutans* Ehrenberg 1832 and designation of *Spirillum winogradskyi* as the type species of the genus *Spirillum*	*Int J Syst Evol Microbiol* 2022;72:005197; doi: 10.1099/ijsem.0.005197	Based on the description of *Spirillum volutans* cited in the Approved Lists, the Judicial Commission concluded that it might be possible to locate a neotype strain, through either re-isolation or searching in culture collections. Strain ATCC 19553 is a good candidate. Therefore, the Judicial Commission did not place the name *Spirillum volutans* Ehrenberg 1832 (Approved Lists 1980) on the list of rejected names.
104	Rejection of the name *Beijerinckia fluminensis* Döbereiner and Ruschel 1958	*Int J Syst Evol Microbiol* 2022;72:005197; doi: 10.1099/ijsem.0.005197	Isolation of strains that correspond to the properties of *Beijerinckia fluminensis* was reported from different countries. The Judicial Commission therefore did not place the name *Beijerinckia fluminensis* Ehrenberg 1832 (Approved Lists 1980) Döbereiner and Ruschel 1958 (Approved Lists 1980) on the list of rejected names at this time, as a possible candidate neotype strain may already exist.
105	Renaming the genus *Rhodoligotrophos* as *Rhodoligotrophus*	*Int J Syst Evol Microbiol* 2022;72:005197; doi: 10.1099/ijsem.0.005197	The Judicial Commission concluded that *Rhodoligotrophos* Fukuda *et al.* 2012 does not violate the rules of the ICNP. The Judicial Commission should decide on orthographical corrections from case to case. In the case of *Rhodoligotrophos*, the request was denied.
106	Conservation of the	*Int J Syst Evol Microbiol*	The Judicial Commission placed the epithet *hoagii* in *Corynebacterium hoagii*

	name *Rhodococcus equi* and rejection of its earlier heterotypic synonym *Corynebacterium hoagii*	2022;72:005197; doi: 10.1099/ijsem.0.005197	(Morse 1912) Eberson 1918 (Approved Lists 1980) and *Rhodococcus hoagii* (Morse 1912) Kämpfer *et al.* 2014 on the list of *epitheta specifica et subspecifica rejicienda*. The request to conserve the epithet *equi* in *Rhodococcus equi* (Magnusson 1923) Goodfellow and Alderson 1977 (Approved Lists 1980) was denied.
107	Rejection of the name *Thermomicrobium fosteri* Phillips and Perry 1976 (Approved Lists 1980)	*Int J Syst Evol Microbiol* 2022;72:005197; doi: 10.1099/ijsem.0.005197	Under the assumption that *Thermomicrobium fosteri* Phillips and Perry 1976 (Approved Lists 1980) is based on a mixed culture, the Judicial Commission rejected the name as a *nomen confusum* according to Rule 56a (3) and a *nomen dubium* according to Rule 56a (2).
108	Rejection of the name *Hyphomonas rosenbergii* Weiner *et al.* 2000	*Int J Syst Evol Microbiol* 2022;72:005197; doi: 10.1099/ijsem.0.005197	The deposited strains ATCC 43869^T and DSM 17769^T apparently do not belong of the genus *Hyphomonas*, but most likely belong to the genus *Henriciella*. However, the 16S rRNA gene sequence with accession number AF082795 affiliates with species of *Hyphomonas*. One possible interpretation of the data is that AF082795 was derived from VP6^T but ATCC 43869^T and DSM 17769^T are not deposits of VP6^T. The second possibility is that AF082795 is not derived from VP6^T but ATCC 43869^T and DSM 17769^T are deposits of VP6^T. The third possibility is that VP6^T was indeed a mixed culture and for this reason AF082795 as well as ATCC 43869^T (= DSM 17769^T) were both derived from it. As it was not possible to distinguish between the three scenarios, the Judicial Commission did not take action, and the request to place *Hyphomonas rosenbergii* Weiner *et al.* 2000 on the list of rejected names was denied.
109	Rejection of the names *Bacillus aerius* Shivaji *et al.* 2006, *Bacillus aerophilus* Shivaji *et al.* 2006 and *Bacillus stratosphericus* Shivaji *et al.* 2006 because type strains are not available	*Int J Syst Evol Microbiol* 2022;72:005197; doi: 10.1099/ijsem.0.005197	The Judicial Commission concluded that the names *Bacillus aerius* Shivaji *et al.* 2006, *Bacillus aerophilus* Shivaji *et al.* 2006 and *Bacillus stratosphericus* Shivaji *et al.* 2006 are not validly published although they were proposed in an effective publication in the IJSEM. In particular, the three names did not meet the requirements listed in Rule 30 (3b) and Rule 30 (4). Having an effective publication in the IJSEM is neither a necessary nor a sufficient condition for a name to be validly published.
110	Rejection of the name *Actinobaculum massiliense* Greub and	*Int J Syst Evol Microbiol* 2022;72:005197; doi: 10.1099/ijsem.0.005197	The Judicial Commission concluded that the name *Actinobaculum massiliense* corrig. Greub and Raoult 2006 is not validly published, despite its inclusion in Validation List No. 111, because the requirements for valid publication, specifically

	Raoult 2006		Rules 18a, 27 (3) and 30 (3b), were not met.
111	Conservation of the name *Methanocorpusculum parvum*	*Int J Syst Evol Microbiol* 2022;72:005197; doi: 10.1099/ijsem.0.005197	The Judicial Commission concluded that the name *Methanocorpusculum parvum* Zellner *et al.* 1988 does not become illegitimate by considering it as a later heterotypic synonym of *Methanogenium aggregans* Ollivier *et al.* 1985 ≡ *Methanocorpusculum aggregans* (Ollivier *et al.* 1985) Xun *et al.* 1989. It would indeed violate the *Code* to treat *Methanogenium aggregans* Ollivier *et al.* 1985 as the correct name of a species that contains both the nomenclatural type of *Methanogenium aggregans* Ollivier *et al.* 1985 and *Methanogenium aggregans* Ollivier *et al.* 1985 ≡ *Methanocorpusculum aggregans* (Ollivier *et al.* 1985) Xun *et al.* 1989. Yet this does not render *Methanogenium aggregans* Ollivier *et al.* 1985 an illegitimate name. The status of *Methanocorpusculum parvum* Zellner *et al.* 1988 as the nomenclatural type of *Methanocorpusculum* Zellner *et al.* 1988 is thus not in danger.
112	Rejection of the name *Seliberia* Aristovskaya and Parinkina 1963 (Approved Lists 1980)	*Int J Syst Evol Microbiol* 2022;72:005481; doi:10.1099/ijsem.0.005481	The request to place *Seliberia* Aristovskaya and Parinkina 1963 (Approved Lists 1980) on the list of rejected names is denied because the information provided is insufficient for drawing a conclusion.
113	Rejection of the name *Shewanella irciniae* Lee *et al.* 2006	*Int J Syst Evol Microbiol* 2022;72:005481; doi:10.1099/ijsem.0.005481	The request to place *Shewanella irciniae* Lee *et al.* 2006 on the list of rejected names is denied because the information provided is insufficient for drawing a conclusion.
114	Rejection of the name *Enterobacter siamensis* Khunthongpan *et al.* 2014	*Int J Syst Evol Microbiol* 2022;72:005481; doi:10.1099/ijsem.0.005481	The request to place *Enterobacter siamensis* Khunthongpan *et al.* 2014 on the list of rejected names is denied because the information provided is insufficient for drawing a conclusion.
115	Rejection of the name *Moorella thermoautotrophica* (Wiegel *et al.* 1981) Collins *et al.* 1994	*Int J Syst Evol Microbiol* 2022;72:005481; doi:10.1099/ijsem.0.005481	The epithet in *Moorella thermoautotrophica* (Wiegel *et al.* 1981) Collins *et al.* 1994 is placed on the list of rejected epithets because this species name is a *nomen confusum*.
116	Assessment of the consequences of Rule 8 being retroactive	*Int J Syst Evol Microbiol* 2022;72: 005481;doi:10.1099/ijsem.0.005481	The Judicial Commission revisits the names of taxa above the rank of genus which should be comprised of the stem of the name of a nomenclatural type and a category-specific ending but fail to do so. Such names should be orthographically corrected if the sole error is the inadvertent usage of an incorrect stem, and be

			regarded as illegitimate if otherwise. The necessary corrections are made for a number of names. Class names such as *Clostridia* have an actual ending of -a instead of -ia and are illegitimate as long as Rule 8 is retroactive.
117	Designation of *Methylothermus subterraneus* Hirayama *et al.* 2011 as the type species of the genus *Methylothermus*	*Int J Syst Evol Microbiol* 2022;72:005481; doi:10.1099/ijsem.0.005481	The request to designate *Methylothermus subterraneus* Hirayama *et al.* 2011 as the type species of the genus *Methylothermus* is denied because an equivalent action compatible with the *Code* was already conducted.
118	Orthographical correction of the name *Flaviaesturariibacter* to *Flavaestuariibacter*	*Int J Syst Evol Microbiol* 2022;72:005481; doi:10.1099/ijsem.0.005481	The possible orthographical correction of the name *Flaviaesturariibacter* is treated, as are the analogous cases of *Fredinandcohnia* and *Hydrogeniiclostidium*. The genus names are corrected to *Flaviaestuariibacter*, *Ferdinandcohnia* and *Hydrogeniiclostridium*, respectively.
119	Assignment of *Actinomycetales* Buchanan 1917 (Approved Lists 1980) as nomenclatural type of the class *Actinobacteria* Stackebrandt *et al.* 1997	*Int J Syst Evol Microbiol* 2022;72:005481; doi:10.1099/ijsem.0.005481	It is concluded that assigning *Actinomycetales* Buchanan 1917 (Approved Lists 1980) as nomenclatural type of the class *Actinobacteria* Stackebrandt *et al.* 1997 would not render this name legitimate if Rule 8 remained retroactive. The request is granted but *Actinomycetales* is also assigned as type of *Actinomycetes* Krassilnikov 1949 (Approved Lists 1980). This means that *Actinomycetia* Salam *et al.* 2020 would become illegitimate if Rule 8 was made non-retroactive and the correct name of the class would then be *Actinomycetes* Krassilnikov 1949 (Approved Lists 1980).
120	Orthographical correction of the name *Amycolatopsis albidoflavus* Lee and Hah 2001 to *Amycolatopsis albidiflava* corrig. Lee and Hah 2001	*Int J Syst Evol Microbiol* 2022;72:005481; doi:10.1099/ijsem.0.005481	The possible orthographical correction of the name *Amycolatopsis albidoflavus* is treated. It is grammatically corrected to *Amycolatopsis albidoflava*. Six names that could, according to Rule 61, be grammatically corrected by anyone are also corrected.
121	Revision of Judicial Opinion 69	*Int J Syst Evol Microbiol* 2022;72:005481; doi:10.1099/ijsem.0.005481	The request to revise Opinion 69 is denied because there is no basis in the *Code* for revoking the rejection of a name or epithet or revoking the conservation of a name or epithet. However, it is also noted that Opinion 69 does not have the undesirable consequences emphasized in the request.
122	Rejection of various	*Int J Syst Evol Microbiol*	The request to reject various taxon names of *Mollicutes* proposed in 2018 is

	taxon names of *Mollicutes* validly published in 2018	2022;72:005481; doi:10.1099/ijsem.0.005481	denied because it is based on misinterpretations of the *Code*, which are clarified. In particular, the *Code* guarantees taxonomic freedom. Alternative ways to solve the perceived problems are outlined.
123	Correction of the type strain designations for *Aeromonas punctata* and *Aeromonas punctata* subsp. *punctata* from ATCC 15468 to NCMB 74 , and clarification of the best way to write author citations for several other *Aeromonas* names	*Int J Syst Evol Microbiol* 2022;72:005708; doi:10.1099/ijsem.0.005708	The epithet of the name *Aeromonas punctata* is placed on the list of rejected epithets, and the citation of authors of selected names within the genus *Aeromonas* is clarified.
124	Rejection of the genus name *Borreliella* Adeolu and Gupta 2015 and the names of all of its species	*Int J Syst Evol Microbiol* 2022;72:005708; doi:10.1099/ijsem.0.005708	The request to place the name *Borreliella* on the list of rejected names because the request is based on a misinterpretation of the Rules of the ICNP.
125	Rejection of the name *Lactobacillus fornicalis*	*Int J Syst Evol Microbiol* 2022;72:005708; doi:10.1099/ijsem.0.005708	The request to place the name *Lactobacillus fornicalis* on the list of rejected names is denied because the provided information does not yield a reason for rejection.
126	Rejection of the names *Prolinoborus* Pot *et al.* 1992 and *Prolinoborus fasciculus* (Srength *et al.* 1976) Pot *et al.* 1992	*Int J Syst Evol Microbiol* 2022;72:005708; doi:10.1099/ijsem.0.005708	The request to place the names *Prolinoborus* and *Prolinoborus fasciculus* on the list of rejected names is denied because a relevant type strain deposit was not examined.
127	Correction of the type strain of *Agrobacterium tumefaciens* (Smith and Townsend 1907) Conn 1942 (Approved Lists 1980) from ATCC 23308	*Int J Syst Evol Microbiol* 2022;72:005708; doi:10.1099/ijsem.0.005708	The strain deposited as ATCC 4720 is assigned as the type strain of *Agrobacterium tumefaciens*, thereby correcting the Approved Lists.

	to ATCC 4720		
128	The Judicial Commission is asked to confirm that the common ending –*proteobacteria* of the names of the classes *Alphaproteobacteria* Garrity *et al*. 2006, *Betaproteobacteria* Garrity *et al*. 2006, *Deltaproteobacteria* Kuever *et al*. 2006, *Epsilonproteobacteria* Garrity *et al*. 2006 and *Gammaproteobacteria* Garrity et al. 2005 is not to be treated as being indicative of the taxonomic or phylogenetic placement of these classes.	*Int J Syst Evol Microbiol* 2023;73:005797; doi:10.1099/ijsem.0.005797	It is confirmed that the common ending –*proteobacteria* of some class names is not indicative of a joint taxonomic or phylogenetic placement.
	The Judicial Commission is asked to confirm that the nomenclatural type of *Mollicutes* Edward and Freundt 1967 (Approved Lists 1980) is *Mycoplasmatales* Freundt 1955 (Approved Lists 1980).		It is confirmed that the nomenclatural type of *Mollicutes* Edward and Freundt 1967 (Approved Lists 1980) is *Mycoplasmatales* Freundt 1955 (Approved Lists 1980).
	The Judicial Commission is asked to confirm that the ICNP does not indicate that the placement		It is confirmed that the placement of a name on the list of rejected names does not imply that another name with the same spelling but a distinct rank is also placed on that list.

	of a name at a certain rank on the list of rejected names implies the placement of a name at another rank on the list of rejected names merely because the two names have an identical spelling.		
	The Judicial Commission is asked to place the names at the rank of class *Bacteria* Haeckel 1894 (Approved Lists 1980), *Microtatobiotes* Philip 1956 (Approved Lists 1980), *Proteobacteria* Stackebrandt *et al*. 1988 and *Schizomycetes* Nägeli 1857 (Approved Lists 1980) on the list of rejected names as *nomina dubia et ambigua*.		The Judicial Commission has placed the names *Bacteria* Haeckel 1894 (Approved Lists 1980), *Microtatobiotes* Philip 1956 (Approved Lists 1980), *Proteobacteria* Stackebrandt *et al*. 1988 and *Schizomycetes* Nägeli 1857 (Approved Lists 1980) on the list of *nomina rejicienda*
	The Judicial Commission is asked to place the names at the rank of class *Photobacteria* Gibbons and Murray 1978 (Approved Lists 1980) and *Scotobacteria* Gibbons and Murray 1978 (Approved Lists 1980) as well as *Anoxyphotobac*-		The Judicial Commission has placed the names *Photobacteria* Gibbons and Murray 1978 (Approved Lists 1980), *Scotobacteria* Gibbons and Murray 1978 (Approved Lists 1980), *Anoxyphotobacteria* (Gibbons and Murray 1978) Murray 1988, *Archaeobacteria* Murray 1988, *Firmibacteria* Murray 1988 and *Oxyphotobacteria* (*ex* Gibbons and Murray 1978) Murray 1988 on the list of *nomina rejicienda*.

	teria (Gibbons and Murray 1978) Murray 1988, *Archaeobacteria* Murray 1988, *Firmibacteria* Murray 1988 and *Oxyphotobacteria* (*ex* Gibbons and Murray 1978) Murray 1988 on the list of rejected names, using the rejection of names conducted in the Supplementary Information to Opinion 79 as precedent.		
	The Judicial Commission is asked to define the nominative plural of N.L. masc. n. *bacter* as *bacteres*.		For the nominative singular suffix of genus names -*bacter* the genitive singular and nominative plural forms –*bacteris* and -*bacteres* are confirmed.
	The Judicial Commission is asked to orthographically correct *Gemmatimonadetes* Zhang *et al*. 2003 to *Gemmatimonades* corrig. Zhang *et al*. 2003 (old style) or *Gemmatimonadia* corrig. Zhang *et al*. 2003 (new style).		The class name *Gemmatimonadetes* Zhang *et al*. 2003 was orthographically corrected to *Gemmatimonadia* corrig. Zhang *et al*. 2003.
	The Judicial Commission is asked to orthographically correct *Chrysiogenetes* Garrity and Holt 2002 to *Chrysiogenes*		The class name *Chrysiogenetes* Garrity and Holt 2002 was orthographically corrected to *Chrysiogenia* corrig. Garrity and Holt 2002. For the nominative singular suffixes of genus names -*genes*, the genitive singular and nominative plural forms -*genis* and -*genes* are confirmed).

	corrig. Garrity and Holt 2002 (old style) or *Chrysiogenia* corrig. Garrity and Holt 2002 (new style).		
	The Judicial Commission is asked to orthographically correct *Aquificae* Reysenbach 2002 to *Aquifices* corrig. Reysenbach 2002 (old style), thereby confirming that the nominative plural of *–fex* is *-fices*, or to *Aquificia* corrig. Reysenbach 2002 (new style).		The class names *Aquificae* Reysenbach 2002 was orthographically corrected to *Aquificia* corrig. Reysenbach 2002. For the nominative singular suffix of genus names *-fex*, the genitive singular and nominative plural forms *-ficis* and *-fices* are confirmed.
	The Judicial Commission is asked to orthographically correct *Verrucomicrobiae* Hedlund *et al*. 1998 to *Verrucomicrobia* corrig. Hedlund *et al*. 1998 and *Opitutae* Choo *et al*. 2007 to *Opituti* corrig. Choo *et al*. 2007 (old style) or alternatively to orthographically correct *Verrucomicrobiae* Hedlund *et al*. 1998 to *Verrucomicrobiia* corrig. Hedlund *et al*. 1998 and *Opitutae* Choo *et al*. 2007 to *Opitutia* corrig.		The class names *Verrucomicrobiae* Hedlund *et al*. 1998 and *Opitutae* Choo *et al*. 2007 were orthographically corrected to *Verrucomicrobiia* corrig. Hedlund *et al*. 1998 and *Opitutia* corrig. Choo *et al*. 2007, respectively.

	Choo *et al*. 2007 (new style).		
129	Has the name *Firmicutes* has been validly published and has gained standing in view of its inclusion in the Approved Lists; (2) does the Judicial Commission have the power to conserve the name *Firmicutes*; (3) is the Judicial Commission is willing to decision to retain the name *Firmicutes* as an exception to the recently proposed naming scheme for phyla.	*Int J Syst Evol Microbiol* 2024;74:006064; doi:10.1099/ijsem.0.006064	The name *Firmicutes* has the category ‘division’ and was included in the Approved Lists of Bacterial Names, although that category had previously been removed from the International Code of Nomenclature of Bacteria (1975 revision onwards). When the category ‘phylum’ was introduced into the International Code of Nomenclature of Prokaryotes (ICNP) in 2021, equivalence between ‘phylum’ and ‘division’ was not stipulated. The inclusion of *Firmicutes* corrig. Gibbons and Murray 1978 in the Approved Lists was an error. The name is either not validly published or illegitimate because its category is not covered by the ICNP. Since *Firmicutes* corrig. Gibbons and Murray 1978 is also part of a ‘misfitting megaclassification’ recognized in Opinion 128, the name is rejected. *Gracilicutes* Gibbons and Murray 1978 (Approved Lists 1980) and *Anoxyphotobacteriae* Gibbons and Murray 1978 (Approved Lists 1980) are also rejected.
130	Is the bacterial genus name *Rhodococcus* Zopf 1891 illegitimate?	*Int J Syst Evol Microbiol* 2024;74:006414; doi:10.1099/ijsem.0.006414	The name *Rhodococcus* Zopf 1891 (Approved Lists 1980) is significantly more important than the name *Rhodococcus* Hansgirg 1884 and therefore the former is conserved over the latter. This makes the name *Rhodococcus* Zopf 1891 (Approved Lists 1980) legitimate.
131	Conservation of the illegitimate prokaryotic generic name *Proteus* Hauser 1885 (Approved Lists 1980)	*Int J Syst Evol Microbiol* 2024??;74???:xxxxx; doi:10.1099/ijsem.0.00xxxx	XXX PENDING

APPENDIX 6. PUBLISHED SOURCES FOR RECOMMENDED MINIMAL STANDARDS FOR THE DESCRIPTION OF NEW TAXA OF PROKARYOTES

Recommendations for minimal standards of description have been published in the IJSEM for the following groups. This list is current through September 2024 [TO BE UPDATED FURTHER IF RELEVANT]

**Group**	**References**
General (genome sequences)	[64]
Aerobic, endospore-forming bacteria	[65]
Anoxygenic phototrophic bacteria	[66]
Bifidobacterium, *Lactobacillus* and related genera	[67]
Brucella	[68, 69]
Campylobacteraceae	[70, 71]
Flavobacteriaceae	[72]
Halobacteria	[73]
Halomonadaceae	[74, 75]
Helicobacter and *Helicobacteraceae*	[76]
Methanogenic Archaea	[77]
Micrococcineae	[78]
Mollicutes	[79–81]
Moraxella and *Acinetobacter*	[82]
Mycobacterium	[83]
Mycoplasmatales	[84] (superseded by recommendations on *Mollicutes* above)
Pasteurellaceae	[85]
Rhizobia and Agrobacteria	[86]
Root- and Stem-Nodulating Bacteria	[87]
Staphylococcus	[88]
Xanthomonas	[89]

APPENDIX 7. PUBLICATION OF A NEW NAME

Valid publication of the name of a taxon (including a new combination) requires publication in the *International Journal of Systematic and Evolutionary Microbiology* (IJSEM) of (a) the name of the taxon, (b) a designation of a type for the new taxon, and (c) a description or a reference to an effectively published description of the taxon, whether in the *IJSEM* or in another publication.

(1) The new name must be in the correct form. Generic and suprageneric names are single words in Latin form and spelled with an initial capital letter. Names of species are binary combinations in Latin form consisting of a generic name and a single, specific epithet; the latter spelled with an initial lowercase letter. Subspecific names are ternary combinations consisting of the name of a species followed by the term “subspecies” (abbreviation: “subsp.”) and this followed by a single subspecific epithet. Names of taxa from the rank of order through tribe are formed by the addition of the appropriate suffix to the stem of the name of the type genus (see (5) below). The suffix for order is -*ales*, for suborder -*ineae*, for family -*aceae*, and for tribe -*eae*. The suffix for class is -*ia*, for subclass –*idae*. These endings are added to the stem of the name of the type genus of the type genus of the class or sub-class. Names of new phyla are formed by the addition of the suffix –*ota* to the stem of the name of one of the contained genera. Names of new kingdoms are formed by the addition of the suffix –*ati* to the stem of the name of one of the contained genera. Names of new do-mains are formed as plural of the last component of the name of one of the contained gen-era.

Whenever possible, the title of the paper should include any new names or combinations that are proposed in the text.

(2) New names are proposed by appending the phrase “*species nova*” (abbreviation: sp. nov.), “*genus novum*” (abbreviation: gen. nov.), “*combinatio nova*” (abbreviation: comb. nov.), or the like after the name or combination that is being proposed. Revival of names published prior to 1 January 1980 but not included in an Approved List may be effected by provisions in Rule 33.

A list of abbreviations used in the description of new taxa is given in the following Table.

Common abbreviations used in publications of names of new taxa of prokaryotes and their etymologies.

(modified from [90])

	Abbreviation	Full spelling	Explanation	ICNP rule
**Taxonomic ranks**	subsp. nov.	*subspecies nova*	New subspecies	13a
	sp. nov.	*species nova*	New species	27, 33a
	gen. nov.[1]	*genus novum*	New genus	27, 33a
	fam. nov.	*familia nova*	New family	27
	ord. nov.	*ordo novus*	New order	33a
	class. nov.	*classis nova*	New class	33a
	phyl. nov.	*phylum novum*	New phylum	33a
	regn. nov.	*regnum novum*	New kingdom	33a
	dom. nov.	*dominium novum*	New domain	33a
	comb. nov.	*combinatio nova*	New combination, when an established epithet (taken from the basonym) is combined with another genus name to form a species name, or with another genus name and another epithet to form a subspecies name	27, 33a, 34a
	nom. nov.[1]	*nomen novum*	A new name to be established when the establishment of a comb. nov. would lead to a homonym	34a
	nom. rev.[1]	*nomen revictum*	Reserved for names that existed before 1980, were not included in the Approved Lists of 1980 and are revived	28a, 33c
	nom. approb.[1]	*nomen approbatum*	Name included in an Approved List	33b
**Categories of words and word elements**	n.	noun		
	v.	verb		
	adj.	adjective		

	part.	participle		
	pres. part.	present participle		
	part. adj.	participle used as adjective	To comply with Rule 12c(1) so that a participle can be used as a specific or subspecific epithet	
	prep.	preposition		
	pref.	prefix		
	pron.	pronoun		
	suff.	suffix		
**Terms referring to gender and grammatical declensions**	masc.	masculine		
	fem.	feminine		
	neut.	neuter		
	sing.	singular		
	pl.	plural		
	nom.[1]	nominative		
	gen.[1]	genitive		
	dim.	diminutive		
**Source of words or word elements**	L.	Latin	Reserved for words used in classical Latin	
	N.L.	Neo-Latin	Words newly coined, based on classical Latin elements and/or Latinized modern words	
	M.L.	Medieval Latin	Seldom used; in the past M.L. was often used for Modern Latin, now to be replaced with N.L.	Recommendation 6(6)
	Gr.	Greek		
**Other relevant abbreviations**	corrig.	*corrigendum*	Indicates a corrected typographical or orthographic error	61
	emend.	*emendavit*	Alteration of the diagnostic characters or of the circumscription of a taxon	35

[1] The abbreviations “nom.” and “gen.” can thus mean nomen and nominative and genus or genitive, respectively, depending on the context.

(3) The name must not be a later homonym of a name previously validly published in the botanical and zoological literature (See Appendix 3 for published sources of names of plant and animal taxa and Rule 51b for exceptions.)

(4) Rule 27(2)b states that the derivation (etymology) of a new name (and, if necessary, of a new combination) must be given. It is recommended to present the etymology, preceded by the proposed syllabification, in the style shown in the following ~~hypothetical~~ example from the (corrected) proposal of the new genus name *Thermalbibacter* Zhao *et al*. 2023: *Thermalbibacter* gen. nov. (Therm.al.bi.bac'ter, Gr. masc. adj. *thermos*, hot; L. masc. adj. *albus*, white; N.L. masc. n. *bacter*, a rod; N.L. masc. n. *Thermalbibacter*, a white rod from a hot environment).

The syllabification is printed in Roman type, the stressed syllable is followed by the apostrophe sign (‘), and the last syllable is followed by a full stop. For guidelines on how to break names into syllables, see [91].

(5) The name must be accompanied by a description of the taxon or by a reference to an effectively published description of the taxon (see (7) below).

(6) The nomenclatural type of a new taxon must be designated. In the case of species and subspecies, the type strain must be designated by the author’s strain number as well as the accession number under which it is held by at least two culture collections located in different countries from which cultures of the strain are available without restrictions.

A nomenclatural type is that constituent element of a taxon to which the name of a taxon is permanently attached. The type of a species or a subspecies is a strain, that of a genus or a subgenus is a species, and that of a domain, kingdom, phylum, class, subclass, order, suborder, family, or tribe is a genus. With few exceptions (provided for by Rule 8 and Rule 21b) the name of the higher taxon is based on the name of that genus.

A type strain is one of the strains on which the authors who first described a named species or subspecies based the description of the species or subspecies, and which the authors or subsequent authors designated as a type.

A neotype strain replaces a type strain which can no longer be found (Rule 18c) or is no longer viable (Rule 18a(2), Rule 30(3)). The neotype must possess the characteristics as given in the original description; any deviations must be explained. A neotype strain must be proposed by an author in the IJSEM (proposed neotype) together with a reference (or references) to the first description and name for the microorganism (or to an Approved List, if appropriate), a description (or reference to a description) of the proposed neotype strain, and a record of the designation of the author(s) for the type strain and at least two culture collections from which cultures of the strain are available. The neotype strain becomes established two years after the date of publication in the IJSEM (established neotype). Any objections must be referred to the Judicial Commission within the first year after publication of the proposal. A neotype strain shall be proposed only after a careful search for original strains. If an original strain is subsequently discovered, the matter shall be referred immediately to the Judicial Commission. Allowance is made for replacement of an unsuitable type strain.

(7) Descriptions of taxa must include the following information: (a) those characteristics, which are essential for membership in the taxon, i.e., those characteristics that constitute

the basic concept of the taxon; (b) those characteristics that qualify the taxon for membership in the next higher taxon; (c) the diagnostic characteristics, i.e., those characteristics that distinguish the taxon from closely related taxa; and (d) in the case of species, the total number of strains studied, and the strain designations must be given. From this information, the detailed results for each strain can be reconstructed without the full publication of the details for each strain. When appropriate, suitable photomicrographs and, if necessary, electron photomicrographs should be included as part of the description, to show morphological or anatomical characters that are pertinent to the classification. Descriptions should conform, at least, to such proposed minimal standards for the description of new taxa in certain groups as have been approved by the ICSP Subcommittees on Taxonomy.

APPENDIX 8. PREPARATION OF A REQUEST FOR AN OPINION

In cases wherein strict adherence to the rules of nomenclature would produce confusion or would not result in nomenclatural stability, exceptions to the rules may be requested of the Judicial Commission of the ICSP. Requests for Opinions must be accompanied by a comprehensively documented statement of the relevant facts. The Judicial Commission will consider all Requests for Opinions and must issue an Opinion in the IJSEM whether or not the proposal is granted or denied. The title of a manuscript should provide a concise statement of the contents of the manuscript. If an opinion of the Judicial Commission is requested, “Request for an Opinion” should appear as a subtitle. The Judicial Commission recently published detailed guidelines to assist potential authors in deciding whether their concern should be the subject of a Request, and if so, in composing it with the greatest chance of success [92]. A Request for an Opinion submitted in an acceptable form, as determined by peer review, will be published in the IJSEM. If a request is not supported by adequate evidence, it will be returned to the author for revision. When an Opinion is challenged, the basis of the challenge must be stated and supported by a documented statement of the relevant facts. Requests for Opinions will be considered by the Judicial Commission within 6 months. Further information is found in Article 8 of the Statutes of the International Committee on Systematics of Prokaryotes.

APPENDIX 9. ADVICE ON THE FORMATION OF NAMES AND ORTHOGRAPHY

Note: This appendix is adapted from [93].

A. Formation of Compound Names

(1) Compound names are formed by combining two or more words or word elements, generally of Latin and/or Classical Greek origin, into one generic name or specific epithet. In most cases, two word elements are used (e.g., *Thio/bacillus*, *thio/philus*) although, as many as four elements may be found (e.g., *Ecto/thio/rhodo/spira*). A name or epithet that combines elements derived from two or more Greek or Latin words should be formed, as far as practicable, in accordance with classical usage. The combination of word elements follows four basic rules:

(a) The word stems are used, except for the last word element.

(b) For compound names that contain a noun or adjective in a non-final position, the connecting vowel is -i- if the preceding word element is of Latin origin; -o- if the preceding word element is of Greek origin. Greek is more flexible than Latin about the connecting vowel, and other connecting vowels than -o- may be used if a precedent is found in Greek.

Example: *Corynebacterium*.

Compound specific or subspecific epithets of prokaryotes based on localities can be formed by concatenating the genitives of the components, if the name of the locality lends itself to translation into Latin. In such names, the basic noun comes first and is followed by the descriptive word, which can be an adjective or a noun.

Examples for a noun followed by an adjective: *aquaemixtae*, *lacussalsi*, *marisnigri*, for two nouns: *vallismortis*, *lacuslunae*.

Binomial names of plants or animals can be treated in a similar way.

Example: *Sphingomonas bovisgrunnientis*.

(c) The connecting vowel is dropped when the following word element starts with a vowel. The connecting vowel may be dropped when the preceding word element ends in the same vowel.

(d) Hyphens and diacritic signs are not allowed (see Rules 12a and 64, respectively).

(2) Exemptions exist only for the following cases:

(a) When well-established word elements from chemistry or physics are used, their use in these sciences should be followed.

Examples: *thio-* for sulfur does not lose the -o- in combinations such as *Thioalkalibacter* and *thiooxidans* (following the usage in chemistry: thioether, thioester); likewise *radio-* would not lose the -o- in combinations such as ‘*Radioalkalibacter*’ or ‘*radioegens*’ (following the usage in physics: radioactive).

(b) As in inorganic chemistry, the vowels -i and -o are used to indicate different oxidation levels of cations (e.g. ferri, ferro, cupri, cupro, etc.), they do not fall under the Greek/Latin rules for connection vowels when used in prokaryote names.

Examples: *Ferrimonas* is an Fe^{3+} reducer, while *Ferroglobus* is an Fe^{2+} oxidizer.

(c) In word components such as allo-, bio-, geo-, halo-, hetero-, iso-, meso-, neo-, macro-, micro-, etc., the connecting vowel -o- may be retained when a component follows that begins with a vowel (for reasons of clarity or of previous usage).

(d) Greek prepositions and prefixes are not followed by a connecting vowel.

Examples: *Metakosakonia*, *Paracoccus*.

When Greek prepositions and prefixes that end in a vowel (e.g., epi, kata, meta, para) are attached to word elements that begin with a vowel, the final vowel is elided.

Examples: *Eperythrozoon*, *Paralcaligenes*, *Parendozoicomonas*, *Vibrio metoecus*.

Exceptions are the prepositions peri and pro, which do not elide.

Example: *Fusobacterium periodonticum*.

Prepositions formed from Greek adjectives (e.g., poly, mega) and adverbs (e.g., exo and eu) also do not elide.

Examples: *Polyangium*, *Clostridium polyendosporum*.

(e) Latin prepositions and prefixes are not followed by a connecting vowel. When Latin prepositions and prefixes that end in a vowel are attached to word elements that begin with a vowel, the final vowel is not elided, conforming to the usage in classical Latin.

(f) Adverbs are rarely used in compound words and more extensive use is not encouraged. For Latin adverbs, the connecting vowel -i- may be used; it is dropped if the following word element starts with a vowel.

Examples: *Paenibacillus*, *Paenalcaligenes*.

B. Generic (and Subgeneric) Names

(1) The name of a genus (or subgenus) is a Latin noun in the nominative case. If adjectives or participles are chosen to form generic names, they must be transformed into nouns and handled as such. In some cases, this process has already happened in classical Latin (e.g., *Serpens*).

Examples: (i) genuine nouns: *Bacillus*, *Streptococcus*, *Escherichia*, *Azotobacter*; (ii) adjectives used as nouns: *Dermatophilus*, *Halorubrum*, *Methanosalsum*, *Rubritepida*; (iii) participles of the present used as nouns: *Agarivorans*, *Myceligenerans*, *Serpens*; (iv) participles of the perfect used as nouns: *Amycolata*, *Aquiflexum*, *Gemmata*, *Microlunatus*, *Pectinatus*.

(2) Both Latin and Greek have three genders, i.e., contain nouns of masculine, feminine and neuter gender. Adjectives associated with nouns follow these in gender. For the correct formation of specific epithets (as adjectives) it is, therefore, necessary to know the gender of the genus name.

Examples for some last components in compound generic names are:

(i) of masculine gender: -*arcus*, -*bacillus*, -*bacter*, -*coccus*, -*ger*, -*globus*, -*myces*, -*philus*, -*planes*, -*sinus* and -*vibrio*;

(ii) of feminine gender: -*arcula*, -*cystis*, -*ella*, -*ia*, -*illa*, -*ina*, -*musa*, -*monas*, -*opsis*, -*phaga*, -*pila*, -*rhabdus*, -*sarcina*, -*sphaera*, -*spira*, -*spina*, -*spora*, -*thrix* and -*toga*;

(iii) of feminine or masculine gender: -*cola* (-*incola*);

(iv) of neuter gender: -*bacterium*, -*bactrum*, -*baculum*, -*filamentum*, -*filum*, -*genium*, -*microbium*, -*nema*, -*plasma*, -*spirillum*, -*sporangium* and -*tomaculum*;

(v) of masculine or feminine or neuter gender: -*ferax*, -*fex* and -*vorax*.

Names ending in –*oides* are formed by adding that suffix to the stem of the preceding word or word element and have the neuter gender. Names ending in –*opsis* (from Gr. fem. n. *opsis* aspect, appearance) must be treated as feminine. However, generic names ending in –*oides* or –*opsis* assigned to different genders by the authors cannot necessarily be corrected retroactively.

Examples: *Bacteroides* and *Nocardioides* are masculine.

(3) The gender of a new genus name should be given in the etymology.

C. Specific (and Subspecific) Epithets

(1) Rule 12c of the Code demands that specific (or subspecific) epithets must be treated in one of three following ways:

(a) as an adjective that must agree in gender with the generic name;

(b) as noun in apposition in the nominative case;

(c) as a noun in the genitive case.

Examples: (a) *Staphylococcus aureus* (adjective: 'golden'); (b) *Desulfovibrio gigas* (nominative noun: 'the giant'); (c) *Escherichia coli* (genitive noun: 'of the *colum*=colon').

(2) *Adjectives and participles as specific epithets*

(a) Latin adjectives belong to the 1st, 2nd or 3rd declension. Those of the 1st and 2nd declension have different endings in the three genders. For adjectives in the 3rd declension, the situation is more complicated, as some adjectives don't change with gender, some that do change with gender, and some that are identical in the masculine and feminine gender and different in the neuter.

Table 1 gives some examples. Note that comparative adjectives are also listed. It is recommended always to look up an adjective in a dictionary before using it for the formation of a name.

Table 1. *Examples of Latin adjectives*

**Masculine**	**Feminine**	**Neuter**	**English translation**
1st and 2nd declension			
*bonus**	*bona*	*bonum*	good
*aureus**	*aurea*	*aureum*	golden
miser	*misera*	*miserum*	wretched
piger	*pigra*	*pigrum*	fat, lazy
ruber	*rubra*	*rubrum*	red
pulcher	*pulchra*	*pulchrum*	beautiful
3rd declension			
puter	*putris*	*putre*	rotten
celer	*celeris*	*celere*	rapid
*facilis**	*facilis*	*facile*	easy
facilior	*facilior*	*facilius*	easier
maior	*maior*	*maius*	more
minor	*minor*	*minus*	less
simplex	*simplex*	*simplex*	simple
egens†	*egens*	*egens*	needy

*Most common types.
†Infinitive (present) participle used as adjective

(b) Participles are treated as if they are adjectives, i.e., they fall under Rule 12c (1) of the Code.

(c) Infinitive (also named 'present') participles in the singular do not change with gender. According to the four conjugations of Latin, they end in -*ans* (first conjugation, i.e., *vorans* devouring, from *vorare* to devour, *voro* I devour), -*ens* (second conjugation, i.e., *inhibens* inhibiting, from *inhibere* to inhibit, *inhibeo* I inhibit), -*ens* (third conjugation, i.e., *exigens* demanding, from *exigere* to demand, *exigo* I demand), -*iens* (third conjugation, i.e., *faciens* making, from *facere* to make, *facio*, I make), -*iens* (fourth conjugation, i.e., *oboediens* obeying, from *oboedire* to obey, *oboedio* I obey).

(d) Perfect participles change their endings with gender and are handled like adjectives of the first and second declension, e.g., *aggregatus* (masc.), *aggregata* (fem.), *aggregatum* (neut.) (aggregated, from aggregare to get together), *flexus*, *flexa*, *flexum* (bent, from *flectere* to bend), *latus*, *lata*, *latum* (carried, from the irregular verb *ferre* to carry), *diminutus*, *diminuta*, *diminutum* (smashed, from *diminuere* to smash).

(3) *Nominative nouns in apposition as specific epithets*

(a) In grammar, apposition means 'the placing of a word or expression beside another so that the second explains and has the same grammatical construction as the first';
i.e., the added nominative noun has an explanatory specifying function for the generic name. Thus, *Desulfovibrio gigas* may be understood as *Desulfovibrio dictus gigas* and translates as '*Desulfovibrio*, called the giant'.

(b) All specific epithets ending with the Latin suffixes -*cola* (derived from *incola*, 'the inhabitant, dweller') and -*cida* ('the killer') are examples of such nominative nouns in apposition.

4) *Genitive nouns as specific epithets*

(a) The singular genitive of nouns can be found in dictionaries.

(b) If the plural genitive is preferred, as for example in *Paenibacillus plantarum* ('of plants'), the declension of the noun must be determined, as plural genitives are different in different declensions [see F (3)].

Examples: *Curtobacterium plantarum* (first declension); *Staphylococcus equorum* (second declension); *Bifidobacterium dentium* (third declension); examples have not yet been found of the fourth and fifth declensions.

D. Formation of Prokaryote Names from Personal Names

(1) Persons may be honoured by using their name in forming a generic name or a specific epithet. However, the *Code* recommends refraining from naming taxa after persons that are not connected with microbiology or, at least, with natural science [see Recommendations 10a(1) and 12c(3)].

(2) *Latinization of personal names* (Table 2).

(a) It is good practice, where possible, to ask the person to be honoured by a scientific name for permission to use their name and to consider their preferences for how the name is Romanized and Latinized.

(b) Authors should refrain from naming bacteria after themselves or co-authors in the same publication [see Recommendations 6(7) and 12c(3)].

(c) The formation of prokaryote names from personal names has no geopolitical meaning and cannot be used to express geopolitical claims [see General Consideration 8].

(d) More than one person can be honoured in a single generic name or epithet. Examples: *Lechevalieria* after Hubert and Mary Lechevalier and *kimseyorum*, after Lynn S. and Robert B. Kimsey.

(e) When the personal name is already a Latin name, no further Latinization is needed, e.g., Julia, Victoria, Leo. When originating from languages written in non-Roman scripts, personal names should be rendered in the Roman alphabet according to an existing romanization scheme for the name-bearer's language or, where no such scheme exists, through transcription of the spoken form.

(f) Hyphens and white spaces must be removed from any components of a personal name used to build a scientific name [see Rule 12a]. Diacritics must be removed according to the conventions of the language of the name-bearer, e.g., German surnames Höffner and Müller become Hoeffner and Mueller, whereas the Turkish surname Özgür becomes Ozgur and the Finnish surname Törönen becomes Toronen [see Rule 64].

(g) A Romanized derivative of the personal name is used to create a Latinized stem to which suffixes can be added or which can be used in compound word formation. The choice of which components of the personal name are used to create the Latinized stem should consider the wishes of the person being honoured, relevant traditions (e.g., respecting cultures where multiple family names are used or none) and the goal of creating agreeable

user-friendly scientific names (see Recommendation 6). When someone has multiple family names, just one or two of these can be used to honour them. Example: *Boudabousia*, honouring Abdellatif Boudabous Chihi, *Streptomyces lunalinharesii* honouring Luiz Fernando de Toledo Luna Linhares, *Citrobacter murliniae* honouring Alma C. McWhorter-Murlin.

(h) Although a family name is most often used, generic names or specific epithets can also be formed from given names. Examples: *Erwinia* after Erwin Smith, *Jutongia* after Wú Jùtōng, *Staphylococcus arlettae* after Arlette van de Kerckhove and *Nocardia bhagyanarayanae* honouring Bhagyanarayana Gaddam. Scientific names can also be formed from given names placed before or after family names conjoined without a connecting vowel. Examples: *Elizabethkingia* honouring Elizabeth King, *Yonghaparkia* honouring Yong-ha Park and *Gaoshiqia* honouring Gāo Shíqí.

(i) Kinship prefixes (e.g., Ó/ Ní, Mac/Mc, ibn/bin/bint, Abu, ap and Ben) and particles and articles (e.g., al, de, le, van and von) may be omitted or included in the Latinized stem. Examples: *Rochalimaea* after da Rocha-Lima and *Leclercia* after Le Clerc. However, honorific titles (e.g., Sir, Sri and Sheikh) are not usually included in prokaryote names.

(j) To render the Latinized stems easy to pronounce, simplified or truncated forms of personal names may be adopted. Examples: *chauvoei* after Chauveau; *Simkania* after Simona Kahane and *jeikeium* after Johnson and Kaye. Additional vowels should be added if needed (e.g., *macginleyi* after Kenneth John McGinley). Intentional Latinizations involving changes in orthography of personal names must be preserved [see Rule 60].

(k) When the personal name ends in -*a*, the name should be treated as a first-declension Latin noun, where the stem is formed by removing the -*a*. When the personal name is a Latin name that ends in -*us*, the name should be treated as a second-declension Latin noun, where the stem is formed by removing the -*us*. When the personal name ends in -*o*, the name should be treated as a third-declension Latin noun, where the stem is formed by adding -*n* to give a genitive form ending in -*onis*.

(l) When a family name is used, there is the option to augment the stem by adding -*i* to the end of a family name, e.g., as in the epithet *youngii* in *Xanthomonas youngii*, after John Young. However, this is optional, as seen in *Citrobacter youngae*, which honours Viola Young. Stem augmentation is generally avoided when the Latinized stem ends in a vowel, e.g., the epithet *voltae* not *voltiae* from Volta; *sakazakii* not *sakazakiii* from Sakazaki and is inappropriate for components other than family names. Some authorities suggest stem augmentation should be avoided for names ending in -*er*, e.g., in the genus name *Buchnera* or species epithet *brenneri*. However, for consistency, the suffix -*ia* can be used when forming genus names in such cases e.g., *Listeria* or *Burkholderia*.

Table 2. Latinization of personal names

Romanization of the personal name
• If the personal name originates from a language not written in the Roman alphabet, romanize the personal name in accordance with conventions for the language of origin and the preferences of the personal being honoured. When no suitable romanization system exists, transcribe the name phonetically.

Creation of a Latinized stem

- Choose one or more components of the personal name suitable for creation of an agreeable and distinctive scientific name. Avoid components that are too short, too long or otherwise difficult to render in Latin (e.g., containing too few or too many vowels or consonants).
- Where a single component of the name cannot be on its own used to create an agreeable and distinctive Latinized stem, use additional or alternative components of the personal name (e.g., a given name or a given name combined with a family name) or explore additional options for Romanization.
- Remove diacritics, punctuation marks, hyphens and spaces to create a Latinized stem. Replacement of characters with diacritics should be undertaken in accordance with customs for the language of origin. If the personal name originates in the German language, characters with umlauts should be replaced by an appropriate digraph: ä is replaced by *ae*, ö by *oe* and ü by *ue*. However, in other languages (e.g., Turkish or Finnish), the vowel with an umlaut should be replaced by the same vowel without the umlaut. For personal names originating from Nordic languages, *ø*, *æ* and *å* become *oe*, *ae* and *aa*.
- For names of Gaelic origin, expand the patronymic prefix *Mc* to *Mac*.
- Optional: if the family name ends in a consonant, add -*i* to the original stem to create an augmented stem.
- Convert the stem to lower case before use in word formation.

Assignment to a declension

- If the personal name is already a Latin personal name or Latin word, then select the Latinized stem and declension already associated with the term (e.g., first declension if name ends in -*a*, second declension if the name ends in -*us*).
- The names *Andreas*, *Cosmas*, *Thomas* and *Tobias* are assigned to the first declension with a stem revealed by removing the final as. *Alexander* is assigned to the second declension with the stem *alexandr*-.
- The names *Michael*, *Raphael*, *Ruben* and *Simon* are assigned to the third declension with stems identical to the nominative form. The names *Felix*, *Leo*, *Paris* and *Vitalis* are assigned to the third declension with stems *felic*-, *leon*-, *parid*- and *vital*-. The name *Jesus* is treated as an irregular fourth-declension noun with the stem *jesu*- and the genitive form *jesu*.
- If the personal name ends in -*a*, assign the Latinized stem to the first declension.
- If the personal name ends in -*o*, add -*n* to end of the name to create a Latinized stem and assign the Latinized stem to the third declension.
- Otherwise, assign the stem to the first declension if the name bearer is female or to the second declension if the name bearer is male.

(3) *Personal names in generic names* (Table 3)

There are three suggested ways to form a generic name from the Latinized stem of a personal name:

(a) directly, by adding the feminine ending -*ia* (or -*a* if the Latinized stem ends in *e*, *i* or *u*, or -*aea* when the original personal name ends in -*a*).

(b) as a diminutive, usually by adding the feminine ending -*ella* to the stem. When the stem ends in -*e*, the final letter should be omitted when forming diminutives, e.g., in forming *Brucella* from the personal name Bruce. When digraph -*ee* or the vowel -*y* occurs at the end

of personal names, this may be Latinized as *i* when forming diminutives. Examples: *Cowdria* and *Roseburia* from Cowdry and Rosebury or the species epithet *asburiae* after the family name Fife-Asbury.

(c) by using a stem derived from a personal name as a word element in a compound name. Here the stem is linked, as needed, to the following word element by a connecting vowel. Example: *Youngimonas* or *Youngiimonas*, built from the stem *young*- (derived from the family name Young with stem augmentation), optionally the connecting vowel -*i*, and N.L. fem. n. *monas*.

Table 3. Formation of generic names from Latinized stems derived from personal names

To generate a simple genus name from a Latinized stem

- If the stem belongs to the first declension, add -*aea* to the stem.
  Example: *Rochalimaea* from the stem *rochalima*- built from the personal name da Rocha Lima.
- If the stem does not belong to the first declension and ends in a consonant, add -*ia* to the stem.
  Examples: *Escherichia* from the stem *escherich*- built from the personal name Escherich.
- If the stem does not belong to the first declension and ends in a vowel, add -*a* to the stem.
  Examples: *Beneckea* from the stem *benecke*-.
- Convert the first letter of the stem to upper case.
- Convert the genus name to italics.

To generate a diminutive genus name from a Latinized stem

- If the stem ends in -*e* add -*lla*. Example: *Brucella* built from the stem *bruce*-.
- Otherwise, add -*ella*. Example: *Salmonella* from the stem *salmon*-.
- Convert the first letter of the genus name to upper case
- Convert the genus name to italics.

To generate a compound genus name from a Latinized stem

- If the stem ends in -*i*, combine the stem and a subsequent word component without a connecting vowel. Example: if the theoretical name *Terasakimonas* were to be formed from the stem *terasaki*-
- Otherwise, combine the stem and a subsequent word component with the connecting vowel -*i*. Example: *Youngimonas*, built from the stem *young*- from the family name Young.
- Convert the first letter of the genus name to upper case.
- Convert the genus name to italics.

(4) *Personal names in specific epithets* (Table 4)

Two possibilities exist to form specific epithets from personal names

(i) creation of a genitive noun by addition of an inflectional ending to the Latinized stem so that an epithet is formed with the meaning of ‘pertaining to the person being honoured’. The inflectional ending is chosen to reflect the sex of the person to be honoured: typically the feminine ending -*ae* for a female or the masculine or neuter ending -*i* for a male or non-binary individual, except where a male name belongs to first declension, e.g., *voltae* from Volta or a name from a female belongs to the second declension, e.g., *pistorii* to honour a

female with the surname *Pistorius* or where a name of any gender has been assigned to the third declension, e.g., *mayonis* after Mark Mayo. Where the personal name resembles a Latin genitive, it may be used unchanged as a species epithet, e.g., *imshenetskii* to honour Aleksandr Imshenetskii, if it can be formed as described above.

(ii) creation of an adjectival form by adding the endings -*anus* (masculine), -*ana* (feminine) or -*anum* (neuter) to the stem according to gender of the genus name.

Note: The changes proposed in this version of section D take effect on 1 January 2025 and are not retroactive. Names validly published before that date that do not comply with this version of Section D may not be considered to be misspelled for that reason.

Table 4. Formation of specific epithets from Latinized stems derived from personal names

To generate a genitive species epithet<br>• If the stem belongs to the first declension, add -*ae* to the stem. Example: *voltae* from the stem *volt*- from the personal name Volta<br>• If the stem belongs to the second declension, add -*i* to the stem. Example: *adleri* from the stem *adler*- from the personal name Adler<br>• If the stem belongs to the third declension, add -*is* to the stem. Example: *mayonis* from the stem *mayon*- from the personal name Mayo<br>• Convert the species epithet to italics.
To generate an adjectival species epithet<br>• If the genus name is masculine, add -*anus* to the stem. Example: *migulanus* from the stem *migul*- from the personal name Migula.<br>• If the genus name is feminine, add -*ana* to the stem. Example: *olleyana* from the stem *olley*- from the personal name Olley.<br>• If the genus name is neuter, add -*anum* to the stem. Example: *lianum* from the stem *li*- from the personal name Li.<br>• Convert the species epithet to italics.

The changes proposed in this version of section D take effect on 1 January 2025 and are not retroactive. Names validly published before that date that do not comply with this version of section D may not be considered to be misspelled for that reason.

E. Formation of Prokaryote Names from Geographical Names

(1) The formation of prokaryote names from geographical names has no geopolitical meaning, i.e., such names cannot be used to express geopolitical claims (see General Consideration 8).

(2) Similar to personal names (Table 2 of Appendix 9), names of locations can be used to form generic names of the feminine gender. The preferred ending of such names is –*ia* or (diminutive form) –*ella*. Examples are shown in Table 5.

Table 5. Ways to form non-compound generic names from geographical names

**Geographical name ending in**	**Geographical name**	**Direct formation**	**Geographical name**	**Diminutive formation**

		**Direct ending**	**Example**		**Diminutive ending**	**Example**
consonant or –u	Delft, the Netherlands	add –ia	*Delftia*	Daejeon, Korea	add –ella	*Daejeonella*
	Baekdu, a mountain on the Chinese-North Korean border		*Baekduia*			
–us, –a, or –um	Praga, the Latin name of Prague, Czechia	drop ending, add –ia	*Pragia*	Silva Nigra, the Latin name of the Schwarzwald (Black Forest), Germany	drop ending, add –ella	*Silvanigrella*
–ius, –ia, or –ium (note that if name ends in –ia, the name is left unchanged)	Budvicium, the Latin name of České Budějovice, Czechia	drop ending, add -ia	*Budvicia*	Lusitania, the Latin name of Portugal	drop –us, –a, or –um, add –ella	*Lusitaniella*
	Hafnia, the old Latin name of Copenhagen, Denmark		*Hafnia*	-	-	
	Massilia, the Latin name of Marseille, France		*Massilia*			
–o	Dokdo, an island in the Korean Sea	add –nia	*Dokdonia*	Dokdo, an island in the Korean Sea	add –nella	*Dokdonella*
				Lutao, an island in the Pacific Ocean		*Lutaonella*
–e or –i	Jacksonville, a city in Florida (USA)	add –a	*Jacksonvillea*	Lone Pine Koala Sanctuary, Australia	drop –e or –i, add –ella	*Lonepinella*

Note: *Basilia* (Basilia, the Latin name of Basel, Switzerland) would be the preferred form instead of *Basilea*. *Scandinavia* would be the preferred form instead of *Scandinavium*. Instead of *Nevskia*, named after the Neva river, Russia, *Nevia* would be the preferred name.

Similar to personal names (see Table 3 of Appendix 9 in the 2022 revision of the ICNP [2]), names of geographical locations can be used to form compound generic names. The guidelines are presented in Table 6

Table 6. Ways to form compound generic names from a geographical name as non-terminal compound

**Geographical name ending in**	**Modify ending**	**Examples of geographical names**	**Examples of compound genus names**
any consonant	add connecting vowel –i, unless the following word	Eilat, a city and the Gulf of Eilat in Israel	*Eilatimonas*

	element begins with a vowel		
		Gabon, a country in Central Africa	*Gabonibacter*
		Senegal, a country in West Africa	*Senegalimassilia*
–i	add connecting vowel –i, unless the following word element begins with a vowel	Chiayi	*Chiayiivirga*
–a (and –us or –um in classical, medieval, or Neo-Latin names of locations)	drop ending; add connecting vowel –i, unless the following word element begins with a vowel	Bavaria, Germany	*Bavariicoccus*
		Frisinga, the Latin name of Freising, Germany	*Frisingicoccus*
		Massilia, the Latin name of Marseille, France	*Methanomassiliicoccus*
		Turicum, the Latin name of Zürich, Switzerland	*Turicibacter*, *Turicimonas*
–o	add –ni after the name of the location (–n if the following word element begins with a vowel)	SWIO, acronym of South West Indian Ocean	*Swionibacillus*

Note: *Turicicella* would be the preferred form instead of *Turicella*. '*Candidatus* Kapibacterium' (Motse Kapa in the Sesotho language) would be the preferred form instead of '*Candidatus* Kapaibacterium' or '*Candidatus* Kapabacteria'. '*Candidatus* Macondonimonas' (Macondo Prospect, Gulf of Mexico, and Macondo, a fictional town in *A Hundred Years of Solitude* by G. García Márquez) would be the preferred form instead of '*Candidatus* Macondimonas'. Note also that after a Greek geographical location, –o is used as connecting vowel.

Note: The recommendations and rules for specific epithets as given above are alsoapplicable for genus names, i.e., when the name of location such as above lends itself to translation into Latin, it can be further used for the formation of genus names following the guidelines in Table 5 or 6. Examples: *Flavimaricola* (from *Mare Flavum*, the Yellow Sea) and *Meridianimaribacter* (from *Mare Meridianum*, the South Sea).

(3) Unlike epithets derived from personal names, epithets created on the basis of geographical names should not be formed as nouns in the genitive case, but as adjectives. They usually are constructed by adding the ending –*ensis* (masculine or feminine gender) or -*ense* (neuter gender) to the geographical name and in agreement with the latter's gender. Only if the name of the locality ends in -*a* or -*e* or -*en*, are these letters dropped before adding -*ensis*/-*ense* (e.g., *jenensis* from Jena, *californiensis* from California, *drentensis* from Drente, *bremensis* from Bremen). If the locality's name ends in -*o*, the ending becomes -*nensis*/-*nense* (e.g., the name of the Japanese city Sapporo: *sapporonensis*, *sapporonense*).

(4) Quite a number of localities in the Old World (Europe, Asia, Africa) have classical Greek, Latin or medieval Latin names and adjectives derived from these: *aegyptius* (Egypt), *africanus* (Africa), *arabicus* (Arabia), *asiaticus* (Asia), *balticus* (Baltic Sea), *bavaricus* (Bavaria), *bretonicus* (Brittany), *britannicus* (Britain), *europaeus* (Europe), *frisius* (Friesland), *gallicus* (France), *germanicus* (Germany), *graecus* (Greece), *hellenicus* (Hellas, classical Greece), *helveticus* (Switzerland), *hibernicus* (Ireland), *hispanicus* (Spain), *hungaricus* (Hungary), *ibericus* (Spain/Portugal, the Iberian peninsula), *indicus* (India), *italicus* (Italy), *mediterraneus* (Mediterranean Sea), *persicus* (Persia, Iran), *polonus* (Poland), *rhenanus* (Rhineland), *romanus* (Rome), *saxonicus* (Saxony), etc. Neo-Latin names were given also to many other non-European parts of the world, so adjectives like *americanus* (America), *antarcticus* ('southern' in classical Latin) (Antarctica), *australicus* (Australia), *cubanus* (Cuba), *mexicanus* (Mexico), *japonicus* (Japan), etc. were introduced. Wherever such older adjectives exist, they may be used as specific epithets to indicate geographical origins.

(5) European and Mediterranean cities and places of classical times may have had quite different names than today, e.g., *Lucentum* (Alicante, Spain), *Argentoratum* (Strasbourg, France), *Lutetia* (Paris, France), *Traiectum* (Utrecht, Netherlands), *Ratisbona* (Regensburg, Germany), *Eboracum* (York, UK), *Londinium* (London, UK) and *Hafnia* (København, Denmark), which lead to the respective adjectives *lucentensis*, *argentoratensis*, *lutetiensis*, *traiectensis*, *ratisbonensis*, *eboracensis*, *londiniensis* and *hafniensis*. Numerous additional examples are listed in https://en.wikipedia. org/wiki/Category:Lists_of_Latin_place_names (accessed: 4 October 2024 [TO BE UPDATED FURTHER]). Alternatively, the Neo-Latin adjectives of the modern names may be used: *alicantensis*, *strasbourgensis*, *parisensis*, *utrechtensis*, *regensburgensis*, *yorkensis*, *londonensis*, *kobenhavnensis*, respectively.

(6) Many localities (mostly lakes, rivers, seas, islands, capes, rocks, mountains or valleys, but also some cities and towns) have names that consist of two words, usually an adjective and a noun (e.g., Deep Lake, Black Sea, Red River, Rio Grande, Long Island, Blue Mountain, Baton Rouge, Santa Cruz, Saint Germain, Sankt Georgen, etc.) or two nouns (e.g., Death Valley, Lake Windermere, Loch Ness, Martha's Vineyard, Ayers Rock, Woods Hole, Cape Cod, Monte Carlo, etc.). The formation of specific epithets from the names of such localities may pose a problem, as the use of the adjectival suffix -*ensis*, -*ense* may lead to rather strange looking or awkward constructions, such as '*deeplakensis*' or '*bluemountainense*', although they would be formally correct. If the name of a locality lends itself to translation into Latin, specific epithets may be formed, as well as genitive nouns of the two components and concatenating them without hyphenation, such as the existing *lacusprofundi* (of Deep Lake), *marisnigri* (of the Black Sea), *marismortui* (of the Dead Sea) or, of two nouns, *vallismortis* (of Death Valley). See also Section A (1) (b) above.

(7) The inclusion of articles (such as, *the*, *el*, *o*, *il*, *le*, *la*, *a*, *de*, *der*, *die*, *das*, *den*, *het* or their plurals *the*, *los*, *las*, *os*, *as*, *les*, *ils*, *gli*, *le*, *de*, *die*, '*s*, etc.) as they are used for locations in several languages (e.g., La Paz, El Ferrol, El Alamein, Le Havre, The Netherlands, Die Schweiz, Den Haag, 's Hertogenbosch, Los Angeles, etc.) should be avoided.

F. Formation of Names for Prokaryotes Living in Association or Symbiosis with Other Biota

(1) For the formation of names for prokaryotes that live in association or symbiosis with plants, fungi, animals or other prokaryotes, it is important to know the exact meaning of the nomenclatural name of such a partner and how it was formed (adjective, genitive noun, etc.).

(2) The most common way of forming such specific epithets is the use of the genitive case of the generic name of the associated organism in question, e.g., *suis*, *equi*, *bovis*, *muscae*, *muris*, *aquilae*, *falconis*, *gypis*, *elephantis* (of the pig, horse, cow, fly, mouse, eagle, falcon, vulture, elephant), or *fagi*, *quercus* (4th declension genitive, spoken with long u), *castaneae*, *aesculi*, *rosae*, *liliae* (of the beech, oak, chestnut, horse chestnut, rose, lily).

(3) Alternatively, the genitive of the plural is recommended, especially if several species of the associated (usually) eukaryotic genus house the prokaryote species in question. To form the plural genitive, one needs to know the stem and declension of the word. The following examples may be of assistance:

(a) 1st declension: -*arum* (*muscarum*, of flies, *rosarum*, of roses);

(b) 2nd declension: -*orum* (*equorum*, of horses, *pinorum*, of pines);

(c) 3rd declension (consonant stems): -*um* (*leonum*, of lions, *leguminum*, of legumes);

(d) 3rd declension (vocal and mixed stems): -*ium* (*felium*, of cats, *ruminantium*, of ruminants);

(e) 4th declension: -*um* (*quercum*, of oaks);

(f) 5th declension: -*rum* (*scabierum*, of different forms of scabies, a skin desease).

Note. Be aware of irregular forms such as *bos* (the cow), genitive *bovis*, plural genitive *boum*; *canis* (the dog), genitive *canis*, plural genitive *canum*. Use dictionaries.

G. Names Originating from Languages Other than Latin or Classical Greek

(1) According to Recommendation 6(3), Words from languages other than Latin or Classical Greek should be avoided as long as equivalents exist in Latin or Greek or can be constructed by combining word elements from these two languages.

Example: The formation of the epithet *simbae* from the East African Swahili word *simba*, lion, for a *Mycoplasma* species contravenes Recommendation 6(3).

Only Latin case endings are permitted. Greek endings should be transformed into Latin endings.

(2) When a word from another language is used, the word stem must be identified before Latinization.

Example: The Arabic word ‘alkali’ (*al-qaliy*, the ashes of saltwort) from which the element kalium (K; English, potassium) received its name. Since the -*i* at the end of the word belongs to the stem, it is wrong to speak and write, of alcalophilic, instead of alkaliphilic microbes. Formally *alkaliiphilus* (-*a*, -*um*) is then more correct than *alkaliphilus* (-*a*, -*um*), etc., but in view of the many precedents in the past, addition of a connecting vowel after *alkali*- is not recommended.

(3) Typical usages of other languages should not be carried over into Latin.

Example: The English suffix -philic (e.g., hydrophilic: friendly to water, water-loving) is an English transformation of the Latin -*philus*, -*a*, -*um* (originating from Greek *philos*, friendly). Therefore, the ending -*philicus* should be avoided and -*philus* should be used instead.

(4) National food or fermentation products (e.g., sake, tofu, miso, yogurt, kvas, kefir, pombe, pulque, aiva, etc.) often do not have equivalent Latin names, although microorganisms may be named after such food or food products if found in them or causing fermentations. These names must not be used unaltered just as specific epithets in the form of nominative nouns in apposition. They are properly Latinized by forming a neuter noun by adding -*um* (e.g., *sakeum*, *tofuum*, *kefirum*, *pombeum*, etc.) and the use of the genitive of that (ending -*i*) in the specific epithet (e.g., *sakei*, *tofui*, *kefiri*, *pombei*, etc.).

H. Formation of Prokaryote Names from Names of Elements and Compounds Used in Chemistry and Pharmacy

(1) The vast majority of names of chemicals are Latinized as neuter nouns of the 2nd declension with nominatives ending -*um*, genitives in -*i*. The following groups belong in this category:

(a) Most of the chemical elements with the exception of carbon (L. *carbo*, *carbonis*) phosphorus (L. *phosphorus*, *phosphori*) and sulfur (L. *sulfur*, *sulfuris*) have the ending -(*i*)*um* with the genitive ending in -(*i*)*i*; nitrogen may also be called *azotum* besides *nitrogenium*, *calcium* may also be called calx (genitive *calcis*).

(b) Names of chemical and biochemical compounds ending in -ide (including anions), -in, -*ane*, -*ene*, -*one*, -*ol* (only non-alcoholic compounds), -*ose* (sugars), -*an* (polysaccharides) and -*ase* (enzymes) are Latinized by adding the ending -*um* or by replacing the -*e* at the end by -*um* as appropriate.

(c) Acids are named by *acidum* (L. neuter noun, acid), followed by a descriptive neuter adjective, e.g., sulfurous acid *acidum sulfurosum*, sulfuric acid *acidum sulfuricum*, acetic acid *acidum aceticum*.

(2) The second largest category of chemicals are treated as neuter nouns of the 3rd declension: These end in -*ol* (the alcohols), -*al* (aldehydes), -*er* (ethers, esters) and -*yl* (organic radicals); Latinization does not change their names at the end, whereas the genitive is formed by adding -*is*.

(3) Anions ending in -*ite* and -*ate* are treated as masculine nouns of the 3rd declension. The English ending -*ite* is Latinized to -*is*, with the genitive -*itis*, e.g., nitrite becomes *nitris*, *nitritis*. The English ending -*ate* is Latinized to -*as*, with the genitive -*atis*, e.g., nitrate becomes *nitras*, *nitratis*.

(4) Only a few chemicals have names that are Latinized in the 1st declension as feminine nouns, ending in -*a* with a genitive in -*ae*. Besides chemicals that always had names ending in -*a* (like urea), these are chemicals found in classical and medieval Latin, such as gentian (*gentiana*) and camphor (*camphora*), as well as modern drugs, wherein the Latin names were formed by adding -a, such as the French ergot, becoming *ergota* in Latin. An important group of this category are alkaloids and other organic bases, such as nucleic acid bases and

amino acids with English names ending in -*ine*. In Neo-Latin this ending is -*ina*, with the genitive -*inae*.

Examples: *betaina*, -*ae*; *atropina*, -*ae*; *adenina*, -*ae*; *alanina*, -*ae*.

(5) The word stems and genitives of Latinized chemical names are the basis for their use in prokaryote generic names and specific epithets. In principle, they are then treated like any other word elements.

I. Arbitrary Names

(1) The basis for arbitrary names are Rules 10a and 12c of the Code: 'genus names or specific epithets may be taken from any source and may even be composed in an arbitrary manner'. They must, however, be treated as Latin. Often they are vocalized abbreviations or contractions of names. Examples: *Cedecea*, *Afipia*, *Kordia*, *Kribbella*, *Waddlia* and *Desemzia*, that were derived from the acronyms CDC (Centers for Disease Control), AFIP (Armed Forces Institute of Pathology), KORDI (Korea Ocean Research and Development Institute), KRIBB (Korean Research Institute of Bioscience and Biotechnology), WADDL (Washington Animal Disease Diagnostic Laboratory) and DSMZ (Deutsche Sammlung von Mikroorganismen und Zellkulturen), respectively. Another example is *Simkania* (contracted from the name Simona Kahane). Examples for arbitrary specific epithets are (*Burkholderia*) *unamae*, derived from the acronym UNAM (Universidad Nacional Autónoma de México), (*Brevundimonas*) *nasdae*, derived from the acronym NASDA (National Space Development Agency of Japan), and (*Flavobacterium*) *micromati* derived from the abbreviation MICROMAT (MICROMAT project 'Biodiversity of Microbial Mats in Antarctica').

Arbitrary specific epiths based on acronyms, e.g., of names of research institutions, universities, etc. are preferentially formed as nouns in the genitive case. Use of adjectives with -(*i*)*anus*, -(*i*)*ana*, (*i*)*anum* endings is possible, as well.

(2) When proposing arbitrary names or epithets, authors should aim at short, elegant, easily spelled and pronounced ones.

Note. With arbitrary genus names, the gender should also be indicated.

References 99-113 are intended to be informative and helpful, but are not an official part of Appendix 9.

APPENDIX 10. INFRASUBSPECIFIC SUBDIVISIONS

The designations of these taxa are not covered by the Rules of this Code, but this Appendix is included to encourage conformity and to clarify the application of these designations (see Rule 14a, b).

A. Definitions

The term **infrasubspecific subdivision** (or division) has been used in two ways to denote both terms and taxa. It is preferable to distinguish them as given below. **Infrasubspecific "subdivision"** has been used rather than "division" to avoid any confusion with the taxonomic category "division" (*divisio*) used in the botanical and the zoological nomenclature.

Note. Infrasubspecific subdivisions are not arranged in any order of rank, and may overlap one another.

(1) *Infrasubspecific taxa*. An **infrasubspecific taxon** is one strain or a set of strains showing the same or similar properties, and treated as a taxonomic group.

Example: *Staphylococcus aureus* phagovar 81.

The sets of properties used may be of a similar kind but are not necessarily the same.

Example: The susceptibility to a different phage may be used to define another phagovar of *Staphylococcus aureus*, e.g., phagovar 42D.

Infrasubspecific taxa based on different sets of properties may overlap; e.g., one serovar may contain strains belonging to different phagovars.

Example: *Salmonella enterica* serovars, phagovars, and biovars.

(2) *Infrasubspecific terms*. An **infrasubspecific term** is used to refer to the kinds of taxa below subspecies.

Examples: serovar, chemovar, *forma specialis*.

If a species has not been divided into subspecies, the infrasubspecific terms may be applied to other subdivisions within that species. The subdivisions so named would still be infrasubspecific subdivisions for nomenclatural purposes until they may be raised to subspecific or specific rank.

Example: Serovars of *Erysipelothrix rhusiopathiae*.

(3) *Use of other terms*. **Infrasubspecific form** has been used to refer to a bacterial strain, although this use should be avoided.

A **culture** of prokaryotes is a population of bacterial cells in a given place at a given time, e.g., in this test tube or on that agar plate. It may have a long duration, e.g., desiccated cultures.

A **clone** is a population of prokaryotic cells derived from a single parent cell.

A **strain** is made up of the descendants of a single isolation in pure culture. A strain is usually made up of a succession of cultures and is often derived from a single colony. The number of cells that gave rise to the original colony is often unknown.

Individual is a term with little meaning in bacteriology although it has been applied to a single prokaryotic cell or to a bacterial strain; it is best to avoid the use of this term.

B. Infrasubspecific Terms

The table below contains some of the terms that are commonly used, and the preferred name appears in the first column. The introduction of the suffix **"-var"** or **"-form"** to replace "-type" is recommended to avoid confusion with the strict use of the term "type" to mean nomenclatural type (see Rule 15).

Infrasubspecific terms

**Preferred name**	**Synonym(s)**	**Notes**
Biovar	Biotype, physiological type	Biochemical or physiological properties
Chemoform	Chemotype	Chemical constitution
Chemovar		Production or amount of production of a particular chemical
Cultivar		A cultivated strain with particular properties
forma specialis (abbreviation, f.sp.)	Special form	A parasitic, symbiotic, or commensal microorganism distinguished primarily by adaptation to a particular host or habitat. Named preferably by the scientific name of the host, in the genitive case
Genomovar	Genovar, genomic group	Used to designate distinct intraspecific groups based on genomic comparisons, that cannot be phenotypically distinguished
Morphovar	Morphotype	Morphological characteristics
Pathovar	Pathotype	Disease responses in one or more hosts. For recommendations on designating pathovars and use of designations when reviving names, see [49] in Appendix 3
Phagovar	Phagotype, lysotype	Reactions to bacteriophage
Phase		Restricted to well-defined stages of naturally occurring alternating variations
Serovar	Serotype	Antigenic characteristics
State		Colonial variants, e.g., rough, smooth, mucoid (may be defined antigenically)

The term **"type"** in prokaryotic biology (e.g., phenotype, genotype, serotype, etc.) must not be confused with the strictly nomenclatural use of the term, type (Principle 5 and Chapter 3, Section 4).

The term **"group"** is informal and has no nomenclatural standing. It may prove useful to designate informally a set of organisms having certain characteristics in common, provided that it is used with care and exact definition to avoid ambiguity. It must not be used to avoid the use of the correct name of a taxon such as genus or species. However, it may be useful

when the bacteriologist does not wish to give a formal name to a set of prokaryotes until further studies have been made but wishes to publish his results and seek the opinion of others.

Example: "IID group," later named *Cardiobacterium hominis*.

C. Nomenclature of Infrasubspecific Taxa

An **infrasubspecific taxon** is designated or cited by the name of the species followed by the infrasubspecific term used to designate this infrasubspecific subdivision followed by the infrasubspecific designation.

Example: *Staphylococcus aureus* phagovar 81.

Reference strains of infrasubspecific taxa may be designated.

There are many ways that infrasubspecific taxa may be designated; among these are the following: Latinized words, e.g., *cerealis* in *Xanthomonas translucens* f.sp. *cerealis*; vernacular names or words, e.g., rough phase; numbers, letters, or formulae, e.g., phagovar 42D in *Staphyloccocus aureus* phagovar 42D.

.

D. Nomenclature of Strains

A strain may be designated in any manner, e.g., by the name of an individual, by a locality, or by a number. Strain designations (e.g., strain collection accession numbers) must be preserved to ensure the 'chain of custody' of prokaryotes that are presumed to be the same but may demonstrate different features.

APPENDIX 11. THE PROVISIONAL STATUS *CANDIDATUS*

Introduction of the status called *Candidatus* was first proposed by Murray and Schleifer in 1994 [109]. The provisional status *Candidatus* was intended for putative taxa of any rank that could not be described in sufficient details to warrant establishment of a novel taxon, usually because of the absence of a pure culture. Following discussions of the International Committee on Systematics of Bacteria (ICSB; now the International Committee on Systematics of Prokaryotes, ICSP) [110], further guidelines were published for *Candidatus* taxa in 1995 [111].
This status should be used for describing prokaryotic taxa for which more than a nucleic acid sequence is available but for which the requirements for valid publication of a name according to the *Code* are not met.

The following information should be included in the description of a *Candidatus* taxon:

(a) Genomic information, i.e., nucleic acid sequences apt to determine the phylogenetic position of the organism.

(b) All information so far available on structure and morphology (appropriate illustration), physiology and metabolism, reproductive features, the natural environment, in which the organism can be identified by *in situ* hybridization or similar techniques for cell detection and identification, and any other available and suitable information.

A name of an organism in the status of *Candidatus* consists of the word *Candidatus*, followed by a name, based on one of the ranks defined in this *Code* (species, genus, family, etc.), formed in accordance with the nomenclature rules of the *Code* and its etymology appendix (Appendix 9); see also [112].

Examples: *Candidatus* Methanoflorentaceae (family rank), *Candidatus* Methanoflorens (genus rank), *Candidatus* Methanoflorens stordalenmirensis (species rank).
Note that the word *Candidatus*, but not the name that follows, is printed in italics.

Before the implementation of Section 10, Rules 66-73 of the *Code*, *Candidatus* names had no pro-standing in prokaryote nomenclature; they still have no standing. A proposal to include names of *Candidatus* taxa under Rules of the ICNP as validly published names to *Candidatus* names [113, 114] was rejected by the ICSP in 2020 [115]. Starting 1 January 2025, the new rules to regulate nomenclature of *Candidatus* names are implemented.

Murray and Stackebrandt [111] proposed compiling a list of *Candidatus* taxa based on requests for inclusion submitted by the authors describing them. Starting 2020, lists of proposed *Candidatus* taxa have been published periodically in the IJSEM as a service to the scientific community [116–121]. Rather than proposed listing of a 'codified record' of each *Candidatus* taxon (as suggested in [111]), these lists, compiled by the IJSEM List Editors, include the etymologies and references to the publications in which the names were proposed. If necessary, names were corrected, based on the rules of the *Code* and its Appendix 9 [116, 118-120]. Those corrections are proposals only, and alternative corrected names are possible. The *Candidatus* lists published in the IJSEM are not to be considered as 'Approved Lists of Names' that may serve as Validation Lists if, in the future, the ICSP may decide to include *Candidatus* taxa under the Rules of the *Code*. At the time of publication of the first two *Candidatus* lists in the IJSEM, the rank of phylum was not included in the Code, and, therefore, names of *Candidatus* phyla were not listed. As the ICSP has since included

the rank of phylum in the *Code*, the List Editors of the IJSEM have also prepared an initial list of *Candidatus* phyla [121]. Authors and other individuals wishing to have new names of *Candidatus* taxa included in future lists should send an electronic copy of the published paper to the IJSEM List Editors.

When an organism of the status *Candidatus* is later isolated and the pure culture sufficiently described, the name can be submitted for validation according to the Rules of the *Code*. The former *Candidatus* name is then deleted from the *Candidatus* list.

APPENDIX 12. THE VAN NIEL INTERNATIONAL PRIZE

The van Niel International Prize, established in 1986 by Professor V. B. D. Skerman of The University of Queensland, honours the contribution of scholarship in the field of microbiology by Professor Cornelis Bernardus van Niel.

A history of the prize and a list of recipients from 1986 until 2014 is presented in Appendix 12 of the 2008 Revision of the *ICNP* [1]. The prize was not awarded for 2014-2017.

2017–2020 *van Niel Prize recipient*, Tanja Woyke

The Senate of The University of Queensland, on the recommendation of the Executive Board of the International Committee on Systematics of Prokaryotes, is pleased to present the van Niel International Prize for Studies in Bacterial Systematics for the triennium 2017–2020 to Dr Tanja Woyke in recognition of her contributions made to the field of bacterial systematics [122].

2021–2024 *van Niel Prize recipient*, [TO BE UPDATED WHEN RELEVANT]

The Senate of The University of Queensland, on the recommendation of the Executive Board of the International Committee on Systematics of Prokaryotes, is pleased to present the van Niel International Prize for Studies in Bacterial Systematics for the triennium 2021–2024 to xxxxx

APPENDIX 13. ACTIVITIES OF THE CONGRESSES

The minutes of the meetings of the International Congress for Microbiology (and later, the International Congress of Bacteriology and Applied Microbiology) of the International Union of Microbiological Societies contain a detailed history of the evolution of this code of nomenclature. A summary of this historical material is presented in Appendix 13 of the 2008 Revision of the ICNP [3]. Minutes of the ICSP plenary meetings held between 2014 and 2019 are summarized in Appendix 13 of the 2022 Revision of the ICNP [1].

A plenary meeting of ICSP was held on 22 October 2024 in conjunction with the 18th Congress of the International Union of Microbiological Societies (IUMS) held in Florence, Italy. XXX XXX XXX XXX XXX INFORMATION TO BE COMPLETED LATER – MINUTES WERE SUBMITTED FOR PUBLICATION IN THE IJSEM

Funding information
The authors received no specific grant from any funding agency.

Conflicts of interest
The authors declare that there are no conflicts of interest.

References [NUMBERS ARE PROVISIONAL]

1. **Oren A, Arahal DR, Göker M, Moore ERB, Rossello-Mora R, *et al.*** International Code of Nomenclature of Prokaryotes. Prokaryotic Code (2022 Revision). *Int J Syst Evol Microbiol* 2023;73:005585.
2. **Whitman WB, Bull C, Busse H-J, Fournier P-E, Oren A, *et al.*** Request for revision of the Statutes of the International Committee on Systematics of Prokaryotes. *Int J Syst Evol Microbiol* 2019;69:584–593.
3. **Parker CT, Tindall BJ, Garrity GM (eds).** International Code of Nomenclature of Prokaryotes Prokaryotic Code (2008 Revision) *Int J Syst Evol Microbiol* 2019;69:S1–S111.
4. **Ash C**, **Priest FG**, **Collins MD**. *Paenibacillus* gen. nov. In *Validation of the Publication of New Names and New Combinations Previously Effectively Published Outside the IJSB*, List no. 51. *Int J Syst Bacteriol* 1994;44:852.
5. **Turland NJ, Wiersema JH, Barrie FR, Greuter W, Hawksworth DL, *et al.* (eds).** *International Code of Nomenclature for algae, fungi, and plants (Shenzhen Code) adopted by the Nineteenth International Botanical Congress Shenzhen, China, July 2017. Regnum Vegetabile 159.* Glashütten: Koeltz Botanical Books; 2018.
6. **Roop RM**, **Smibert RM**, **Johnson JL**, **Krieg NR**. Designation of the neotype strain for *Campylobacter sputorum* (Prévot) Véron and Chatelain 1973. *Int J Syst Bacteriol* 1986;36:348.
7. **Kelly DP**, **Wood AP**.Reclassification of some species of *Thiobacillus* to the newly designated genera *Acidithiobacillus* gen. nov., *Halothiobacillus* gen. nov. and *Thermithiobacillus* gen. nov. *Int J Syst Evol Microbiol* 2000;50:511–516.
8. **Sly LI**, **Cahill MM**, **Osawa R**, **Fujisawa T**. The tannin-degrading species *Streptococcus gallolyticus* and *Streptococcus caprinus* are subjective synonyms. *Int J Syst Bacteriol* 1997;47:893–894.
9. **Raj HD**. A new species - *Microcyclus flavus*. *Int J Syst Bacteriol* 1970;20:61–81.
10. **Cohn F**. Untersuchungen über Bakterien. *Beiträge Biol Pflanz* 1872;1:127–224.
11. **Cato EP**, **Moore WEC**, **Nygaard G**, **Holdeman LV**. *Actinomyces meyeri* sp. nov., specific epithet rev. *Int J Syst Bacteriol* 1984;34:487–489.
12. **Xie CH**, **Yokota A.** Reclassification of *Alcaligenes latus* strains IAM 12599T and IAM 12664 and *Pseudomonas saccharophila* as *Azohydromonas lata* gen. nov., comb. nov., *Azohydromonas australica* sp. nov. and *Pelomonas saccharophila* gen. nov., comb. nov., respectively. *Int J Syst Evol Microbiol* 2005;55:2419–2425.
13. **Goodfellow M**, **Pirouz T**. Numerical classification of sporoactinomycetes containing *meso*-diaminopimelic acid in the cell wall. *J Gen Microbiol* 1982;128:503–527.
14. **Bernardet J-F**, **Segers P**, **Vancanneyt M**, **Berthe F**, **Kersters K**, ***et al***. Cutting a gordian knot: emended classification and description of the genus *Flavobacterium*, emended description of the family *Flavobacteriaceae*, and proposal of *Flavobacterium hydatis* nom. nov. (basonym, *Cytophaga aquatilis* Strohl and Tait 1978). *Int J Syst Bacteriol* 1996;46:128–148.

15. **Pridham TG**, **Lyons AJ**, **Seckinger HL**. Comparison of some dried holotype and neotype specimens of streptomycetes with their living counterparts. *Int Bull Bacteriol Nomencl Taxon* 1965;15:191–237.
16. **Rogosa M**. *Peptococcaceae*, a new family to include the Gram-positive, anaerobic cocci of the genera *Peptococcus*, *Peptostreptococcus*, and *Ruminococcus*. *Int J Syst Bacteriol* 1971;21:234–237.
17. **Breed RS**, **Murray EGD**, **Smith NR**. (eds) *Bergey's Manual of Determinative Bacteriology*, 7th ed. Baltimore, MD: Williams & Wilkins; 1957.
18. **Schwabacher H**, **Lucas DR**, **Rimington C**. *Bacterium melaninogenicum*; a misnomer. *J Gen Microbiol* 1947;1:109–120.
19. **Lessel EF**. Status of the name *Proteus morganii* and designation of the neotype strain. *Int J Syst Bacteriol* 1971;21:55–57.
20. **Buchanan RE**, **Holt JG**, **Lessel EF**. *Index Bergeyana. An annotated alphabetic Listing of Names of the Taxa of the Bacteria*. Baltimore, MD: Williams and Wilkins; 1966.
21. **Sneath PHA**. Correction of orthography of epithets in *Pasteurella* and some problems with recommendations on latinization. Letter to the Editor. *Int J Syst Bacteriol* 1992;42:658–659.
22. **Buchanan RE, St. John-Brooks R, Breed RS.** International Bacteriological Code of Nomenclature in 4th International Congress for Microbiology, Report of Proceedings, July 20–26, 1947, Copenhagen; 1947. pp. 587–606.
23. **Buchanan RE, St. John-Brooks R, Breed RS.** International Bacteriological Code of Nomenclature. *J Bacteriol* 1948;55:287–306.
24. **Buchanan RE, St. John-Brooks R, Breed RS.** International Bacteriological Code of Nomenclature. *J Gen Microbiol* 1949;3:444–462.
25. *International Code of Nomenclature of Bacteria and Viruses.* Ames, IA: State College Press; 1958.
26. **Editorial Board.** International Code of Nomenclature of Bacteria (1966 Revision). *Int J Syst Bacteriol* 1966;16:459–490.
27. **Lapage SP, Sneath PHA, Lessel EF, Skerman VBD, Seeliger HPR, *et al.* (eds).** *International Code of Nomenclature of Bacteria and Statutes of the International Committee on Systematic Bacteriology and Statutes of the Bacteriology Section of the International Association of Microbiological Societies (1976 Revision).* Washington, DC: American Society for Microbiology; 1975. (Russian translation published 1978 by USSR Academy of Sciences, Moscow; Japanese translation published 1982 by Center for Academic Publications, Tokyo; Chinese translation published 1989 by Science Press, Beijing.)
28. **Lapage SP, Sneath PHA, Lessel EF, Skerman VBD, Seeliger HPR, *et al.* (eds).** *International Code of Nomenclature of Bacteria and Statutes of the International Committee on Systematic Bacteriology and Statutes of the Bacteriology and Applied Microbiology Section of the International Union of Microbiological Societies (1990 Revision).* Washington, DC: American Society for Microbiology; 1992. https://www.ncbi.nlm.nih.gov/books/NBK8817/ (accessed: 4 October 2024 [TO BE UPDATED FURTHER])
29. **Hedlund BP, Chuvochina M, Hugenholtz P, Konstantinidis KT, Murray AE, *et al.*** SeqCode: a nomenclatural code for prokaryotes described from sequence data. *Nat Microbiol* 2022;7:1702–1708.
30. **Brickell CD, Alexander C, Cubey JJ, David JC, Hoffman MHA, *et al.*** *International Code of Nomenclature for Cultivated Plants.* 9th ed. Scripta Horticulturae 18. Leuven: ISHS; 2016. https://www.ishs.org/scripta-horticulturae/international-code-nomenclature-cultivated-plants-ninth-edition (accessed: 4 October 2024 [TO BE UPDATED FURTHER])

31. *International Code of Zoological Nomenclature*, 4th ed. London, UK: The International Trust for Zoological Nomenclature; 1999. https://www.iczn.org/the-code/the-code-online/ (accessed: 4 October 2024 [TO BE UPDATED FURTHER])

32. *The International Code of Virus Classification and Nomenclature (ICVCN). March 2021.* https://ictv.global/about/code (accessed: 4 October 2024 [TO BE UPDATED FURTHER])

33. Greuter W, Garrity GM, Hawksworth DL, Jahn R, Kirk PM, *et al.* Draft BioCode (2011): Principles and rules regulating the naming of organisms. *Taxon* 2011;60:201–212. https://onlinelibrary.wiley.com/doi/10.1002/tax.601019 (accessed: 4 October 2024 [TO BE UPDATED FURTHER])

34. Theurillat J-P, Willner W, Fernández-González F, Bültmann H, Čarni A, *et al.* International Code of Phytosociological Nomenclature. 4th edition. *Appl Veget Sci* Appl Veg Sci. 2021;24:e12491.

35. de Queiroz K, Cantino P. *International Code of Phylogenetic Nomenclature (PhyloCode)*. Version 6. CRC Press, Taylor & Francis; 2020.

36. Skerman VBD, **Sneath PHA**, **McGowan V**. Approved Lists of Bacterial Names. *Int J Syst Bacteriol* 1980;30:225–420.

37. Hill LR, Skerman VBD, Sneath PHA. Corrigenda to the Approved Lists of Bacterial Names edited for the International Committee on Systematic Bacteriology. *Int J Syst Bacteriol* 1984;34:508-511.

38. Skerman VBD, **McGowan V**, **Sneath PHA**. *Approved Lists of Bacterial Names. Amended edition*. American Society for Microbiology, Washington, 1989.

39. Sneath PHA. The preparation of the approved lists of bacterial names. *Int J Syst Evol Microbiol* 2005;55:2247–2249.

40. Gibbons NE, Pattee KB, Holt JG. *Supplement to Index Bergeyana*. Baltimore: The Williams & Wilkins Co; 1982.

41. Moore WEC, Cato EP, Moore LVH (eds). Index of the Bacterial and Yeast Nomenclatural Changes Published in the *International Journal of Systematic Bacteriology* since the 1980 Approved Lists of Bacterial Names (1 January 1980 to 1 January 1985). *Int J Syst Bacteriol* 1985;35:382–407.

42. Moore WEC, Moore LVH (eds). *Index of the Bacterial and Yeast Nomenclatural Changes Published in the International Journal of Systematic Bacteriology since the 1980 Approved Lists of Bacterial Names (1 January 1980 to 1 January 1989).* Washington, DC: American Society for Microbiology; 1989.

43. *Bergey's Manual of Systematics of Archaea and Bacteria.* Published by John Wiley & Sons, Inc., in association with Bergey's Manual Trust. https://onlinelibrary.wiley.com/doi/book/10.1002/9781118960608 (accessed: 4 October 2024 [TO BE UPDATED FURTHER])

44. Dye DW, Bradbury JF, Goto M, Hayward AC, Lelliott RA, *et al.* Standards for naming pathovars of phytopathogenic bacteria and a list of pathovar names and pathotype strains. *Rev Plant Pathol* 1980;59:153–168.

45. Hauer T, Komárek J. CyanoDB 2.0 - On-line database of cyanobacterial genera. - *World-wide electronic publication, Univ. of South Bohemia & Inst. of Botany AS CR*; 2020. http://www.cyanodb.cz (accessed: 4 October 2024 [TO BE UPDATED FURTHER])

46. Silva PC. *Names of Classes and Families of Living Algae with Special Reference to Their Use in the Index Nominum Genericorum (Plantarum) (Regnum Vegetabile vol. 103)*. Utrecht: Bohn, Scheltema & Holkema; 1980.

47. Farr ER, Leussink JA, Stafleu FA (eds). *Index Nominum Genericorum (Plantarum), vols. 3 (Regnum Vegetabile vol. 100–102)*. Utrecht: Bohn, Scheltema & Holkema; 1979.

48. **Farr ER, Leussink JA, Zijlstra G (eds).** *Index Nominum Genericorum (Plantarum) Supplementum I (Regnum Vegetabile vol. 113).* Utrecht: Bohn, Scheltema & Holkema; 1986.
49. **Fourtanier E, Kociolek JP (comps).** Catalogue of Diatom Names, California Academy of Sciences (updated 19 Sep 2011). Available online at: https://researcharchive.calacademy.org/research/diatoms/names/index.asp (accessed: 4 October 2024 [TO BE UPDATED FURTHER])
50. **Guiry MD, Guiry GM.** AlgaeBase. World-wide electronic publication, National University of Ireland, Galway. Available online at: www.algaebase.org (accessed: 4 October 2024 [TO BE UPDATED FURTHER])
51. **Kusber W-H, Jahn R.** Annotated list of diatom names by Horst Lange-Bertalot and co-workers. Version 3.0; 2003. Available online at: www.algaterra.org/Names_Version3_0.pdf (accessed: 4 October 2024 [TO BE UPDATED FURTHER])
52. **Neave SA (ed).** *Nomenclator Zoologicus, 1758–1935.* 4 vols, 3 suppls. London: Zoological Society; 1939.
53. **Sharpe D (ed).** *Index Zoologicus*, 1800–1900. London: Zoological Society; 1902. Index Zoologicus. 1902–present. London: Zoological Society.
54. **Levine ND, Corliss JO, Cox FE, Deroux G, Grain J, *et al*.** A newly revised classification of the protozoa. *J Protozool* 1980;27:37–58.
55. **Aescht E.** *Catalogue of the Generic Names of Ciliates (Protozoa, Ciliophora).* Linz, Austria: Oberoesterreichisches Landesmuseum; 2001.
56. **Hawksworth DL, Sutton BC, Ainsworth GC.** *Ainsworth & Bisby's Dictionary of the Fungi*, 7th ed. Kew: Commonwealth Mycological Institute; 1983.
57. Index to Fungi. 1940–present (semi-annual). Wallingford: CAB International.
58. **Kirk P, Cannon P, Stalpers J, Minter D (ed).** *Dictionary of the Fungi*, 10th ed. CABI International; 2008.
59. **Kurtzman CP, Fell JW (ed).** *The Yeasts: A Taxonomic Study*, 4th ed. Elsevier; 1998.
60. **Robert V, Stegehuis G, Stalpers J.** The MycoBank engine and related databases. Available online at www.mycobank.org (accessed: 4 October 2024 [TO BE UPDATED FURTHER])
61. **Matthews REF.** Classification and Nomenclature of Viruses. Fourth Report of the International Committee on Taxonomy of Viruses. *Intervirology* 1982;17:1–200.
62. **Wildy P (ed).** *Classification and Nomenclature of Viruses. First Report of the International Committee on Nomenclature of Viruses*. Basel: S. Karger; 1971.
63. Species 2000 & ITIS. Catalogue of Life, www.catalogueoflife.org (accessed: 4 October 2024 [TO BE UPDATED FURTHER])]. Naturalis, Leiden, the Netherlands.
64. **Chun J, Oren A, Ventosa A, Christensen H, Arahal DR, *et al*.** Minimal standards for the use of genome data for the taxonomy of prokaryotes. *Int J Syst Evol Microbiol* 2018;68:461-466.
65. **Logan NA, Berge O, Bishop AH, Busse H-J, De Vos P, *et al*.** Proposed minimal standards for describing new taxa of aerobic, endospore-forming bacteria. *Int J Syst Evol Microbiol* 2009;59:2114–2121.
66. **Imhoff JF, Caumette P.** Recommended standards for the description of new species of anoxygenic phototrophic bacteria. *Int J Syst Evol Microbiol* 2004;54:1415-1421.
67. **Mattarelli P, Holzapfel W, Franz CMAP, Endo A, Felis GE, *et al*.** Recommended minimal standards for description of new taxa of the genera *Bifidobacterium*, *Lactobacillus* and related genera. *Int J Syst Evol Microbiol* 2014;64:1434–1451.
68. **Corbel MJ, Brinley Morgan WJ.** Proposal for minimal standards for descriptions of new species and biotypes of the genus *Brucella*. *Int J Syst Bacteriol* 1975;25:83–89.

69. **Corbel MJ, Brinley Morgan WJ.** Proposal for minimal standards for descriptions of new species and biotypes of the genus *Brucella* (erratum). *Int J Syst Bacteriol* 1975;25:243.
70. **Ursing JB, Lior H, Owen RJ.** Proposal of minimal standards for describing new species of the family *Campylobacteraceae*. *Int J Syst Bacteriol* 1994;44:842–845.
71. **On SLW, Miller WG, Houf K, Fox JG, Vandamme P.** Minimal standards for describing new species belonging to the families *Campylobacteraceae* and *Helicobacteraceae*: *Campylobacter*, *Arcobacter*, *Helicobacter* and *Wolinella* spp. *Int J Syst Evol Microbiol* 2017;67:5296-5311.
72. **Bernardet J-F, Nakagawa Y, Holmes B, Subcommittee on the taxonomy of *Flavobacterium* and *Cytophaga*-like bacteria of the International Committee on Systematics of Prokaryotes.** Proposed minimal standards for describing new taxa of the family *Flavobacteriaceae* and emended description of the family. *Int J Syst Evol Microbiol* 2002;52:1049–1070.
73. **Cui H-L, Amoozegar MA, Dyall-Smith ML, de la Haba RR, Minegishi H, *et al*.** Proposed minimal standards for description of new taxa of the class *Halobacteria*. *Int J Syst Evol Microbiol* 2024;74:005290.
74. **Arahal DR, Vreeland RH, Litchfield CD, Mormile MR, Tindall BJ, *et al*.** Recommended minimal standards for describing new taxa of the family *Halomonadaceae*. *Int J Syst Evol Microbiol* 2007;57:2436–2446.
75. **Arahal DR, Vreeland RH, Litchfield CD, Mormile MR, Tindall BJ, *et al*.** Recommended minimal standards for describing new taxa of the family *Halomonadaceae*. Erratum. *Int J Syst Evol Microbiol* 2008;58:2673.
76. **Dewhirst FE, Fox JG, On SL.** Recommended minimal standards for describing new species of the genus *Helicobacter*. *Int J Syst Evol Microbiol* 2000;50:2231–2237.
77. **Boone DR, Whitman WB.** Proposal of minimal standards for describing new taxa of methanogenic bacteria. *Int J Syst Bacteriol* 1988;38:212–219.
78. **Schumann P, Kämpfer P, Busse H-J, Evtushenko LI, for the Subcommittee on the Taxonomy of the Suborder *Micrococcineae* of the International Committee on Systematics of Prokaryotes.** Proposed minimal standards for describing new genera and species of the suborder *Micrococcineae*. *Int J Syst Evol Microbiol* 2009;59:1823–1849.
79. **International Committee on Systematic Bacteriology Subcommittee on the Taxonomy of Mollicutes.** Proposal of minimal standards for descriptions of new species of the class *Mollicutes*. *Int J Syst Bacteriol* 1979;29:172–180.
80. **Brown DR, Whitcomb RF, Bradbury JM.** Revised minimal standards for description of new species of the class *Mollicutes* (division *Tenericutes*). *Int J Syst Evol Microbiol* 2007;57:2703–2719.
81. **Whitcomb RF.** Evolution and devolution of minimal standards for descriptions of species of the class *Mollicutes*: analysis of two *Spiroplasma* descriptions. *Int J Syst Evol Microbiol* 2007;57:201–206.
82. **Bøvre K, Henriksen SD.** Minimal standards for description of new taxa within the genera *Moraxella* and *Acinetobacter*: Proposal by the Subcommittee on *Moraxella* and Allied Bacteria. *Int J Syst Bacteriol* 1976;26:92–96.
83. **Lévy-Frébault VV, Portaels F.** Proposed minimal standards for the genus *Mycobacterium* and for description of new slowly growing *Mycobacterium* species. *Int J Syst Bacteriol* 1992;42:315–323.
84. **International Committee on Systematic Bacteriology Subcommittee on the Taxonomy of *Mycoplasmatales*.** Minutes of Meeting, 5 and 6 September 1973, Jerusalem, Israel. *Int J Syst Bacteriol* 1972;22:184–188.

85. **Christensen H, Kuhnert P, Busse H-J, Frederiksen WC, Bisgaard M**. Proposed minimal standards for the description of genera, species and subspecies of the *Pasteurellaceae. Int J Syst Evol Microbiol* 2007;57:166–178.
86. **de Lajudie PM, Andrews M, Ardley J, Eardly B Jumas-Bilak E, *et al.*** Minimal standards for the description of new genera and species of rhizobia and agrobacteria. *Int J Syst Evol Microbiol* 2019;69:1852-1863.
87. **Graham PH, Sadowsky MJ, Keyser HH, Barnet YM, Bradley RS, *et al.*** Proposed minimal standards for the description of new genera and species of root- and stem-nodulating bacteria. *Int J Syst Bacteriol* 1991;41:582–587.
88. **Freney J, Kloos WE, Hajek V, Webster JA, Bes M, *et al.*** Recommended minimal standards for description of new staphylococcal species. *Int J Syst Bacteriol* 1999;49:489–502.
89. **Young JM, Bradbury JF, Gardan L, Gvozdyak RI, Stead DE, *et al.*** Comment on the Reinstatement of *Xanthomonas citri* (ex Hasse 1915) Gabriel et al. 1989 and *X. phaseoli* (ex Smith 1897) Gabriel et al. 1989: Indication of the Need for Minimal Standards for the Genus *Xanthomonas. Int J Syst Bacteriol* 1991;41:172–177.
90. **Oren A.** How to name new taxa of archaea and bacteria. In: Bergey's Manual of Systematics of Archaea and Bacteria. John Wiley & Sons, in association with Bergey's Manual Trust; 2019. https://doi.org/10.1002/9781118960608.bm00008.pub2 (accessed: 4 October 2024 [TO BE UPDATED FURTHER])
91. **Trüper HG.** How to name a prokaryote? Etymological considerations, proposals and practical advice in prokaryote nomenclature. *FEMS Microbiol Rev* 1999;23:231–249.
92. **Arahal DA, Bull CT, Busse H-J, Christensen H, Chuvochina M, Dedysh SN, *et al.*** Guidelines for interpreting the International Code of Nomenclature of Prokaryotes and for preparing a Request for an Opinion. *Int J Syst Evol Microbiol* 3034;73:005782.
93. **Trüper HG, Euzéby JP.** International Code of Nomenclature of Prokaryotes. Appendix 9: Orthography. *Int J Syst Evol Microbiol* 2009;59:2107–2113.
94. **Buchanan RE.** Chemical terminology and microbiological nomenclature. *Int Bull Bacteriol Nomencl Taxon* 1960;10:16-22; Reprinted: *Int J Syst Bacteriol* 1994;44:588–590.
95. **MacAdoo TO.** Nomenclatural literacy. In: Goodfellow M and O'Donnell AG (eds). *Handbook of New Bacterial Systematics.* London: Academic Press; 1993. pp. 339–358.
96. **Oren A.** Proposal to change Recommendation 12c of the International Code of Nomenclature of Prokaryotes. *Int J Syst Evol Microbiol* 2015;65:4288.
97. **Oren A, Schink B.** Formation of names of genera of prokaryotes that end on –*oides* or –*opsis*: A proposal for addenda to Rule 65(2) and to Appendix 9 of the International Code of Nomenclature of Prokaryotes. *Int J Syst Evol Microbiol* 2016;66:2452–2453.
98. **Oren A, Schink B.** Use of Greek in prokaryotic nomenclature: proposal to change Principle 3, Recommendation 6, Rule 7, Rule 65 and Appendix 9 of the International Code of Nomenclature of Prokaryotes. *Int J Syst Evol Microbiol* 2020;70:3559–3560.
99. **Oren A, Schink B.** Further guidelines for the formation of compound specific and subspecific epithets. A proposal to emend Appendix 9 of the International Code of Nomenclature of Prokaryotes. *Int J Syst Evol Microbiol* 2020;70:3561–3562.
100. **Oren A, Vandamme P, Schink B.** Notes on the use of Greek roots in the genus and species names of prokaryotes. *Int J Syst Evol Microbiol* 2016;66:2129–2140.
101. **Oren A, Garrity GM, Schink B, Ventura S.** 'Localimania' revisited: guidelines for the formation of specific epithets for names of prokaryotes based on names of institutions or their acronyms. A proposal for emendation of Appendix 9 to the International Code of Nomenclature of Prokaryotes. *Int J Syst Evol Microbiol* 2017;67:1618–1619.

102. Oren A, Chuvochina M, Schink B. The use of Greek and Latin prepositions and prefixes in compound names: proposed emendation of Appendix 9 of the International Code of Nomenclature of Prokaryotes. *Int J Syst Evol Microbiol* 2019;69:1831–1832.

103. Oren A, Chuvochina M, Ventura S. Formation of compound generic names based on personal names: a proposal for emendation of Appendix 9 of the International Code of Nomenclature of Prokaryotes. *Int J Syst Evol Microbiol* 2019;69:594–596.

104. Trüper HG. Help! Latin! How to avoid the most common mistakes while giving Latin names to newly discovered prokaryotes. *Microbiologia* 1996;12:473–475.

105. Trüper HG. How to name a prokaryote? Etymological considerations, proposals and practical advice in prokaryote nomenclature. *FEMS Microbiol Rev* 1999;23:231–249.

106. Trüper HG. Etymology in nomenclature of procaryotes. In: Boone DR, Castenholz RW and Garrity GM. (eds) *Bergey's Manual of Systematic Bacteriology*, 2nd edn, vol. 1, New York: Springer; 2001. pp. 89–99.

107. Trüper HG. The use of Neolatin in biological nomenclature. *Neulateinisches Jahrb J Neo-Latin Lang Lit* 2004;6:318–327.

108. Trüper HG. Is 'localimania' becoming a fashion for prokaryote taxonomists? *Int J Syst Evol Microbiol* 2005;55:1753.

109. Murray RGE, Schleifer KH. Taxonomic notes: a proposal for recording the properties of putative taxa of procaryotes. *Int J Syst Bacteriol* 1994;44:174–176.

110. Judicial Commission of the IJSB. Minutes of the meetings, 2 and 6 July 1994, Prague, Czech Republic. *Int J Syst Bacteriol* 1995;45:195–196.

111. Murray RE, Stackebrandt E. Taxonomic Note: Implementation of the provisional status *Candidatus* for incompletely described procaryotes. *Int J Syst Bacteriol* 1995;45:186–187.

112. Oren A. A plea for linguistic accuracy – also for *Candidatus* taxa. *Int J Syst Evol Microbiol* 2017;67:1085–1094.

113. Whitman WB. Modest proposals to expand the type material for naming of prokaryotes. *Int J Syst Evol Microbiol* 2016;66:2108–2112.

114. Whitman WB, Sutcliffe IC, Rossello-Mora R. Proposal for changes in the International Code of Nomenclature of Prokaryotes: granting priority to *Candidatus* names. *Int J Syst Evol Microbiol* 2019;69:2174–2175.

115. Sutcliffe IC, Dijkshoorn L, Whitman WB, on behalf of the ICSP Executive Board. Minutes of the International Committee on Systematics of Prokaryotes online discussion on the proposed use of gene sequences as type for naming of prokaryotes, and outcome of vote. *Int J Syst Evol Microbiol* 2020;70:4416–4417.

116. Oren A, Garrity GM, Parker CT, Chuvochina M, Trujillo ME. Lists of names of prokaryotic Candidatus taxa. Candidatus List no. 1. *Int J Syst Evol Microbiol* 2020;70:3956–4042.

117. Oren A, Garrity GM, Trujillo ME. Registration of names of prokaryotic Candidatus taxa in the IJSEM. *Int J Syst Evol Microbiol* 2020;70:3955.

118. Oren A, Garrity GM. Lists of names of prokaryotic Candidatus taxa. Candidatus List no. 2. *Int J Syst Evol Microbiol* 2021;71:004671.

119. Oren A, Garrity GM. Lists of names of prokaryotic Candidatus taxa. Candidatus List no. 3. *Int J Syst Evol Microbiol* 2022;72:005186.

120. Oren A. Lists of names of prokaryotic *Candidatus* taxa. Candidatus List no. 4. *Int J Syst Evol Microbiol* 2022;72:005545.

121. Oren A, Göker M. *Candidatus* List. Lists of names of prokaryotic *Candidatus* phyla. *Int J Syst Evol Microbiol* 2023;73:005821.

122. **Sutcliffe IC, Whitman BW, on behalf of the ISCP.** [sic] The van Niel International Prize for Studies in Bacterial Systematics, awarded in 2020 to Tanja Woyke. *Int J Syst Evol Microbiol* 2020;70:5594–5595.
